# Symbiotic Virus at the Evolutionary Intersection of Three Types of Large DNA Viruses; Iridoviruses, Ascoviruses, and Ichnoviruses

**DOI:** 10.1371/journal.pone.0006397

**Published:** 2009-07-28

**Authors:** Yves Bigot, Sylvaine Renault, Jacques Nicolas, Corinne Moundras, Marie-Véronique Demattei, Sylvie Samain, Dennis K. Bideshi, Brian A. Federici

**Affiliations:** 1 Génétique, Immmunothérapie, Chimie et Cancer, UMR CNRS 6239, Université François Rabelais de Tours, UFR des Sciences et Techniques, Parc de Grandmont, Tours, France; 2 INRIA/IRISA, Centre de Rennes Bretagne-Atlantique, Campus de Beaulieu, Rennes, France; 3 GENOSCOPE, Evry, France; 4 Department of Natural and Mathematical Sciences, California Baptist University, Riverside, California, United States of America; 5 Department of Entomology & Graduate Programs in Genetics & Microbiology, University of California Riverside, Riverside, California, United States of America; Georgia Institute of Technology, United States of America

## Abstract

**Background:**

The ascovirus, DpAV4a (family *Ascoviridae*), is a symbiotic virus that markedly increases the fitness of its vector, the parasitic ichneumonid wasp, *Diadromus puchellus*, by increasing survival of wasp eggs and larvae in their lepidopteran host, *Acrolepiopsis assectella*. Previous phylogenetic studies have indicated that DpAV4a is related to the pathogenic ascoviruses, such as the *Spodoptera frugiperda* ascovirus 1a (SfAV1a) and the lepidopteran iridovirus (family *Iridoviridae*), *Chilo* iridescent virus (CIV), and is also likely related to the ancestral source of certain ichnoviruses (family *Polydnaviridae*).

**Methodology/Principal Findings:**

To clarify the evolutionary relationships of these large double-stranded DNA viruses, we sequenced the genome of DpAV4a and undertook phylogenetic analyses of the above viruses and others, including iridoviruses pathogenic to vertebrates. The DpAV4a genome consisted of 119,343 bp and contained at least 119 open reading frames (ORFs), the analysis of which confirmed the relatedness of this virus to iridoviruses and other ascoviruses.

**Conclusions:**

Analyses of core DpAV4a genes confirmed that ascoviruses and iridoviruses are evolutionary related. Nevertheless, our results suggested that the symbiotic DpAV4a had a separate origin in the iridoviruses from the pathogenic ascoviruses, and that these two types shared parallel evolutionary paths, which converged with respect to virion structure (icosahedral to bacilliform), genome configuration (linear to circular), and cytopathology (plasmalemma blebbing to virion-containing vesicles). Our analyses also revealed that DpAV4a shared more core genes with CIV than with other ascoviruses and iridoviruses, providing additional evidence that DpAV4a represents a separate lineage. Given the differences in the biology of the various iridoviruses and ascoviruses studied, these results provide an interesting model for how viruses of different families evolved from one another.

## Introduction

Despite advances in understanding the evolutionary history of organisms made possible by molecular phylogenetics, the origins of most viruses and their radiation during evolution remain very poorly understood. This is due to the enormous diversity of virus types, ranging from those that produce very small virions, less than 20 nm in diameter consisting of a single-stranded genome of 2 kbp and protein coat, to those with large and complex enveloped virions, 300 to greater than 1,000 nm in diameter, containing fifty or more proteins with double-stranded DNA genomes ranging from 200 to greater than 1,000 kbp [1]. Polioviruses and parvoviruses are examples of the former, whereas poxviruses, iridoviruses, and mimiviruses are examples of the latter. This diversity suggests that unlike organisms, viruses are polyphyletic, with many, if not most types having originated independently. Complicating the evolutionary history of viruses is the evidence that many of the most complex types evolved by acquiring and exchanging genes with their hosts as they evolved [2]. For these reasons, the highest taxonomic classification for the thousands of recognized viral species is at the level of the family, of which there are currently about seventy [3].

Recent molecular phylogenetic studies of virus families that produce enveloped virions with large double-stranded DNA genomes suggest, however, that several of these, namely, the phycodnaviruses (family *Phycodnaviridae*), asfarviruses (family *Asfarviridae*), iridoviruses (family *Iridoviridae*) and ascoviruses (family *Ascoviridae*), are related and likely share a common evolutionary history. Based on studies of genes coding for proteins such as the major capsid protein, DNA polymerase, and thymidine kinase, these studies indicate that the asfarviruses and iridoviruses evolved from the phycodnaviruses, and the ascoviruses evolved from iridoviruses, in the latter case most likely iridoviruses that infect lepidopteran hosts [4]. Molecular evidence has also been provided recently that the viruses of the genus *Ichnovirus*, family *Polydnaviridae*, evolved from ascoviruses [5], [6].

Whereas the viruses of these families share certain structural similarities among their virions, they differ markedly in properties such as host range, cellular pathology, and transmission, making their evolutionary history of particular interest. For example, the phycodnaviruses attack eukaryotic algae and protozoans where they replicate and assemble in the cytoplasm [7] whereas the asfarviruses are a small group of cytoplasmic viruses known only from swine [8]. The iridoviruses compose a large family containing many species that differ broadly in host range, some reported from vertebrates, including salamanders and fish, whereas most are known from invertebrates, including nematodes and especially insects [9]. Phycodnavirus, asfarviruses, and iridoviruses all produce virions that are icosahedral. Alternatively, the ascoviruses and ichnoviruses produce virions that are bacilliform or reniform, and are only known from insects, specifically lepidopterans and hymenopterans, where they have relationships that range from being, respectively, highly pathogenic to symbiotic. The virions of both types are transmitted by female parasitic wasps during oviposition. Ascoviruses cause a fatal disease in their lepidopteran larval and pupal hosts, in which after the virions invade the nuclei of various tissues, the cells undergo a cellular pathology resembling apoptosis. During this process, the developing apoptotic bodies are rescued and form vesicles in which virions are assembled and disseminated. On the other hand, ichnovirus virions, the genomes of which are fully encoded in the chromosomes of the parasitic wasps that transmit them, invade the nuclei of their lepidopteran hosts, but never replicate or produce progeny virions in these. Instead, the function of ichnovirus virions is to deliver wasps genes that encode immunosuppressive proteins into their lepidopteran hosts to enhance survival of wasps eggs and progeny [10].

The significant differences in the biology of the iridoviruses, ascoviruses, and ichnoviruses are representative of major changes in their genomes, changes that likely began with iridoviruses that infect lepidopteran larvae. It is known, for example, that the endoparasitic wasp, (*Eiphosoma vitticolle*), can mechanically transmit on its ovipositor an iridovirus that infects its lepidopteran host, *Spodoptera frugiperda*, as well as this parasite's larvae developing in this host [11]. The transmission of most ascoviruses is similar to this in that their virions are transmitted mechanically on the ovipositor of parasitic wasps that parasitize caterpillars. Most ascoviruses, such as the type species, the *Spodoptera frugiperda ascovirus 1a* (SfAV1a), do not infect larvae or adults of their wasps vectors. There is, however, a very notable and important exception, the *Diadromus puchellus ascovirus 4a* (DpAV4a). This virus infects, but without causing any pathology, males and females of its ichneumonid wasp vector, *Diadromus puchellus* [12]. Interestingly, the DpAV4a genome resides as an unintegrated circle in the nuclei of all cells, providing a possible example of an evolutionary intermediate that led to the origin of ichnoviruses. During oviposition, DpAV4a virions are transmitted vertically to its pupal lepidopteran host, *Acrolepiopsis assectella*, where the virus kills the host as it replicates in most tissues. As with ichnoviruses, DpAV4a suppresses the host defence response thereby enabling parasite development [12], [13]. Wasp larvae ingest progeny virions as they develop, and this, plus vertical transmission through the egg, are the apparent mechanisms by which DpAV4a is maintained in the *D. puchellus* population.

The unique biology of the ascoviruses, and especially DpAV4a, suggested that the genome of this virus could provide significant insights into the evolutionary history of the apparent transition from the iridoviruses to the ascoviruses and ichnoviruses. Thus we sequenced the DpAV4a genome, and report here the phylogenetic analysis of 28 of its core genes. This analysis indicates that DpAV4a had a separate origin in the iridoviruses from the other ascoviruses, but evolved in parallel with these. Furthermore, our data suggest that other ascovirus-like particles, including the virions of ichnoviruses and other virus-like particles involved in the suppression of host innate immunity likely evolved from evolutionary changes that occurred among various iridoviruses infecting insects. These results suggest that molecular phylogenetic studies of viruses belonging to these families provide a rich source of material for studying how viruses evolve.

## Results

### Features of the DpAV4a genome

#### Genome properties

The DpAV4a genome consisted of a circular double-stranded (ds) DNA molecule of 119,343 bp with a G + C content of 49.66%. These traits are within the range of ascovirus and iridovirus genomes, which, respectively, vary from 90 to 215 kbp and 27.25 to 54.8% G + C [14]–[17]. Previously it was shown that the DpAV4a genome had a significant number of 5-methyldeoxycytidines [18] when this virus replicated in its lepidopteran pupal host, and therefore the frequency of CpX or XpC dinucleotides were investigated to verify whether they were subjected to unexpected increases or decreases. Our calculations revealed that there was no CpX or XpC shortage, indicating that the occurrence of 5-methyldeoxycytidine did not create a mutational bias in the DpAV4a genome.

Previous restriction fragment length polymorphism (RFLP) studies of the DpAV4a genome demonstrated that this virus was polymorphic in natural populations [12]. In the present study of the isolate sequenced, which was a mixture of variants, we detected 17 positions distributed along the genome that were highly polymorphic (variable in more than 20% of the reads; *Fig. S1a*), all of which were silent or neutral nucleotide (nt) substitutions. This indicated that the nt polymorphism rate was 0.015% among the DpAV4a variants within the isolate sequenced. We also compared the DpAV4a genome sequence to several other fragments of this viral genome cloned and sequenced from other isolates of this species (*Fig. S1b*; [4], [12], [18]). In agreement with our RFLP data, we found that sequence polymorphism rates among the variants within these other DpAV4a isolates ranged from 0.8 to 4.1%.

#### ORF content

**Figure 1:**
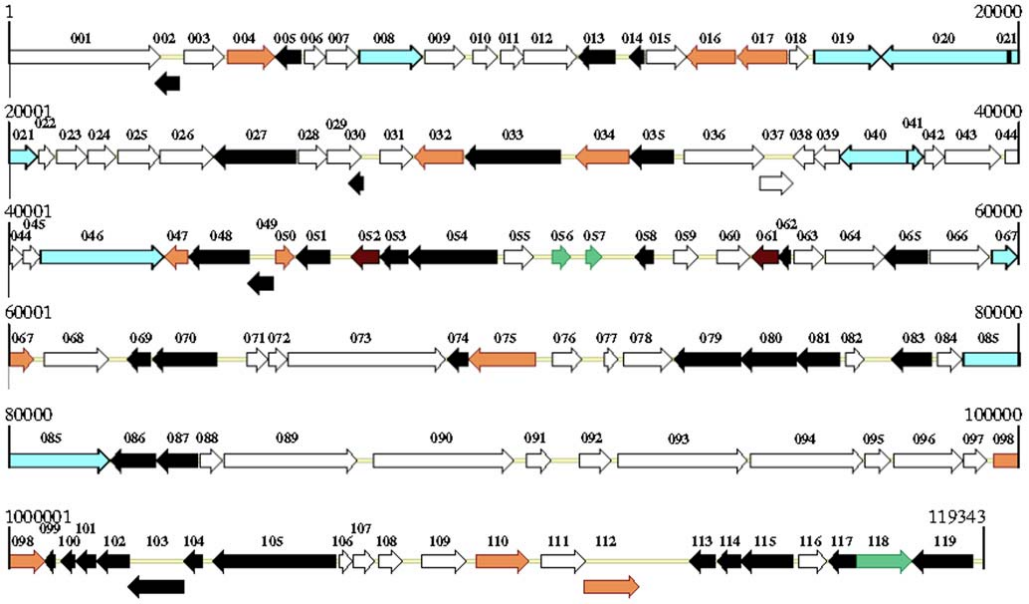
Schematic illustration of the organization of the *Diadromus pulchellus ascovirus 4a* (DpAV4a) genome. Predicted ORFs are indicated by their location, orientation, and putative size. White arrows represent ORFs on the forward strand, whereas black arrows identify those on the reverse strand. ORFs corresponding to members of repeated protein families are light orange for the BRO-like proteins, green for the metallo-hydrolases, and purple for the ABC-type transport system permease. ORFs encoding peptides present in TnAV2a and SfAV1a virions are light blue. The DpAV4a genomic map was constructed using Vector NTI (Invitrogene).

A total of 433 open reading frames (ORFs) with a methionine start codon and a minimum protein size of 50 amino acids were identified in the DpAV4a genome. Among these, 119 ORFs with no or minimal overlap (≤150 nt) were assumed to encode putative proteins (Fig. 1; *Fig. S2*). In agreement with previous sequencing studies of ascovirus genomes [15]–[17], the A of the ATG start codon of the ORF encoding the DNA polymerase B was arbitrarily assigned position 1 for the DpAV4a genome. The predicted ORFs were not distributed equally on both strands; 71 were in forward orientation whereas 48 were in the reverse orientation, with many of these being arranged in unidirectional gene clusters. ORFs represent 88.5% of the genome with an average density of one gene per 887 bp. We detected a linear relationship between the ORF number and genome size among most ascovirus and iridovirus genomes (*Fig. S3*; R^2^ = 0.757), with the exceptions being the genomes of MIV [19] and SfAV1a [15]. In MIV, this was due primarily to the presence of fifteen large repeats representing about 20% of its genome. In SfAV1a, this is due to the presence of (a) two large non-coding inverted repeats (7.4% of the genome), (b) an ORF65bis (*Fig. S4a*; [6]), and (c) to 61 overlapping ORFs described previously [15], named ORF A to OOO (*Fig. S4b*), but not referenced in Genebank. We found that 26 of the SfAV1a ORFs were present in the HvAV3e genome, and 10 of the same in the TnAV6a genome. In the DpAV4a genome, we determined that the ORF023 is a homologue of the SfAV1a ORF R, and that ORFs 056 and 057 are homologues of the SfAV1a ORF P. Interestingly, we found no homologue for each of these 61 SfAV1a ORFs among the ORFs contained in vertebrate and invertebrate iridovirus genomes. This suggested, therefore, that these 26 SfAV1a ORFs were characteristic of ascovirus genomes. Taking into account these data and errata (*Fig. S4c*), we adjusted the genomes sizes and ORF number of MIV and SfAV1a accordingly, which yielded a very significant linear correlation (R^2^ = 0.9265) between ORF number and the genome size of these two viruses (*Fig. S3*).

#### Coding capacities

BlastP and tBlastN searches revealed that only 26 of the 119 DpAV4a ORFs were “orphans;” the 93 others had homologues in the NCBI protein databases. Among these 93, 21 had similarities with viral proteins with no assigned function. The 72 others encoded proteins that had similarities strong enough (e values<0.01), conserved motifs and domains in specific databases, to assign each of these a putative function (*Fig. S2*). For most proteins coded for by these ORFs, their roles in virus metabolism, including such functions as DNA replication, recombination, transcription, protein modification, and apoptosis were described previously for iridoviruses and ascoviruses [15]–[17], [19], [20].

Four other features of ORFs in the DpAV4a genome, however, deserve special mention. In the first case, we used recent analyses of proteins present in the virions of SfAV1a [21] and TnAV6a [22] to search for homologues in the DpAV4a genome (*Fig. S5*). This search revealed that 10 of the 21 proteins found in the SfAV1a virion had homologues in DpAV4a, and also in the genomes of HvAV3e, TnAV6a, and the *Chilo* iridovirus (CIV). An additional protein, DpAV4a ORF063, was found only in ascovirus genomes. This suggested that there were 10 virion proteins in common to all ascoviruses and iridoviruses, and 11 present among all ascovirus virions.

The second interesting trait of the DpAV4a genome is the presence of two loci that each have a palindromic sequence over 90 and 150 bp (positions 55460 to 55550 and 90700 to 90850) within ORFs 062 and 091. RNA Mfold calculations showed that these were capable of forming very stable hairpin RNA structures (*Fig. S2*), suggesting that miRNAs could potentially be produced from the hairpins. Other miRNAs have been described recently for certain herpesvirus and HvAV3e genes [23], [24]. Interestingly, these two DpAV4a ORFs encode, respectively, ubiquitin and a homologue of ichnovirus proteins D1, D3 and D4, which might be important factors in regulating the virus virulence or replication [25], [26].

The third genomic feature of interest in DpAV4a is the presence of ORFs 90, 91 and 93, which code for proteins unique to a pox-D5 NTPase protein family in the *Glypta fumiferanae* ichnovirus (GfIV; [27]). These proteins, as well as other structural features of the GfIV virion were suggested recently to be indicative of a possible close evolutionary relationship between DpAV4a and GfIV [6]. A similar observation was also made with respect to the virion proteins encoded by ORF 19 and 44 in DpAV4 and two other ichnovirus proteins [6]. Whereas the sequence features of these proteins support evolutionary links between DpAV4, ascoviruses and ichnoviruses, the number of homolog ORFs contained in the ichnovirus genome was significantly lower than between ascovirus, DpAV4 and iridoviruses. This is because ichnovirus genomes, and more generally polydnavirus genomes, consist not of viral genes, but wasp genes, packaged into ancient viral proteins, the encoding genes for which were integrated into the wasp genome by symbiogenesis [5]. Two recent papers dealt with this interesting example of lateral gene transfer between viruses and parasitic wasps [6], [28]

**Figure 2:**
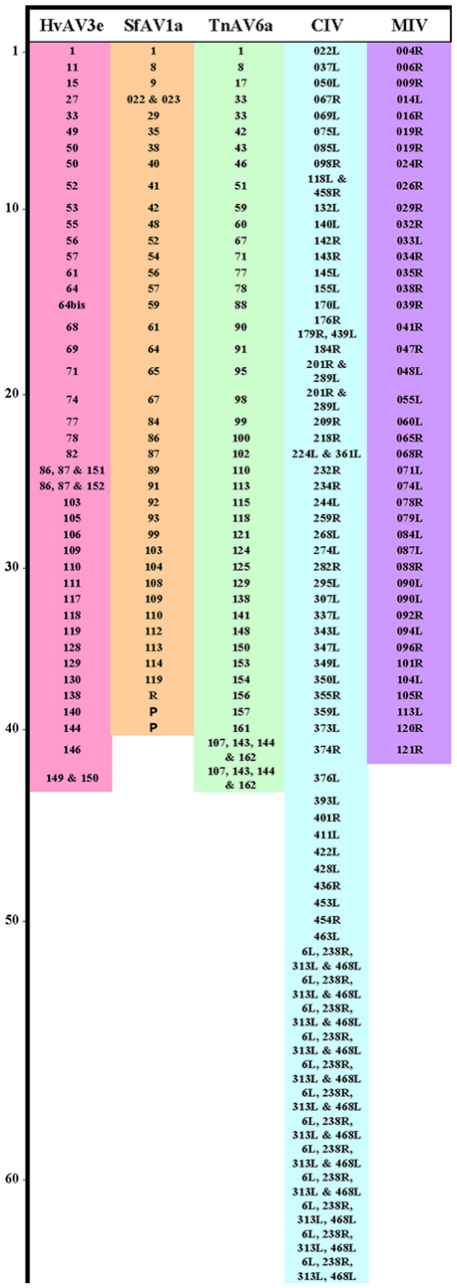
Graphic presentation of DpAV4a ORF homologues in the genomes of three ascoviruses (SfAV1a, TnAV6a, and HvAV3e) and two invertebrate iridoviruses (*Chilo* iridovirus, CIV; and Mosquito iridovirus, MIV).

The fourth feature, and the most surprising, is that 63 DpAV4a ORFs were homologues of ORFs in the CIV genome. This compares to only 42, 40, 42 and 41 shared by DpAV4a and, respectively, HvAV3e, SfAV1a, TnAV6a and mosquito iridovirus (MIV) genomes (Fig. 2). This finding suggested that DpAV4a was more closely related to the CIV than to any of the other ascoviruses or iridoviruses. Thus, we focused our analyses on the phylogenetic relationships of invertebrate iridoviruses, ascoviruses, and the unique DpAV4a, with the aim of identifying the number of core genes shared between and among these viruses.

### Evolutionary relationship of DpAV4a to iridoviruses and ascoviruses

#### Core genes

Identification of the number of core genes common to a virus family is a powerful tool used to define evolutionary relationships of virus families [29]. However, partial genomic data are less than optimal because gene sampling can be misleading in virus genomes containing genes of different origins, as exemplified in the *Glossina pallidipes* salivary gland hypertrophy virus (SHGV; [30]). For example, 28 of 160 genes of the SHGV genome display moderate similarities with baculoviruses (12) and entomopoxviruses (16) genes, findings that could lead to the conclusion that this virus is related to baculoviruses or entomopoxviruses, or even that it is a chimeric virus composed of portions of both virus types. Therefore, in our analyses we compared the entire gene content of ascovirus genomes, DpAV4a, and two insect iridoviruses, CIV and MIV. Our analysis of the closest DpAV4a ORF relatives revealed 28 core genes shared by all the sequenced ascoviruses, DpAV4a and iridoviruses (Table 1; *Fig. S6*). Our results did not confirm that ascoviruses originated from invertebrate iridoviruses, as proposed previously [4], but did indicate that they have a common origin. Our analyses of the conserved motifs, which putatively participate in regulating the expression through the 5′ and 3′ 150-bp regions of these ORFs, suggested that the mechanism of regulation was similar because the same sets of conserved nt motifs were present (*Fig. S7a,b,c*). Since only 26 core genes were shared by vertebrate and invertebrate iridoviruses, this suggested that the gene content of two invertebrate iridoviruses, MIV and CIV, was closer to those of the ascoviruses and DpAV4a (Table 2). This conclusion was supported by the finding that more core genes, 29, were shared by invertebrate iridoviruses and ascoviruses, or these viruses and DpAV4a, which shared 42, than with the vertebrate iridoviruses. Interestingly, only 34 core genes were shared by DpAV4a and SfAV1a, TnAV2a and HvAV3a, whereas the latter three ascoviruses shared at least 67 core genes. This indicated that the gene complement of DpAV4a was closer to that of the invertebrate iridoviruses than to other ascoviruses. Together, these data suggest that DpAV4a may not be an ascovirus. Instead, it may be a member of a new virus family that also originated from invertebrate iridoviruses, but from a root that would be different than that which led to the ascoviruses.

**Table 1:** Core genes common to the genomes of four ascoviruses and two invertebrate iridoviruses sequenced to date.

DpAV4a ORF N°	SfAV1a ORF N°	BLAST% similarity	HvAV3e ORF N°	BLAST% similarity	TnAV2c ORF N°	BLAST% similarity	CIV ORF N°	BLAST% similarity	MIV ORF N°	BLAST% similarity
**1**	**1**	**45**	**1**	**47**	**1**	**45**	**37L**	**55**	**120R**	**53**
**3**	**22 & 23**	**56**	**27**	**55**	**8**	**44**	**142R**	**57**	**101R**	**53**
***8***	***48***	***>20***	***61***	***>20***	***141***	***>20***	***232R***	***43***	***84L***	***>20***
9	57	35	69	>20	124	>20	359L	38	105R	>20
10	89	>20	138	>20	51	>20	454R	47	92R	>20
***19***	***41***	***41***	***56***	***42***	***153***	***46***	***274L***	***49***	***14L***	***47***
***20***	***9***	***51***	***15***	***53***	***161***	***52***	***22L***	***53***	***87L***	***52***
*22*	*91*	*49*	*130*	*>20*	*59*	*50*	*401R*	*62*	*68R*	*>20*
26	59	58	71	51	121	56	244L	63	78R	60
33	103	45	118	50	77	53	50L	50	94L	50
**36**	**104**	**41**	**117**	**44**	**88**	**48**	**98R**	**45**	**38R**	**44**
***40***	***35***	***39***	***52***	***39***	***157***	***44***	***118L & 458R***	***56–59***	***6R***	***51***
***41***	***61***	***50***	***74***	***47***	***118***	***54***	***347L***	***60***	***96R***	***54***
**43**	**65**	**41**	**78**	**42**	**113**	**45**	**393L**	**51**	**39R**	**44**
48	114	51	103	44	102	49	224L & 361L	50–52	24R	55
**55**	**40**	**57**	**55**	**50**	**154**	**50**	**143R**	**60**	**29R**	**57**
**64**	**56**	**>20**	**68**	**37**	**125**	**>20**	**67R**	**56**	**4R**	**60**
***65***	***54***	***40***	***65bis***	***58***	***129***	***42***	***337L***	***48***	***47R***	***46***
**70**	**67**	**48**	**82**	**48**	**110**	**50**	**343L**	**55**	**90L**	**45**
**73**	**52**	**59**	**64**	**59**	**138**	**60**	**428L**	**55**	**9R**	**56**
***85***	***84***	***37***	***146***	***37***	***43***	***35***	***295L***	***47***	***16R***	***45***
**86**	**110**	**66**	**109**	**64**	**95**	**65**	**75L**	**74**	**88R**	**67**
**89**	**8**	**53**	**11**	**53**	**42**	**54**	**176R**	**55**	**90L**	**53**
**93**	**99**	**41**	**119**	**49**	**78**	**47**	**184R**	**44**	**121R**	**46**
**103**	**29**	**50**	**33**	**49**	**17**	**52**	**282R**	**56**	**79L**	**54**
108	113	42	105	42	100	36	350L	48	26R	47
116	92	>20	129	>20	60	42	259R	55	71L	46
***117***	***109***	***52***	***110***	***48***	***90***	***50***	***355R***	***62***	***104L***	***55***

ORF number that were bolded correspond to 19 of the 26 core genes shared by these viruses with vertebrate iridoviruses. ORF65b is located from position 73288 to 74247 in the HvAV3e genome. ORF numbers typed in italics correspond to those encoding proteins that were present in the SfAV1a virion.

**Table 2:** Cores genes shared between and among viruses of the families *Iridoviridae*, *Ascoviridae* and DpAV4a.

Selected viruses	Number of core genes shared between members of each selection
Invertebrate iridoviruses, ascoviruses & DpAV4a	28
Vertebrates & invertebrate iridoviruses	26
Iridovirus, ascovirus & DpAV4a	20
Invertebrate iridoviruses & ascoviruses	29
Invertebrate iridoviruses & DpAV4a	42
Ascoviruses & DpAV4a	34 (1)
DpAV4a & CIV	63
Ascoviruses	67 (10)

Vertebrate iridoviruses = c.f. (12); Invertebrate iridoviruses = CIV + MIV; Ascoviruses = SfAV1a + TnAV2c + HvAV3e; DpAV4a. Values were calculated from literature [12] and *Fig. S6*. Unreferenced core genes presented in *Fig. S4* are indicated between parentheses.

#### Phylogenetic analyses of core genes

**Figure 3:**
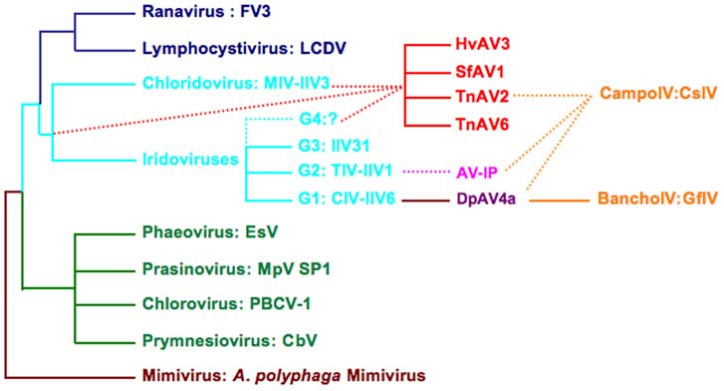
Synthesis of the evolutionary relationships among various genera of *Mimiviridae* (brown), *Phycodnaviridae* (green), *Iridoviridae* (invertebrate genera are in blue and vertebrate genera in dark blue), *Ascoviridae* (red), DpAV4a (purple) and ichnoviruses, *Polydnaviridae* (orange). The name of the principal virus representative of each genus is indicated. A putative family of ascovirus-like particles (AV-lP) are in pink. This synthetic tree was determined from results obtained with the evolutionary analyses of the 28 core genes and the literature [9], [29], [32]. Plain lines represent verified relationships. Dotted lines indicate evolutionary pathways between virus families that require further support for confirmation. Phylogenetic analyses of major capsid protein (MCP) sequences revealed that at least 3 groups (G1, G2, G3) occurred within the iridovirus genus. A hypothetical fourth one, G4, was used to support the putative origin of the *Ascoviridae*. Taxonomic details and full virus names in each virus family are available at: http://gicc.univ-tours.fr/collaborations/col_pics.php?connect=0&lang=fr or http://www.ncbi.nlm.nih.gov/ICTVdb/Ictv/fs_ascov.htm; http://www.ncbi.nlm.nih.gov/ICTVdb/Ictv/fs_irido.htm; http://www.ncbi.nlm.nih.gov/ICTVdb/ICTVdB/00.051.0.04.htm; http://www.ncbi.nlm.nih.gov/ICTVdb/Ictv/fs_mimiv.htm; http://www.ncbi.nlm.nih.gov/ICTVdb/Ictv/fs_asfar.htm; http://www.ncbi.nlm.nih.gov/ICTVdb/Ictv/fs_polyd.htm.

The evolutionary relationships of each of the 26 core genes shared by iridoviruses, ascoviruses and DpAV4a were determined using 20 vertebrate iridovirus homologues as outgroups (*Fig. S8a,c*), and phycodnavirus, mimivirus, eukaryotic or procaryotic homologues as outgroups for 8 others (*Fig. S8b,d*). For each gene, the phylogenetic tree obtained was expected to yield one of the three following results: i) no conclusion, in cases where the genes contained insufficient phylogenetic information; ii) support for the separate existence of two virus families having different evolutionary roots among iridoviruses, ascoviruses and DpAV4a; or iii) support to the existence of a single virus family, the ascoviruses, containing SfAV1a, TnAV2a, HvAV3a and DpAV4a. Results of this analysis revealed eleven genes that contained insufficient phylogenetic information to yield an informative answer. The other seventeen core genes, however, supported our hypothesis that DpAV4a was not an ascovirus, but rather a unique virus, more closely related to iridoviruses than ascoviruses, and thus likely a member of a new family with an origin different from that of the ascoviruses. None of the phylogenies obtained suggested that DpAV4a belonged to the same family as SfAV1a, TnAV6a and HvAV3a. A synthesis of our result is given in (Fig. 3).

## Discussion

We have reported here the sequence and phylogenetic analysis of the DpAV4a genome, identifying the major characteristics of this genome and comparing these with those of three other ascoviruses [15], [17], [22] and several invertebrate and vertebrate iridoviruses [14]. The number of homologues found between DpAV4a and these ascoviruses and iridoviruses, along with the phylogenetic results demonstrate that its closest relative is the invertebrate iridovirus CIV, not one of the known ascoviruses. These results suggest that DpAV4a is probably not an ascovirus, or at a minimum should be a member of a separate new genus of the family *Ascoviridae*. The results reported here, which are more detailed than prior phylogenetic analyses of the relationship between ascoviruses and invertebrate iridoviruses, also confirm that ascoviruses have a common ancestor. Interestingly and importantly, our analyses reveal that invertebrate iridoviruses are genetically and evolutionary closer to ascoviruses and DpAV4a, than to vertebrate iridoviruses. With respect to DpAV4a, our analyses suggest that this unique virus probably originates from an invertebrate iridovirus ancestor close to CIV, and evolved along a similar but different path parallel to the other non-symbiotic, pathogenic members of the family *Ascoviridae*, namely, SfAV1a, TnAV2a, and HvAV3a. It must be that our results currently only show that ascovirus and invertebrate iridoviruses have a common origin, and although strongly suggestive, and are not yet sufficiently consistent to definitively confirm the origin of ascoviruses from iridoviruses. This is because the genomic data available for iridoviruses, is very limited, and therefore very limited in our analyses. Indeed, only two genera of invertebrate iridoviruses have been recognized by the International Committee for Virus Taxonomy, the Iridovirus and the Chloriridovirus. Moreover, previous phylogenetic studies have proposed that there are at least three distinct iridovirus groups [32 and http://www.microbiologybytes.com/virology/kalmakoff/Iridoviruses.html]. CIV is currently a group II *Iridovirus* and MIV is the single member of *Chloriridovirus*. Thus, no genomic sequence data are currently available regarding the diversity within and among group I and group II iridoviruses. Sequencing and phylogenetic analyses of additional invertebrate iridovirus and ascovirus genomes will be required to define more precisely the evolutionary paths and relationships of these interesting large DNA viruses of invertebrates.

Our results also provide new insights into the possible origins of various ascovirus-like particles [33]–[37] and ichnoviruses [5], [6]. They suggest, for example, that some of the various ascovirus-like particles might not be ascoviruses, but rather members of other virus families that originated from invertebrate iridoviruses (Fig. 3). Though these results do not alter the concepts and final results with respect to the possibility and even probability that ichnoviruses originated from ascoviruses, analyses of our new molecular evidence improve the interpretation and support for an ascovirus origin of ichnoviruses [5], [6], either from a symbiotic ascovirus like DpAV4a or even an attenuated pathogenic ascovirus or ascovirus-like particle. Our molecular analyses indicating different but parallel evolutionary pathways for symbiotic and pathogenic ascoviruses accommodate the recent observation that ichnoviruses are not a monophyletic group [27], but without implying from which ascovirus lineage the apparently different lineages of ichnoviruses originated. For example, the morphology of ichnovirus virions and genomic properties of the viruses found in the wasp subfamilies, Banchinae and Campopleginae, were sufficiently different to propose that there are two unrelated ichnoviruses sub-families, the BanchoIV and the CampoIV [6]. The BanchoIV have properties suggesting they may be related to DpAV4a [6], but the origin of the CampoIV is not at all clear at present. Indeed, taking into account the lack of or low amount of molecular data, and the important rate of sequence divergence between certain CampoIV proteins [28], it cannot be determined whether their ancestral virus is located within one of the putative families of ascovirus-like particles or among ascoviruses (Fig. 3).

Three remarkable properties of the pathogenic and symbiotic ascoviruses have been acquired and exhibit convergence during their parallel evolution from different invertebrate iridovirus ancestors. First, with respect to virion structure, the virions of both evolved from an icosahedral symmetry to a reniform or bacilliform shape. Secondly, the genome configuration changed from a linear to a circular molecule. And thirdly, the cytopathology of viruses in both lineages evolved to produce of vesicles-containing virions from invaginations of the plasmalemma and *de novo* membrane synthesis in the infected host cells [1], [12]. The determination of the gene acquisitions and deletions that underlay these phenotypic characteristics of major importance, which have allowed to these viruses to be *a priori* so different from invertebrate iridoviruses, hold the possibility for providing important insights into how viruses evolved. Indeed, there are no other clearly related families of large DNA viruses among which such different morphological and genomic properties exist.

Moreover, in an even broader context, further phylogenetic studies of more distantly but still clearly related families based on molecular evidence, offers the possibility of even greater insights into virus evolution. For example, although virion shapes are different among the ascoviruses, iridoviruses, phycodnaviruses and mimiviruses, they all appear to contain two lipid membranes within a protein capsid composed primarily of a major capsid protein (MCP; [38]–[40]). In agreement with the sequence similarities of their MCPs, the CIV and phycodnavirus (PBCV-1) virions are similarly organized. To assemble their icosahedral capsids, MCP trimers were demonstrated to assemble in different oligomers, called n-symmetrons [41]. Thus, 20 triangular tri-symmetrons compose the 20 faces of the isocahedral virions, 12 penta-symmetrons are located at the particle vertices, and linear di-symmetrons are located at the lattices, playing the role of junctions between tri- and penta-symmetrons. Atomic force microscopy has revealed that two other viral proteins are located in the middle of the penta-symmetrons [42], putatively protein homologues to the PBCV-1 Vp260 (ORF A122R) and Vp280 [43]. Psi-BLAST mining of the databases revealed that ORFs encoding PBCV-1 Vp260 homologues are present in iridovirus genomes of CIV216R and MIV091L, but are absent in those of the ascoviruses. This therefore suggests that the loss of the gene encoding a Vp260 homologue in these viruses might prevent the assembly or the stability of penta-symmetrons. There is also one protein encoded by all ascovirus genomes, homologues of SfAV1a ORF038 and DpAV4a ORF063, that is present in the virions of these viruses, yet absent in iridoviruses and phycodnaviruses. A similar situation is also encountered with homologues of the SfAV1a ORF R and P. Although these two proteins were not detected in virions [21], they might be involved in virion assembly during virogenesis. Together, the loss of the ORF encoding Vp260 homologues and the acquisition of DpAV4a ORF063, and SfAV1a R and P homologues by lateral transfer are possible candidates to explain the change in oligomerisation of ascovirus major capsid proteins that resulted in the change of virion shape from an icosahedron to a basically bacilliform shape.

The circularization that occurred for the ascovirus genomes may have different origins. Indeed, the genome circularization in DpAV4 might result from the nuclease and ligase activities of proteins encoded by the ORFs 077 and 113, which have no homologues in ascovirus and iridovirus genomes. Similarly, in the pathogenic ascoviruses it might have been due to recombinase and nuclease activities of proteins encoded by the ORFs SfAV1a 066 and 075, which have no homologues among DpAV4a and iridovirus genomes.

Data recently published [44], [45] on the CIV cytopathology and the apoptosis regulation of the host cells revealed that dynamic changes occurred in the cytoskeleton of the CIV-infected cells, which lead to the differentiation and the release of vesicles. Unfortunately, published data does not yet indicate whether these vesicles contained virions. Nevertheless, they suggest that the cytopathology characteristic of ascoviruses and iridoviruses might not be determined by genes specifically present in the genome of these viruses. Instead, they may originate from a property shared by all invertebrate iridoviruses that would have been strongly differentiated during the evolutionary differentiation of ascoviruses from iridoviruses.

In conclusion, a more definitive elucidation of the ascovirus and ascovirus-like origins will require further efforts in phylogenetics. Nevertheless, it can be reasonably proposed that their significant phenotypic differences might result from evolutionary scenario involving a very limited number of gene losses and acquisitions.

## Materials and Methods

### DpAV4 DNA source

*D. pulchellus* is a solitary hymenopteran endoparasitoid that uses DpAV4a for successful parasitization of the leek-moth, *A. assectella* (Lepidoptera), which infests *Allium* species. DpAV4a stocks were maintained in the laboratory by breeding wasp and caterpillar hosts. Wasps were reared on host pupae as described (15, 38). Briefly, the wasps were reared in cages at 25°C, 60±10% relative humidity (RH) during the 16 h light period, and at 15°C, 70±10% RH during an 8 h dark period. Pupae of *A. assectella*, 24 h after pupation, were presented every day to *D. pulchellus* females for oviposition. The strain of *D. pulchellus* was established from adult wasps collected in September 1990, in southern France near the town of Antibes.

Total genomic DpAV4 DNA was produced and extracted as described (34). Pupae of *A. assectella* were inoculated using glass pins contaminated with hemolymph containing DpAV4a from pupae four days after being parasitized by wasps. Pupae infected with DpAV4a were held for 48 hours at 25°C for amplification of virus stocks (15).

### DNA sequencing and analysis of the DpAV4a genome

Sequencing strategy: The DpAV4a genome was sequenced using a shotgun strategy. Viral DNA was amplified using the rolling circle DNA polymerase TempliPhi (Amersham Biosciences). About 40 µg of DNA was sheared (Genemachines hydroshear) and the resulting fragments were separated on a preparative LPM agarose gel (FMC). DNA fragments of about 5-kbp were eluted using beta-agarase (Biolabs) and ligated into the *Bst*XI-digested pcDNA2.1 vector (Invitrogen). The transformation step was performed in electro-competent bacteria of the *Escherichia coli* strain DH10B (GIBCO-BRL). Sequence reads of 5000 subcloned ends were performed using dye terminator sequencing on an ABI 3700 (PE-Applied Biosystem). The sequences were assembled into contigs using PHRED [46] and PHRAP (phragment assembly program; P. Green, unpubl.) software. The gaps between the contigs were filled using primer walking, and poor quality sequences were improved using specific primers and dye terminator sequencing on automated ABI 3700 and ABI 3730 sequencers (PE-Applied Biosystem). Consensus sequences were considered as valid when at least 98% of the nts were base-called with a PHRAP score above 40. The consensus sequence was obtained after analysis of at least 10 sequence reads on both strands, or using sequencing methods based on two different labelling procedures applied to one strand.

### Genome analyses

Bioinformatics tools used for the present analyses were as described (17; Fig. S2).

### Phylogenetic analyses

The sequence alignments, determination of conserved blocks, and calculation of phylogenetic trees with the maximum likelihood, were obtained using MABL facilities at http://www.phylogeny.fr/phylo_cgi/downloads.cgi. Briefly, related sequences were determined using BLAST results from databases. They were then aligned using MUSCLE. Seqeunce alignment were curated using parameter options that allow i) smaller final blocks, ii) gap positions within the final blocks and iii) less strict flanking positions. Alignments were first used to calculate phylogenetic trees with the maximum likelihood procedure on the MABL site using the option “approximate likelihood-ratio test” to verify the consistence of the tree branching. Trees were automatically drawn with TreeDyn. Curated sequence alignments were also recovered for the MABL sites to calculate phylogenetic trees with the neighbor-joining, and parsimony procedures using WebPHYLIP facilities from http://www.genouest.org/spip.php?page=outils&id_rubrique=44.

### Data deposition footnotes

The nucleotide sequence of the DpAV4a genome of has been deposited in GenBank/EMBL/DDBJ database with the accession number (AM292540).

## Supporting Information

Figure S1 DpAV4a genomic polymorphisms. S1a: DNA polymorphism in the sequenced DpAV4a genome S1b: Sequence similarity between the DpAV4a sequenced genome and fragments previously cloned and sequenced from other DpAV4a isolates
(0.11 MB DOC)

Figure S2 Gene annotations in the DpAV4a genome
(0.90 MB DOC)

Figure S3 Relationship between the genome size and the number of ORFs of ascovirus, DpAV4a, vertebrate and invertebrate iridovirus genomes
(0.07 MB PDF)

Figure S4 Complementary information in gene annotation of previously sequenced ascovirus genomesS4a. Sequences of the proteins encoded, in the HvAV3e genome, by ORFs non-referenced in databases, and, having homologues in the other ascovirus genomes S4b. Unreferenced SfAV1a ORFs having homologues in the HvAV3e and TnAV2c genomes S4c. Sequence of the protein encoded, in the SfAV1a genome, by an ORF non-referenced in databases, and, having homologues in the other ascovirus genomes
(0.07 MB DOC)

Figure S5 SfAV1a virion proteins identified by mass spectrometry
(0.11 MB DOC)

Figure S6 ORF homologues between Ascoviruses and Iridoviruses
(0.20 MB DOC)

Figure S7 Conserved nucleotide motif in upstream and dowstream gene regions. S7a: A comparative summary of the transcription features of the 28 core genes shared by ascoviruses, DpAV4a and invertebrate iridoviruses, sequence analyses of DpAV4a, and data published for CIV. S7b: Conserved nt motifs in the 5′ UTR of the 28 core genes shared by ascoviruses, DpAV4a and invertebrate iridoviruses. S7c: Conserved nt motifs in the 3′ UTR of the 28 core genes shared by ascoviruses, DpAV4a and invertebrate iridoviruses.
(1.11 MB DOC)

Figure S8 Phylogenetic analysis of core genes. S8a. Phylogenetic trees depicting the relationships between each of the proteins shared by all vertebrate and invertebrate iridoviruses, ascoviruses and DpAV4a S8b. Phylogenetic trees depicting the relationships between each of the proteins shared by all invertebrate iridoviruses, ascoviruses and DpAV4a S8c. Table summarizing phylogenetic relationships between core proteins shared by all vertebrate and invertebrate iridoviruses, ascoviruses, and DpAV4a S8d. Table summarizing phylogenetic relationships between core proteins shared by all invertebrate iridoviruses, ascoviruses, and DpAV4a
(0.67 MB DOC)
