# Supplementary material for: Symbiotic Virus at the Evolutionary Intersection of Three Types of Large DNA Viruses; Iridoviruses, Ascoviruses, and Ichnoviruses
Source: PLoS One. 2009 Jul 28;4(7):e6397. doi: 10.1371/journal.pone.0006397 (PMC2712680; doi:10.1371/journal.pone.0006397)
Supplement: Figure S1 — DpAV4a genomic polymorphisms. S1a: DNA polymorphism in the sequenced DpAV4a genome S1b: Sequence similarity between the DpAV4a sequenced genome and fragments previously cloned and sequenced from other DpAV4a isolates (0.11 MB DOC) [file pone.0006397.s001.doc]

**S1 : Supporting Information 1**

**Symbiotic Virus at the Evolutionary Intersection of Three Types of Large DNA Viruses;**

**Iridoviruses, Ascoviruses, and Ichnoviruses**

Yves Bigot, Sylvaine Renault, Jacques Nicolas, Corinne Moundras, Marie-Véronique Demattei, Sylvie Samain, Dennis K. Bideschi, and Brian A. Federici

**S1a: DNA polymorphism in the sequenced DpAV4a genome**

**S1b: Sequence similarity between the DpAV4a sequenced genome and fragments previously cloned and sequenced from other DpAV4a isolates**

S1a: DNA polymorphisms in the DpAV4a genome sequenced

| **Position** | **Substitution** | **Nature** | **ORF location** |
| --- | --- | --- | --- |
| 21320 | G 🡪 C | Transversion | 23 |
| 30543 | C 🡪 G | Transversion | 33 |
| 63508 | T 🡪 C | Transition | Non-coding |
| 71721 | T 🡪 C | Transition | Non-coding |
| 71768 | T 🡪 C | Transition | Non-coding |
| 82217 | G 🡪 T | Transversion | 86 |
| 108604 | G 🡪 T | Transversion | 109 |
| 108610 | C 🡪 T | Transition | 109 |
| 108631 | A 🡪 G | Transition | 109 |
| 109415 | T 🡪 C | Transition | 110 |
| 109417 | G 🡪 C | Transversion | 110 |
| 109471 | C 🡪 G | Transversion | 110 |
| 109489 | A 🡪 C | Transversion | 110 |
| 109679 | T 🡪 C | Transition | 110 |
| 110098 | A 🡪 G | Transition | 110 |
| 111271 | C 🡪 A | Transversion | 111 |
| 111714 | G 🡪 A | Transition | 112 |

**S1b: Sequence similarity between the DpAV4a genome sequenced and fragments previously cloned and sequenced from other DpAV4a isolates**

| **Fragment AccN°** | **Fragment size in bp** | **Conserved regions between DpAV4a and each fragment** | **Similarity rate (%)** | **contained ORF** |
| --- | --- | --- | --- | --- |
| X85802 | 694 | 63360-64062 / 1-694 | 98.4 | 70 |
| X85803 | 738 | 88651-89401 / 1-738 | 97.1 | 90 |
| X85804 | 737 | 3886-4630 / 1-737 | 96.4 | 3 & 4 |
| X85806 | 1726 | 30810-32549 / 1-1726 | 98.0 | 34 |
| X85807 | 1585 | 107790-108169 / 1-1585 | 95.9 | Non coding |
| AJ279812 | 12255 | 117619-119334 / 1-1749  1-10519 / 1750-12255 | 99.0  98.4 | 118 and 119  1 to 11 |
| AJ279815 | 6736 | 32806-39542 / 1-6736 | 99.2 | 35 to 43 |
| AJ312705 | 1735 | 15737-17463 / 1-1735 | 97.5 | 18 to 20 |
| AJ312706 | 944 | 49778-50725 / 1-944 | 97.4 | 55 |
