# Supplementary material for: Symbiotic Virus at the Evolutionary Intersection of Three Types of Large DNA Viruses; Iridoviruses, Ascoviruses, and Ichnoviruses
Source: PLoS One. 2009 Jul 28;4(7):e6397. doi: 10.1371/journal.pone.0006397 (PMC2712680; doi:10.1371/journal.pone.0006397)
Supplement: Figure S2 — Gene annotations in the DpAV4a genome (0.90 MB DOC) [file pone.0006397.s002.doc]

**S2 : Supporting information 2**

**Symbiotic Virus at the Evolutionary Intersection of Three Types of Large DNA Viruses;**

**Iridoviruses, Ascoviruses, and Ichnoviruses**

Yves Bigot, Sylvaine Renault, Jacques Nicolas, Corinne Moundras, Marie-Véronique Demattei, Sylvie Samain, Dennis K. Bideschi, and Brian A. Federici

**Methods :** Genomic DNA composition, structure, and codon usage were analyzed using several desktop and online programs (DNASTAR (Lasergene); <http://www.info.univ-angers.fr/pub/gh/signets_gh.htm>, http://www.kazusa.or.jp/codon). Nucleotide and protein sequence database searches were performed using the BLAST programs at NCBI website ([http://www.ncbi.nlm.nih.gov](http://www.ncbi.nlm.nih.gov/)). For more refined analyses, conserved motifs and domains, and putative functions of deduced DpAV4a proteins composed of 49 or more amino acids with homology to other proteins in sequence databases were identified using several online programs, as follows:

*Physicochemical properties of the proteins*: <http://www.iut-arles.up.univ-mrs.fr/w3bb/d_abim/compo-p.html> and <http://www.expasy.ch/cgi-bin/protscale.pl>;

*Conserved motifs and domains*: <http://hits.isb-sib.ch/cgi-bin/hits_mpfsearch>, <http://www.expasy.org/cgi-bin/scanprosite>; <http://smart.embl-heidelberg.de/smart/set_mode.cgi?GENOMIC=1>; <http://www.cbs.dtu.dk/services/>; <http://npsa-pbil.ibcp.fr/cgi-bin/npsa_automat.pl?page=/NPSA/npsa_hmmbuild.html>, <http://www.ncbi.nlm.nih.gov/Structure/cdd/wrpsb.cgi>, <http://supfam.org/SUPERFAMILY/>, <http://idefix.univ-rennes1.fr:8080/GenOuest/outils.php3?id_rubrique=40>;

*Secretion and maturation motif predictions*: Non-classical and leaderless secretion of proteins: <http://www.cbs.dtu.dk/services/SecretomeP/>, Twin-arginine signal peptides: <http://www.cbs.dtu.dk/services/TatP-1.0/>, Peptide signal: <http://www.cbs.dtu.dk/services/SIGNALP/>, Transmembrane domains: TMHMM2.0 – <http://www.cbs.dtu.dk/services/TMHMM-2.0/>;

*Dimerization motifs*: WD domain repeat : <http://bmerc-www.bu.edu/bioinformatics/wd_search.html>;

*Leucine Zippers*: <http://2zip.molgen.mpg.de/index.html>;

*DNA binding motifs*: Zinc Finger domain : <http://www.sangamo.com/index.php> and <http://www.scripps.edu/mb/barbas/zfdesign/zfdesignhome.php>, Helix turn helix motif : <http://subaru2.univ-lemans.fr/sciences/lbge/MLEsdatabase/TOOLS/hth.php>, <http://npsa-pbil.ibcp.fr/cgi-bin/npsa_automat.pl?page=/NPSA/npsa_hth.html>;

*Post-translational modification* : Kinase specific eukaryotic protein phosphorylation sites -<http://www.cbs.dtu.dk/services/NetPhosK/> and <http://pred.ngri.re.kr/PredPhospho.htm>;

*Cellular location*: Subcellular location of proteins: mitochondrial, chloroplastic, secretory pathway, or other: <http://www.cbs.dtu.dk/services/TargetP/>, <http://motif.genome.jp/>, <http://www.cbs.dtu.dk/services/ProtFun/>, <http://elm.eu.org/>, <http://research.i2r.a-star.edu.sg/CysView/>, http://swift.cmbi.ru.nl/cyscys/web/;

*Cellular trafficking*: Nuclear localization signals : <http://subaru2.univ-lemans.fr/sciences/lbge/MLEsdatabase/TOOLS/nls.php> and <http://cubic.bioc.columbia.edu/cgi/var/nair/resonline.pl>; Leucine-rich nuclear export signals (NES) in eukaryotic proteins : http://www.cbs.dtu.dk/services/NetNES/;

*Disulfide bridge*: http://www.info.univ-angers.fr/pub/richer/rec/bio/dbdb/

Virus abbreviations used for non-iridovirus and ascovirus proteins are : AmEPV : Amsacta moorei entomopoxvirus, BmNPV : Bombyx mori nucleopolyhedrosis virus, CoGV : Choristoneura occidentalis granulovirus, EpNPV : Epiphyas postvittana NPV, LdNPV : Lymantria dispar nucleopolyhedrosis virus, XcGV : Xestia c-nigrum Granulovirus. (*) co-locate accession number reference of AmEPV ORF024, 110 & 112 (NP_064892.1, NP064894.1, NP_064806.1).

| **ORF N°** | **Nucleotide positions in the genome** | **Peptide**  **size (amino acids)** | **Molecular Mass** | **Putative Function** | Conserved motifs and domains | **Closest viral or cellular orthologues** |
| --- | --- | --- | --- | --- | --- | --- |
| 1 | 1>2964 | 987 | 114.34 | DNA polymerase B | pfam03104.15: DNA polymerase family B, exonuclease domain. pfam00136.14: DNA polymerase family B, DNA-binding and dNTP binding activities | HvAV3e ORF001, TnAV2c ORF001, SfAV1a ORF001,  CIV037L & MIV120R |
| 2 | 2884<3363 | 159 | 18.03 | - | - | - |
| 3 | 3461>4234 | 257 | 29.17 | RNAseIII | pfam00636.14 : RNase3 domain. smart00358.11: Double-stranded RNA binding motif | SfAV1a ORF022 & 023, HvAV3e ORF027, TnAV2c ORF008, CIV142R & MIV101R |
| 4 | 4304>5236 | 310 | 35.75 | BRO-like protein 1 | pfam02498.14 : BRO family, N-terminal domain. This family includes the N-terminus of baculovirus BRO and ALI motif proteins. | NP_064957.1| AmEPV ORF175, CIV006L, 238R, 313L & 468L |
| 5 | 5274<5760 | 161 | 18.59 | NUDIX hydrolase | cd02883.1 : Nudix hydrolase is a superfamily of enzymes that catalyzes the hydrolysis of NUcleoside DIphosphates linked to other moieties, X. Enzymes belonging to this superfamily require a divalent cation, such as Mg^2+^ or Mn^2+^ for their activity. | CIV414L, MIV111R |
| 6 | 5842>6240 | 132 | 15.01 | - | - | - |
| 7 | 6273>6887 | 204 | 23.22 | IAP-like protein | 2 motifs cd00022.3 are present in the N-terminal region: Baculoviral inhibition of apoptosis protein repeat domain found in inhibitors of apoptosis proteins (IAPs) and other proteins. In higher eukaryotes, BIR domains inhibit apoptosis by acting as direct inhibitors of the caspase family of protease enzymes | YP_654505.1| CoGV ORF3,  NP_001037024.1| IAP-3 BmNPV |
| 8 | 6927>8168 | 413 | 44.48 | Cofactor of the virion S/T kinase encoded by the DpAV1 ORF044 | pfam08793.2: 2-cysteine adaptor domain. The virus-specific 2-cysteine adaptor domain is found fused to OTU/A20-like peptidases and S/T protein kinases. Protein containing SRP-rich tandem repeats of 6-15 residues with a sequence that is virus-specific. | HvAV3e ORF061,  TnAV2c ORF141,  SfAV1a ORF048,  CIV232R, MIV084L |
| 9 | 8213>9004 | 263 | 29.46 | - | - | HvAV3e ORF69, TnAv2c ORF1241, SfAV1a 057,  CIV359L, MIV105R |
| 10 | 9166>9654 | 162 | 18.56 | RNA polymerase II, subunit-like Rpb5b | - | HvAV3e ORF138, TnAv2c ORF051, SfAV1a 089,  CIV 454R, MIV092R |
| 11 | 9721>10185 | 154 | 18.4 | - | - | - |

| 12 | 10186>11232 | 348 | 39.27 | - | pfam00769.14 : Ezrin/radixin/moesin family | Q3IJ53| Translation initiation factor IF-2 [*Pseudoalteromonas haloplanktis* TAC125] |
| --- | --- | --- | --- | --- | --- | --- |
| 13 | 11268<11990 | 240 | 27.45 | - | - | CIV 218R |
| 14 | 12278<12574 | 98 | 11.62 | - | - | - |
| 15 | 12613>13392 | 259 | 30.01 | Fatty acid elongase | pfam01151.14 : GNS1/SUR4 family. Members of this family are involved in long chain fatty acid elongation systems that produce the 26-carbon precursors for ceramide and sphingolipid synthesis. | SfAV1a ORF087, HvAV3e ORF140, TnAV2c, ORF046 |
| 16 | 13415<14371 | 318 | 36.78 | BRO-like protein 2 | pfam04383.8 : KilA-N domain. The amino-terminal module of the D6R/N1R proteins defines a novel, conserved DNA-binding domain (the KilA-N domain) that is found in a wide range of proteins of large bacterial and eukaryotic DNA viruses | AmEPV ORF024, 110 & 112 (NP_064892.1, NP064894.1, NP_064806.1)*, CIV 006L, 238R, 313L & 468L |
| 17 | 14415<15404 | 329 | 37.75 | BRO-like protein 3 | pfam02498.14 : BRO family, N-terminal domain. This family includes the N-terminus of baculovirus BRO and ALI motif proteins. | AmEPV ORF024, 110 & 112*, CIV 006L, 238R, 313L & 468L |
| 18 | 15450<15812 | 120 | 14.03 | - | - | - |
| 19 | 15955>17259 | 434 | 49.92 | Major Capsid Protein | pfam04451.8 : Iridovirus major capsid protein. | HvAV3e ORF056, TnAV2c ORF144 & 153, SfAV1a ORF041, CIV274L, MIV014L |
| 20 | 17284<19851 | 855 | 96.06 | SNF2 DEAD-like helicase | smart00487.11 : DEAD-like helicases superfamily | HvAV3e ORF015, TnAV2c ORF161, SfAV1a ORF009, CIV022L, MIV087L |
| 21 | 19869>20546 | 225 | 25.50 | - | Arginine and Serine-rich protein | - |
| 22 | 20575>20886 | 103 | 12.32 | Yabby-like transcription factor | cd00084.4: High Mobility Group (HMG)-box | HVAv3e ORF130, TnAV2c ORF059, SfAV1a ORF091 CIV401R, MIV068R |
| 23 | 20929>21522 | 197 | 20.30 | - | - | HvAV3e ORF059,  SfAV1a ORFP  TnAV2c ORF148 |
| 24 | 21554>22087 | 177 | 19.91 | - | - | CIV234R |
| 25 | 22147>22962 | 271 | 31.07 | Polymerase processivity factor for DNA replication & repair | cd00577.1 : Proliferating Cell Nuclear Antigen (PCNA) domain found in eukaryotes and archaea. | CIV436R |

| 26 | 22977>24014 | 345 | 39.29 | Metallo-dependent calcineurin-like phosphatase | pfam00149.14 : Calcineurin-like phosphoesterase. | HvAV3e ORF071, TnAV2c ORF121, SfAV1a ORF059, CIV244L, MIV078R |
| --- | --- | --- | --- | --- | --- | --- |
| 27 | 24042<25664 | 540 | 60.02 | - | - | - |
| 28 | 25718>26290 | 190 | 22.50 | Uvr/REP helicase | - | HvAV3e ORF144, SfAV1a ORF086, CIV307L, MIV033L |
| 29 | 26294>26965 | 223 | 24.95 | Zinc-dependent metalloprotease | cd04278.1 : Zinc-dependent metalloprotease, matrix metalloproteinase (MMP) sub-family. | EAT45766| matrix metalloproteinase [*Aedes aegypti*] |
| 30 | 26730<27001 | 89 | 9.82 | - | - | CIV140L |
| 31 | 27333>27980 | 215 | 24.98 | - | - | - |
| 32 | 28023<28985 | 320 | 36.95 | BRO-like protein 4 | pfam02498.14 : BRO family, N-terminal domain. This family includes the N-terminus of baculovirus BRO and ALI motif proteins. | AmEPV ORF024, 110 & 112*, CIV006L, 238R, 313L & 468L |
| 33 | 29026<30918 | 630 | 71.53 | SbcC subunit C | pfam02463.14 : RecF/RecN/SMC N terminal domain. pfam00005.14 : BC transporter. | HvAV3e ORF118, TnAV2c ORF77, SfAV1a ORF103, CIV050L , MIV094L |
| 34 | 31222<32283 | 353 | 40.90 | BRO-like protein 5 | pfam02498.14 : BRO family, N-terminal domain. This family includes the N-terminus of baculovirus BRO and ALI motif proteins. | AmEPV ORF024, 110 & 112*, CIV006L, 238R, 313L & 468L |
| 35 | 32311<33153 | 280 | 31.61 | - | - | HvAV3e ORF066, TnAV2c ORF126, SfAV1a ORF055, CIV254L |
| 36 | 33388>34941 | 517 | 58.82 | Serine/threonine protein kinase | - | HvAV3e ORF117, TnAV2c ORF086, SfAV1a ORF104, CIV098R, MIV038R |
| 37 | 34866>35513 | 215 | 25.26 | - | - | NP_203270.1| EpNPV, NP_148818.1|XcGV ORF30 and ORF31, CIV422L |
| 38 | 35544<35963 | 139 | 15.29 | - | - | - |
| 39 | 35960<36448 | 162 | 18.49 | - | pfam04848.8 : Poxvirus A22 protein. | CIV170L, NP_064944.1| AmEPV ORF162 |

| 40 | 36470<37801 | 443 | 47.24 | - | - | HvAV3e ORF052, TnAV2c ORF157, SfAV1a ORF035, CIV118L & 458R , MIV006R |
| --- | --- | --- | --- | --- | --- | --- |
| 41 | 37816>38130 | 104 | 12.10 | Evrl/Alr thiol oxidase | pfam04777.8 : Erv1 / Alr family. Biogenesis of Fe/S clusters involves a number of essential mitochondrial proteins. | HvAV3e ORF074, TnAV2c ORF118, SfAV1a ORF061, CIV347L, MIV096R |
| 42 | 38140>38520 | 126 | 15.06 | - | - | - |
| 43 | 38553>39638 | 361 | 41.30 | - | - | HvAV3e ORF078, TnAV2c ORF113, SfAV1a ORF65, CIV393L, MIV039R |
| 44 | 39872>40267 | 131 | 18.89 | Thioredoxin | pfam00085.14 : Thioredoxins are small enzymes that participate in redox reactions, via the reversible oxidation of an active centre disulfide bond. | CIV453L & MIV041R |
| 45 | 40273>40620 | 115 | 13.36 | - | - | - |
| 46 | 40642<43065 | 807 | 90.15 | Serine/threonine protein kinase | - | HvAV3e ORF077, TnAV2c ORF115, SfAV1a ORF064 & CIV209R |
| 47 | 43099<43544 | 148 | 16.61 | BRO-like protein 6 | pfam02498.14 : BRO family, N-terminal domain. This family includes the N-terminus of baculovirus BRO and ALI motif proteins. | NP_064957.1|AmEPV ORF175, CIV201R & 289L, MIV019R |
| 48 | 43561<44754 | 397 | 44.05 | Cathepsin B | cd02620.2 : cathepsin B group | HvAV3e ORF103, TnAV2c ORF102, SfAV1a ORF114, CIV224L & 361L, MIV024R |
| 49 | 44744<45238 | 164 | 18.29 | - | AIG2-like family. AIG2 is an Arabidopsis proteins that exhibit RPS2- and avrRpt2-dependent induction early after infection with Pseudomonas syringae pv maculicola strain ES4326 carrying avrRpt2. | AAH80818.1| RIKEN cDNA A030007L17 gene (*Mus musculus*) |
| 50 | 45278>45655 | 125 | 14.27 | BRO-like protein 7 | pfam02498.14 : BRO family, N-terminal domain. This family includes the N-terminus of baculovirus BRO and ALI motif proteins. | AmEPV ORF024, 110 & 112*, CIV006L, 238R, 313L & 468L |
| 51 | 45692<46354 | 220 | 24.82 | - | - | - |

| 52 | 46801<47325 | 174 | 19.86 | ABC-type transport system permease | - | HvAV3e ORF56, 87 & 151, TnAV2c ORF107, 143, 144, 152 & 162, SfAV1a ORF119 |
| --- | --- | --- | --- | --- | --- | --- |
| 53 | 47353<47910 | 185 | 19.93 | - | - | - |
| 54 | 47933<49672 | 580 | 65.53 | Ribonucleotide reductase | cd01679.5 : RNR, class I. Ribonucleotide reductase (RNR) catalyzes the reductive synthesis of deoxyribonucleotides from their corresponding ribonucleotides. It provides the precursors necessary for DNA synthesis. | CIV085L, MIV065R |
| 55 | 49829>50392 | 187 | 22.01 | Thymidine kinase | cd01673.2 : Deoxyribonucleoside kinase (dNK) catalyzes the phosphorylation of deoxyribonucleosides to yield corresponding monophosphates (dNMPs). | HvAV3e ORF55, TnAV2c ORF154, SfAV1a ORF040, CIV143R, MIV029R |
| 56 | 50778>51140 | 120 | 13.82 | metallo-hydrolase | - | YP_441975.1| Gene info metallo-beta-lactamase family protein [*Burkholderia thailandensis* E264], HvAV3e ORF050 SfAV1a ORFQ+R, TnAV2c ORF033 |
| 57 | 51447>51749 | 100 | 11.40 | metallo-hydrolase | - | YP_001067411.1| Gene info metallo-beta-lactamase family protein [*Burkholderia pseudomallei*], HvAV3e ORF50, TnAV2c ORF33 |
| 58 | 52415<52774 | 119 | 13.58 | DNA-directed RNA polymerases I, II, and III subunit RPABC2 | pfam01192.14 : RNA polymerase Rpb6. Rpb6 is an essential subunit in the eukaryotic polymerases Pol I, II and III. | XP_393973.2| RNA polymerase II 18kD subunit CG1163-PA [Apis mellifera] |
| 59 | 53193>53654 | 153 | 17.77 | Acyl-Coenzyme A Binding Protein | cd00435.2 : Acyl CoA binding protein (ACBP) binds thiol esters of long fatty acids and coenzyme A in a one-to-one binding mode with high specificity and affinity. | XP_001356156.1| Gene info GA21340-PA [*Drosophila pseudoobscura*] |
| 60 | 54045>54692 | 215 | 25.27 | - | - | HvAV3e ORF057, TnAV2c ORF150, SfAV1a ORF042, MIV034R |

| 61 | 54735<55253 | 172 | 18.64 | ABC-type transport system permease | - | HvAV3e ORF56, 87 & 151, TnAV2c ORF107, 143, 144, 152 & 162, SfAV1a ORF119 |
| --- | --- | --- | --- | --- | --- | --- |
|  | 55460-55550 |  |  | miRNA ORF-like |  |  |
| 62 | 55254<55502 | 82 | 9.73 | Ubiquitin | cd01803.2 : Ubiquitin (includes Ubq/RPL40e and Ubq/RPS27a fusions as well as homopolymeric multiubiquitin protein chains) | AAF72586.1| GP37 protein [SlNPV] |
| 63 | 55573>56133 | 186 | 21.52 | - | - | HvAV3e ORF053, TnAV2c ORF156, SfAV1a ORF038 |
| 64 | 56178>57362 | 394 | 44.67 | - | - | HvAV3e ORF068, TnAV2c ORF125, SfAV1a ORF058, CIV067R, MIV004R |
| 65 | 57359<58204 | 281 | 31.58 | Myristylated membrane protein-like | - | TnAV2c ORF129, HvAV3e (ORF65bis, frame +1 pos. 73288 to 74247), SfAV1a ORF054, CIV337L, MIV047R |
| 66 | 58252>59439 | 395 | 46.09 | Ribonucleotide reductase | cd01049.2 : Ribonucleotide Reductase, R2/beta subunit (RNRR2) is a member of a broad superfamily of ferritin-like diiron-carboxylate proteins. | CIV376L, MIV048L |
| 67 | 59514>60504 | 329 | 37.73 | BRO-like protein 8 | pfam02498.14 : BRO family, N-terminal domain. This family includes the N-terminus of baculovirus BRO and ALI motif proteins. | AmEPV ORF024, 110 & 112*, CIV006L, 238R, 313L & 468L |
| 68 | 60703>61965 | 420 | 48.54 | NTPase/helicase | - | NP_048295.1| MsEPV ORF MSV224 |
| 69 | 62343<62819 | 158 | 18.29 | - | - | - |
| 70 | 62850<64121 | 423 | 47.63 | DNA-directed RNA polymerase subunit 1 | PRK04309.3 : DNA-directed RNA polymerase subunit A | HvAV3e ORF82, TnAV2c ORF110, SfAV1a ORF067, CIV343L, MIV 090L |
| 71 | 64723>65112 | 129 | 15.26 | - | - | CIV145L |
| 72 | 65139>65501 | 120 | 14.41 | - | - | CIV374R |
| 73 | 65537>68641 | 1034 | 115.23 | DNA-directed RNA polymerase subunit 2 | cd00653.3 : RNA polymerase beta subunit. | HvAV3e ORF064, TnAV2c ORF138, SfAV1a ORF052, CIV428L, MIV009R |
| 74 | 68680<69085 | 134 | 16.14 | - | - | - |

| 75 | 69115<70410 | 431 | 50.14 | BRO-like protein 9 | pfam02498.14 : BRO family, N-terminal domain. This family includes the N-terminus of baculovirus BRO and ALI motif proteins. | NP_064957.1|AmEPV ORF175, CIV201R & 289L, MIV019R |
| --- | --- | --- | --- | --- | --- | --- |
| 76 | 70740>71330 | 196 | 22.59 | - | - | - |
| 77 | 71774>72043 | 89 | 10.07 | Endonuclease | COG2827.2: Predicted endonuclease containing a URI domain [DNA replication, recombination, and repair] | CAJ41444.1| *Serratia liquefaciens* protein |
| 78 | 72165>73112 | 315 | 37.18 | - | - | NP_047746.1| LdNPV ORF109 |
| 79 | 73153<74463 | 436 | 50.60 | - | - | CIV268L, MIV074L |
| 80 | 74463<75575 | 370 | 41.86 | Serine/Threonine protein kinase | cd00180.3 : Serine/Threonine protein kinases, catalytic domain. | XP_629684.1| Gene info putative protein serine/threonine kinase [*Dictyostelium discoideum* AX4] |
| 81 | 75578<76432 | 284 | 32.54 | PlsC phosphate acyltransferase | smart00563.11 : PlscC Phosphate acyltransferases | HvAV3e ORF106, TnAV2c ORF098, SfAV1a ORF112 |
| 82 | 76537>76911 | 124 | 13.95 | Transcription elongation factor-SII | smart00440.11 : C2C2 Zinc finger | HvAV3e ORF099, CIV349L , MIV055L |
| 83 | 77466<78239 | 257 | 28.51 | Hydroxysteroid (17-beta) dehydrogenase | COG0300.2 : DltE Short-chain dehydrogenases of various substrate specificities | NP_957175.1| Hydroxysteroid (17-beta) dehydrogenase 12 [*Danio rerio*] |
| 84 | 78364>78837 | 157 | 17.54 | - | - | - |
| 85 | 78876>82001 | 1041 | 117.91 | Dynein-like beta chain | - | HvAV3e ORF146, TnAV2c ORF043, SfAV1a ORF084, CIV395L, MIV016R |
| 86 | 82016<82906 | 296 | 34.50 | ATPase3 | smart00382.11 : ATPases associated with a variety of cellular activities | HvAV3e ORF109, TnAV2c ORF095, SfAV1a ORF110, CIV075L, MIV088R |
| 87 | 82929<83738 | 269 | 30.36 | Patatin-like phospholipase | pfam01734.14: Patatin-like phospholipase | HvAV3e ORF128, TnAV2c ORF067, SfAV1a ORF093, CIV463L |
| 88 | 83790>84209 | 139 | 15.79 | - | - | - |

| 89 | 84276>86897 | 873 | 98.74 | DNA-directed RNA polymerase subunit alpha | PRK08566 : DNA-directed RNA polymerase subunit alpha | HvAV3e ORF011, TnAV2c ORF042, SfAV1a ORF008, CIV176R, MIV090L |
| --- | --- | --- | --- | --- | --- | --- |
| 90 | 87212>89989 | 925 | 105.35 | DNA primase with a pox D5 ATPase domain | Prim_Pol: Bifunctional DNA primase/polymerase, N-terminal. PriCT_2: Primase C terminal 2 (PriCT-2). COG3378: Phage associated DNA primase -predicted ATPse | YP_001029423.1| Gene info GfV-C20-ORF1 [*Glypta fumiferanae* ichnovirus] |
| 91 | 90251>90736 | 162 | 19.12 | - | - | YP_001029427.1| GfV-D1-ORF1, YP_001029444.1| GfV-D4-ORF1, YP_001029432.1| GfV-D3-ORF2 |
|  | 90700-90850 |  |  | miRNA ORF-like |  |  |
| 92 | 91296>91919 | 207 | 23.94 | ALI-like protein | - | CIV069L, NP_048267.1| MsEPV ORF196, NP_048095.1| MsEPV ORF 024, NP_048096.1| MsEPV ORF026 |
| 93 | 92072>94621 | 849 | 97.31 | Putative ATPase | pfam08707.2 : Primase C terminal 2 (PriCT-2), D5 N terminal like.COG3378 : Predicted ATPase. | HvAV3e ORF119, TnAV2c ORF078, SfAV1a ORF099, CIV184R, MIV121R, YP_001029423.1| GfV-C20-ORF1 |
| 94 | 94677>96914 | 745 | 84.67 | Putative chromosomal replication initiation protein | - | CIV ORF155L, MIV113L |
| 95 | 96959>97450 | 163 | 18.72 | Zinc finger protein | - | CIV132L |
| 96 | 97525>98874 | 449 | 51.19 | E3 ubiquitin ligase | - | AAV91107.1| ligase [Grouper iridovirus] |
| 97 | 98901>99359 | 152 | 17.65 | - | - | HvAV3e ORF111, TnAV2c ORF091, SfAV1a ORF108, CIV373L |
| 98 | 99515>100726 | 403 | 46.33 | BRO-like protein 10 | pfam02498.14 : BRO family, N-terminal domain. This family includes the N-terminus of baculovirus BRO and ALI motif proteins. | AmEPV ORF024, 110 & 112*, CIV006L, 238R, 313L & 468L |
| 99 | 100731<100949 | 72 | 8.32 | - | - | - |
| 100 | 101067<101342 | 91 | 10.86 | - | - | - |
| 101 | 101369<101740 | 123 | 14.28 | - | - | - |
| 102 | 101764<102407 | 213 | 20.06 | - | - | - |
| 103 | 102396<103509 | 370 | 43.18 | Late Transcription Factor VLTF3-like | pfam04947.8 : Poxvirus Late Transcription Factor VLTF3 like. | HvAV3e ORF033, TnAV2c ORF017, SfAV1a ORF029, CIV282R, MIV079L |
| 104 | 103879<103499 | 128 | 14.44 | Putative Thioredoxin | - | XP_764604.1| thioredoxin [*Theileria parva* strain Muguga] |
| 105 | 104077<106494 | 805 | 91.15 | Protein kinase | - | CIV179R, CIV439L, MIV035R |
| 106 | 106577>106819 | 80 | 9.11 | - | - | - |
| 107 | 106849>107268 | 139 | 15.74 | - | - | CIV411L , MIV032R |
| 108 | 107348>107815 | 155 | 18.08 | Zn-finger/nucleic acid binding protein | - | HvAV3e ORF105, TnAV2c ORF100, SfAV1a ORF113, CIV350L, MIV026R |
| 109 | 108205>109080 | 291 | 33.08 | Delta 9 fatty acid desaturase | COG1398.2 : OLE1 - Fatty-acid desaturase. | XP_001357688.1| GA22005-PA [*Drosophila pseudoobscura*] |
| 110 | 109300>110331 | 343 | 39.62 | BRO-like protein 11 | pfam02498.14 : BRO family, N-terminal domain. This family includes the N-terminus of baculovirus BRO and ALI motif proteins. | AmEPV ORF024, 110 & 112*, CIV006L, 238R, 313L & 468L |
| 111 | 110593>111462 | 289 | 30.96 | Oxidoreductase | PRK07985.3 : oxidoreductase. | TnAV2c ORF071 |
| 112 | 111429>112526 | 365 | 42.41 | BRO-like protein 12 | pfam02498.14 : BRO family, N-terminal domain. This family includes the N-terminus of baculovirus BRO and ALI motif proteins. | AmEPV ORF024, 110 & 112*, CIV 006L, 238R, 313L & 468L |
| 113 | 114516<114010 | 164 | 19.94 | Putative DNA ligase | COG0272.2 : NAD-dependent DNA ligase (contains BRCT domain type II) | - |
| 114 | 114060<114515 | 151 | 16.23 | - | - | - |
| 115 | 114533<115576 | 347 | 39.37 | CK1 family protein kinase | cd00180.3 : Serine/Threonine protein kinases, catalytic domain. | NP_586504.1| Gene info CASEINE KINASE 1 [*Encephalitozoon cuniculi* GB-M1] |
| 116 | 115674>116244 | 189 | 20.05 | - | - | HvAV3e ORF129, TnAV2c ORF060, SfAV1a ORF092, CIV259R , MIV071L |

| 117 | 116273<116803 | 176 | 20.54 | CDT-like phosphatases Putative NIF/NLI interacting factor | smart00577.10 : catalytic domain of ctd-like phosphatases | HvAV3e ORF110, TnAV2c ORF093, SfAV1a ORF109, CIV355R, MIV104L |
| --- | --- | --- | --- | --- | --- | --- |
| 118 | 116838>117911 | 357 | 40.39 | Metallo-hydrolase | - | ZP_01639593.1| beta-lactamase-like [*Pseudomonas putida* W619] |
| 119 | 117947<119131 | 394 | 44.14 | RedQ-like DEAD helicase | cd00046.4 : DEAD-like helicases superfamily. cd00079.3 : Helicase superfamily c-terminal domain; associated with DEXDc-, DEAD-, and DEAH-box proteins | EAT44626.1| blooms syndrome DNA helicase [*Aedes aegypti*] |

**Best BLASTP e-value results for the 119 ORFs in the DpAV4a genome using the non-redundant Protein database and the BLOSUM60 comparison matrice**

| ORF N° | BLASTP  e-values | ORF N° | BLASTP  e-values | ORF N° | BLASTP  e-values |
| --- | --- | --- | --- | --- | --- |
| 1 | 4e-170 | 41 | 1e-29 | 81 | 2e-09 |
| 2 | - | 42 | 7e-18 | 82 | 3e-08 |
| 3 | 5e-114 | 43 | 2e-36 | 83 | 2e-15 |
| 4 | 6e-49 | 44 | 1e-19 | 84 | - |
| 5 | 9e-38 | 45 | - | 85 | 1e-57 |
| 6 | - | 46 | 1e-54 | 86 | 3e-79 |
| 7 | 2e-22 | 47 | 3e-08 | 87 | 3e-29 |
| 8 | 1e-06 | 48 | 2e-59 | 88 | - |
| 9 | - | 49 | 9e-22 | 89 | 5e-144 |
| 10 | 1e-06 | 50 | 4e-16 | 90 | 1e-37 |
| 11 | - | 51 | - | 91 | 5e-03 |
| 12 | 1e-04 | 52 | 5e-07 | 92 | 3e-12 |
| 13 | 4e-12 | 53 | - | 93 | 4e-62 |
| 14 | - | 54 | 1e-153 | 94 | 1e-13 |
| 15 | 1e-27 | 55 | 5e-28 | 95 | 3e-07 |
| 16 | 4e-63 | 56 | 3e-07 | 96 | 7e-05 |
| 17 | 6e-46 | 57 | 2e-03 | 97 | - |
| 18 | - | 58 | 3e-11 | 98 | 2e-30 |
| 19 | 9e-67 | 59 | 2e-12 | 99 | - |
| 20 | 4e-92 | 60 | 3e-03 | 100 | - |
| 21 | - | 61 | 5e-03 | 101 | - |
| 22 | 2e-10 | 62 | 5e-13 | 102 | - |
| 23 | - | 63 | - | 103 | 8e-44 |
| 24 | - | 64 | - | 104 | 8e-03 |
| 25 | 4e-25 | 65 | 2e-16 | 105 | 9e-12 |
| 26 | 9e-67 | 66 | 2e-117 | 106 | - |
| 27 | - | 67 | 7e-30 | 107 | - |
| 28 | 3e-33 | 68 | 1e-12 | 108 | 7e-17 |
| 29 | 5e-17 | 69 | - | 109 | 1e-94 |
| 30 | - | 70 | 2e-60 | 110 | 2e-31 |
| 31 | - | 71 | - | 111 | 2e-81 |
| 32 | 7e-32 | 72 | - | 112 | 6e-30 |
| 33 | 4e-21 | 73 | 2e-168 | 113 | - |
| 34 | 5e-30 | 74 | - | 114 | - |
| 35 | - | 75 | 8e-62 | 115 | 8e-14 |
| 36 | 3e-32 | 76 | - | 116 | 1e-11 |
| 37 | 4e-29 | 77 | 2e-16 | 117 | 6e-35 |
| 38 | - | 78 | - | 118 | 2e-26 |
| 39 | 5e-04 | 79 | - | 119 | 9e-105 |
| 40 | - | 80 | 1e-13 | - | - |

**Intrastrand folds calculated with Mfold of the two putative miRNA encoded by the DpAV4a genome**

miRNA1 : Region from position 55460 to 55550

Initial dG = -23.20

10 20 30

C-------- T---- TC- T-| G G

GC AACG TTA CGTTTG AGTTTTTAATAAAAT A

CG TTGC AAT GCAGAT TCAAAAATTATTTTA T

TTTTTACAA TAAAC CAC TT^ G C

. 80 70 60 50 40

miRNA2 : Region from position 90700 to 90850

Initial dG = -60.60

10 20 30 40 50 60 70 80

GTGTTTGTTAATTTTGA| CAGG A ATCT TT TGTC

TCTGAAC TGAACTA CTGAACT TTTTTAGAGCATTTATCAAAAACTTT TGGT G

GGACTTG ACTTGAT GACTTGA AAAAATCTCGTAAATAGTTTTTGAAA ACTA T

G----------------^ ATGA C CCCT -- TGAA

. 140 130 120 110 100 90
