# Supplementary material for: Symbiotic Virus at the Evolutionary Intersection of Three Types of Large DNA Viruses; Iridoviruses, Ascoviruses, and Ichnoviruses
Source: PLoS One. 2009 Jul 28;4(7):e6397. doi: 10.1371/journal.pone.0006397 (PMC2712680; doi:10.1371/journal.pone.0006397)
Supplement: Figure S3 — Relationship between the genome size and the number of ORFs of ascovirus, DpAV4a, vertebrate and invertebrate iridovirus genomes (0.07 MB PDF) [file pone.0006397.s003.pdf]

# S3 : Supporting Information 3

**Symbiotic Virus at the Evolutionary Intersection of Three Types of Large DNA Viruses; Iridoviruses, Ascoviruses, and Ichnoviruses**

Yves Bigot, Sylvaine Renault, Jacques Nicolas, Corinne Moundras, Marie-Véronique Demattei, Sylvie Samain, Dennis K. Bideschi, and Brian A. Federici

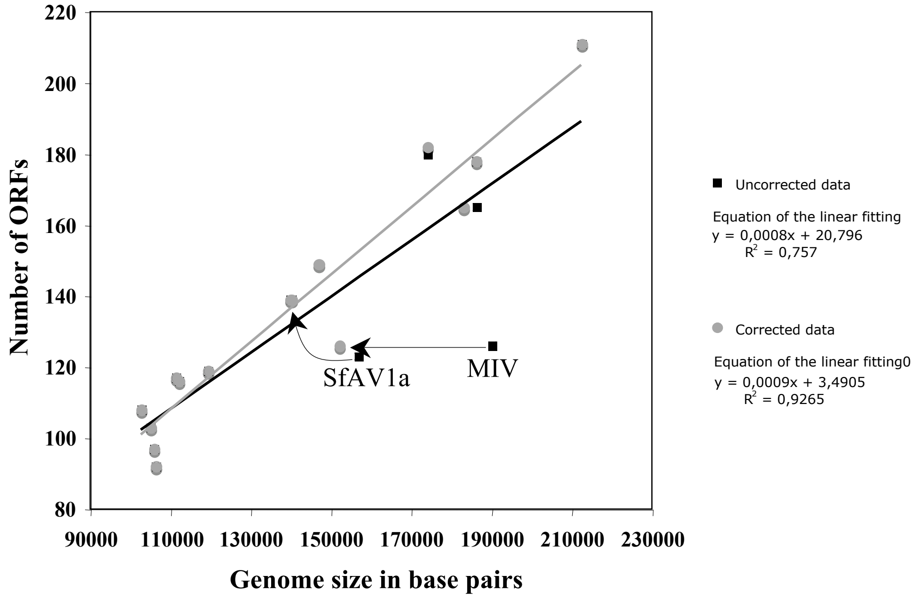


Graphic representation of the relationship between the genome size and the number of ORFs of ascovirus (HvAV3e, TnAV6a and SfAV1a), DpAV4a, vertebrate (FV3, TFV, ATV, GIV, SGIV, LCDV-I, LCDV-china, ISKNV, RBIV) and invertebrate (CIV and MIV) iridovirus genomes. Calculations were done using data deposited in Genebank (black squares and line), and corrected data, taking into account the presence of non-coding repeats and ORF errata (grey dots and line). Arrows identify the effect of corrections made to the genome size and the number of ORFs. Grey dots do not overlap black squares when modifications were done.
