# Supplementary material for: Symbiotic Virus at the Evolutionary Intersection of Three Types of Large DNA Viruses; Iridoviruses, Ascoviruses, and Ichnoviruses
Source: PLoS One. 2009 Jul 28;4(7):e6397. doi: 10.1371/journal.pone.0006397 (PMC2712680; doi:10.1371/journal.pone.0006397)
Supplement: Figure S4 — Complementary information in gene annotation of previously sequenced ascovirus genomesS4a. Sequences of the proteins encoded, in the HvAV3e genome, by ORFs non-referenced in databases, and, having homologues in the other ascovirus genomes S4b. Unreferenced SfAV1a ORFs having homologues in the HvAV3e and TnAV2c genomes S4c. Sequence of the protein encoded, in the SfAV1a genome, by an ORF non-referenced in databases, and, having homologues in the other ascovirus genomes (0.07 MB DOC) [file pone.0006397.s004.doc]

**S4 : Supporting Information 4**

**Symbiotic Virus at the Evolutionary Intersection of Three Types of Large DNA Viruses;**

**Iridoviruses, Ascoviruses, and Ichnoviruses**

Yves Bigot, Sylvaine Renault, Jacques Nicolas, Corinne Moundras, Marie-Véronique Demattei, Sylvie Samain, Dennis K. Bideschi, and Brian A. Federici

**S4a. Sequences of the proteins encoded, in the HvAV3e genome, by ORFs non-referenced in databases, and, having homologues in the other ascovirus genomes**

**S4b. Unreferenced SfAV1a ORFs having homologues in the HvAV3e and TnAV2c genomes**

**S4c. Sequence of the protein encoded, in the SfAV1a genome, by an ORF non-referenced in databases, and, having homologues in the other ascovirus genomes**

**S4a. Sequences of proteins encoded in the HvAV3e genome by ORFs not referenced in databases and having homologues in the other ascovirus genomes**

>HvAV3e-ORF65bis [positions 73288 to 74247 in the HvAV3e genome]

MQIRRKETRSLIIITLTSRSALIKRFLMGSSHSSPTPVIPQVPKKTQTPVNILADIVSLK

YTTSTVPNEADRRLFVPGIDLPQPYIGIDTTKKDGTYAKYDTRLITDPYQLLQISELHAG

DDSGKLRATLEQVFVRLCTATRTSQCHNGASKCMALRSLGGNDSRCQEIAEKLDNNFVDG

LKLKWCLDNTDSTECDCLNRDRSKAYVDLKDFVVTHENLFARDECWYKPCTSDGAMTLST

QKANKCGAKVCINVNAMTAGDKINTGTITDSVQCFNKAHPLRDSSSDWAAGLHDYIEYFN

VALGVLLLIIILYTTYSAK

>HvAV3e-U [positions 80258 to 80518 in the HvAV3e genome]

MDKSLNSNCFKIKKPHLLPLFETLNSSDVEAYELLEHMVTTKCTCVKHGAPDIYCGCSTD

LEQTRQLLLRSSKRSEVSDTTALEGV

>HvAV3e-YY [positions 130405 to130635 in the HvAV3e genome]

MCSKRTLGVYAAMKRLVTDGTVCGTPSCMGDVYKRLYEECIQRMYNMQEQLTNINLELVT

VCAENAAALSGNINYV

S4b. Unreferenced SfAV1a ORFs having homologues in the HvAV3e and TnAV2c genomes

| ORF | HvAV3e homologues | TnAV2c homologues | ORF location in the SfAV1a genome |
| --- | --- | --- | --- |
| A | 002 | 112 | 3359<3877 |
| D | 008 | 023 | 8693>9187 |
| K | 032 | 013 | 34553>34867 |
| L | 037 | - | 36129>36566 |
| N | 050 | - | 41462>41668 |
| O | 049 | 034 | 42152<41524 |
| P | 050 | 033 | 42199>42555 |
| Q | 059 | 148 | Fused ORFs interrupted by stop codons and frame shifts in the SfAV1a genome  [49244<49020] + [49367<49747] |
| R |  |  |  |
| T | 060 | - | 52824<53306 |
| U | Undetected ORF  [pos. 80258-80518] | 120 | 66905<66337 |
| V | 073 | - | 66633<66896 |
| W | 075 | 117 | 67601>68068 |
| X | 076 | - | 68673>68837 |
| Y | 079 | - | 72677>72829 |
| Z | 081 | - | 73943<74323 |
| AA | 085 | - | Fused ORFs interrupted by stop codons and frame shifts in the SfAV1a genome [77176>77246] + [77405>77794] |
| CC |  | - |  |
| PP | 142 | - | 112305<112844 |
| QQ | 141 | - | 112979<113587 |
| SS | 137 | - | Fused ORFs interrupted by stop codons and frame shifts in the SfAV1a genome [116073>116534] + [116744<117076] |
| UU |  |  |  |
| VV | 136 | 054 | 117122>117718 |
| WW | 133 | - | 117785>118192 |
| XX | 132 | 057 | 118203<118862 |
| YY | Undetected ORF  [pos. 130405-130635] | - | 124724<124942 |

**S4c. Sequence of the protein encoded in the SfAV1a genome by an ORF unreferenced in databases, and having homologues in the other ascovirus genomes (**two genes in HvAV3e (ORFs 002 and 112) and TnAV2c (ORFs 024 and 148)**)[[1]](#footnote-2)**

>SfAV1a-ORF1bis [positions 4225 to 3362 in the SfAV1a genome]

MNTTRSDTFRSCIGKCRCVFEYGTIDWSGEAFPHVPRTISSTWLQTSDPRRYRLQLVDELI

DLIEQQRPVNALHSNSITILHAALAEACAVATSKHLSVWVCAELILARVPGTYGIMRTIGG

VRDHHSVAVFSTHPHMRLTSVIDPTEGAYLRNYHEIHKTHSRILNGTIRNDNRRITPLVYA

LRPTTVFNTPTLGRDRELLAATVRHYVDDLRRHKCTIVGTLTDRIVGSCPVDFAQCLDDFI

TLMHVQMNRLSAQRFDEMRPTSCADRMWTSRNIPRKPWWIESCI

1. Bigot Y, Samain S, Augé-Gouillou C, Federici BA (2008) Molecular Evidence for the Evolution of Ichnoviruses from Ascoviruses by Symbiogenesis. *BMC Evol Biol* 8, 253. Dol:10.1186/1471-2148-8-253, pp 1-12. [↑](#footnote-ref-2)
