# Supplementary material for: Symbiotic Virus at the Evolutionary Intersection of Three Types of Large DNA Viruses; Iridoviruses, Ascoviruses, and Ichnoviruses
Source: PLoS One. 2009 Jul 28;4(7):e6397. doi: 10.1371/journal.pone.0006397 (PMC2712680; doi:10.1371/journal.pone.0006397)
Supplement: Figure S5 — SfAV1a virion proteins identified by mass spectrometry (0.11 MB DOC) [file pone.0006397.s005.doc]

**S5 : Supporting Information 5**

**Symbiotic Virus at the Evolutionary Intersection of Three Types of Large DNA Viruses;**

**Iridoviruses, Ascoviruses, and Ichnoviruses**

Yves Bigot, Sylvaine Renault, Jacques Nicolas, Corinne Moundras, Marie-Véronique Demattei, Sylvie Samain, Dennis K. Bideschi, and Brian A. Federici

**S5. SfAV1a virion proteins (21) identified by mass spectrometry[[1]](#footnote-2)**

| **DpAV4a ORF** | **SfAV1 ORF** | **Assigned function^#^** | **Other closest relatives** |
| --- | --- | --- | --- |
| 085 | 084 | Dynein-like b chain | H:146  T:043  C: 395L |
| 020 | 009 | SNF2 DEAD-like helicase | H: 015  T: 161  C: 022L |
| - | 047 | DNA puffC4B-like protein | H:060 |
| 046 | 064 | Serine/threonine protein kinase | H:077  T:115  C:209R |
| - | 027 | Unknown | H:030  T:011 |
| 008 | 048 | Neurofilament triplet H1-like protein  Structural proteins bound DNA[[2]](#footnote-3) | H:061  T:141  C:232R |
| - | 015 | Inhibitor of apoptosis | H:022  T:006  Se:NP_0378844 |
| 019 | 041 | Major capsid protein | H:056  T:153  C:274L |
| 065 | 054 | Myristylated membrane protein-like | H:ORF64bis^*^  T:129  C:337L |
| - | 075 | S1/P1 nuclease | H:134  T:135 |
| - | 033 | Unknown | H:046  T:018 |
| - | 043 | Unknown | H:052  T:149 |
| 040 | 035 | Unknown | H:052  T:157  C:118 & 458 |
| - | 003 | Unknown | H:004 |
| 041 | 061 | Evrl/Alr thiol oxidase | H:074  T:118  C:347L |
| 117 | 109 | CTD phosphatase | H:109  T:093  C:355R |
| 063 | 038 | Unknown | H:053  T:156 |
| - | 036 | Unknwon | H:052  T:038 |
| 022 | 091 | HMG_Box/Yabby-like protein | H:130  T:059  C:401R |
| - | 002 | Unknown | H:003 |
| - | 060 | Unknown | H:072 |

*^#^* , Function assignment[[3]](#footnote-4). H: HvAV3e, T: TnAV5a, C: Chilo iridescent virus type 6, Se: *Spodoptera exigua* nucleopolyhedrovirus. ^*^, ORF64bis located from position 73288 to 74247 in the HvAV3e genome. In blue, was located an ORF present in all ascoviruses so far sequenced that was absent in the available genome sequences of the two invertebrate iridoviruses, CIV and MIV.

1. Tan Y, Bideshi DK, Johnson JJ, Bigot Y, Federici BA (2008) Identification of twenty-one structural protein components of the *Spodoptera frigiperda* ascovirus 1a virion by proteomic analysis. *J Gen Virol* 90: 359-365. [↑](#footnote-ref-2)
2. Tan Y, et al. (2008) P64, a novel major virion structural protein involved in condensing and packaging the *Spodoptera frigiperda ascovirus* 1a genome. *J Virol* 83: 2708-2714. [↑](#footnote-ref-3)
3. Bideshi DK, *et al.* (2006) Genomic sequence of *Spodoptera frugiperda Ascovirus 1a*, an enveloped, double-stranded DNA insect virus that manipulates apoptosis for viral reproduction. *J Virol* 80: 11791-11805. [↑](#footnote-ref-4)
