# Supplementary material for: Symbiotic Virus at the Evolutionary Intersection of Three Types of Large DNA Viruses; Iridoviruses, Ascoviruses, and Ichnoviruses
Source: PLoS One. 2009 Jul 28;4(7):e6397. doi: 10.1371/journal.pone.0006397 (PMC2712680; doi:10.1371/journal.pone.0006397)
Supplement: Figure S6 — ORF homologues between Ascoviruses and Iridoviruses (0.20 MB DOC) [file pone.0006397.s006.doc]

**S6 : Supporting Information 6**

**Symbiotic Virus at the Evolutionary Intersection of Three Types of Large DNA Viruses;**

**Iridoviruses, Ascoviruses, and Ichnoviruses**

Yves Bigot, Sylvaine Renault, Jacques Nicolas, Corinne Moundras, Marie-Véronique Demattei, Sylvie Samain, Dennis K. Bideschi, and Brian A. Federici

**S6. ORF homologues between Ascoviruses and Iridoviruses**.

ORF homologues shared by the three virus families are highlighted in light yellow. Those highlighted in light blue are restricted to the ascoviruses (HvAv3e, SfAV1a, and TnAV6a), DpAv4a and invertebrate iridoviruses (CIV and MIV); in light green to the ascoviruses and DpAV4a; in purple to the ascoviruses; and in light orange to the ascoviruses and invertebrate iridoviruses. This table was developed using data published previously[[1]](#footnote-2).

1. Asgari S, Davis J, Wood D, Wilson P, McGrath A (2007) Sequence and organization of the *Heliothis virescens ascovirus* genome. *J Gen Virol* 88:1120-1132. [↑](#footnote-ref-2)
