# Supplementary material for: Symbiotic Virus at the Evolutionary Intersection of Three Types of Large DNA Viruses; Iridoviruses, Ascoviruses, and Ichnoviruses
Source: PLoS One. 2009 Jul 28;4(7):e6397. doi: 10.1371/journal.pone.0006397 (PMC2712680; doi:10.1371/journal.pone.0006397)
Supplement: Figure S7 — Conserved nucleotide motif in upstream and dowstream gene regions. S7a: A comparative summary of the transcription features of the 28 core genes shared by ascoviruses, DpAV4a and invertebrate iridoviruses, sequence analyses of DpAV4a, and data published for CIV. S7b: Conserved nt motifs in the 5′ UTR of the 28 core genes shared by ascoviruses, DpAV4a and invertebrate iridoviruses. S7c: Conserved nt motifs in the 3′ UTR of the 28 core genes shared by ascoviruses, DpAV4a and invertebrate iridoviruses. (1.11 MB DOC) [file pone.0006397.s007.doc]

**S7 : Supporting Information 7**

**Symbiotic Virus at the Evolutionary Intersection of Three Types of Large DNA Viruses;**

**Iridoviruses, Ascoviruses, and Ichnoviruses**

Yves Bigot, Sylvaine Renault, Jacques Nicolas, Corinne Moundras, Marie-Véronique Demattei, Sylvie Samain, Dennis K. Bideshi,

and Brian A. Federici

**S7a: A comparative summary of the transcription features of the 28 core genes shared by ascoviruses, DpAV4a and invertebrate iridoviruses, sequence analyses of DpAV4a, and data published for CIV.**

**S7b: Conservation motif analysis within the sequence of the 150-bp located at the 5’extremity of the 28 core genes shared by ascoviruses, DpAV4a and invertebrate iridoviruses.**

**S7c: Conservation motif analysis within the sequence of the 150-bp located at the 3’extremity of the 28 core genes shared by ascoviruses, DpAV4a and invertebrate iridoviruses.**

**S7a: Summarized comparison of the transcription features of the 28 core genes shared by ascoviruses, DpAV4a and invertebrate iridoviruses from sequences analyses and literature for DpAV4a^1^** and literature for CIV.

| **DpAV4aa ORF N°** | **Transcription category[[1]](#footnote-2)** | **N° of the CIV ORF homologues** | **Transcription category in CIV[[2]](#footnote-3)** |
| --- | --- | --- | --- |
| 1 | Early | 037L | Delayed-early |
| 3 | Early | 142R | Delayed-early and late |
| 8 | Early | 232R | Early and delayed-early |
| 9 | Early | 359L | Early and Late |
| 10 | Early | 454R | Delayed-early and late |
| 19 | Late | 274L | Late |
| 20 | Early | 022L | Delayed-early |
| 22 | Early | 401R | Delayed-early |
| 26 | - | 244L | Early and Late |
| 33 | - | 050L | Late |
| 36 | Early | 098R | Early, delayed-early and late |
| 40 | - | 118L & 458R | Early and Late/Early and delayed-early |
| 41 | Late | 347L | Early and Late |
| 43 | Early | 393L | Early |
| 48 | - | 224L & 361L | Late/Early and Late |
| 55 | Early | 143L | Early and late |
| 64 | - | 067R | Delayed-early and late |
| 65 | - | 337L | Early and late |
| 70 | Early | 343L | Early and late |
| 73 | Early | 428L | Early and late |
| 85 | - | 295L | Late |
| 86 | - | 075L | Delayed-early and late |
| 89 | Early | 176R | Early and late |
| 93 | Early | 184R | Early and late |
| 103 | - | 282R | Late |
| 108 | - | 350L | Early and late |
| 116 | - | 259R | Early, delayed-early and late |
| 117 | - | 355R | Early and late |

**S7b: Conservation motif analysis within the sequence of the 150-bp located at the 5’extremity of the 28 core genes shared by ascoviruses, DpAV4a and invertebrate iridoviruses.**

**Method of analysis**: Repeated motifs in the 150-bp upstream the start codon of DpAV4a, SfAV1a, HvAV3e, TnAV6a, CIV and MIV genes were located using the MEME software at <http://meme.sdsc.edu/meme/intro.html>. In the sequence alignments, methionine start codons are in white and highlighted in dark grey. TATAA boxes are underlined. The conserved and few degenerate early promoter motifs AAAATTGA are highlighted in yellow^1,2^,[[3]](#footnote-4). Despite whether this motif was more degenerate in these viruses than in mimivirus, it was found in 50% of the iridovirus, ascovirus and bibivirus gene promoters, as previously described for mimiviruses and phycodnaviruses^1^. The other conserved motifs in 100% of the sequences are highlighted in grey and blue. They were similarly coloured despite the fact they have no obvious sequence similarity to each other. The conserved motif of the late gene promoter sustaining MCP expression^2^ from the DpAV4a ORF19 is highlighted in magenta. The conserved motifs located in 100% of the promoter regions of each of the 13 other non-early promoter genes are highlighted in black or in dark blue and typed in white, even though they have no sequence similarities to each other or with the ORF19 promoter. In certain sequences of these 13 genes, early promoter motifs (AAAATTGA) are located and highlighted in yellow. In contrast to what was previously proposed^1^, we were unable to confirm that conserved ATTGATCTT motifs are present in the putative late promoters of ascoviruses, invertebrate iridoviruses and bibivirus. At the end of the 28 sequence alignments is a table describing the length of each intergenic and untranslated sequence of region and the orientations of the gene upstream (1) and the core gene (2) in each genome. For each virus, when the gene upstream and the core gene are not in the same orientation, the cases are coloured.

**Upstream region of DpAV4a ORF001**

>DpAV4a ATTTAGCCAGAGCGTTAGTGGCGCGGTCCATTTTGTCGCGTACGATCGCAACCTGTTTGATAACGTCTTTCAAGTATTTATCGTCGCGGGGCTTGCTCAT

>SfAV1a gtgtcgccatacgtagttgttctcgcggtctcagattccacaattctgccattgttcgaacgcacgcatcgtatccattgtttacacatgcacatcggtc

>HvAV3e cgtatcgccaggcatactttcaatgtagtaatagacttgaatacagacacatcaacttcttgaaatcaatttttcaattattaaaattgattgcaactaa

>TnAV6a gatgaccttgagaaattagaaagttacgttgatgatatcgtcaaagagaaaaattgattttaccgagacacaaccactattggacgtatagaccaagtaa

>CIV acctattagaacatcaattagaaaataaaagggagttaaatagacaattgggaatattttaaaaccaaagattgtggttttaaaatacagccataaaaaa

>MIV gatggacacgagagcaaggctcgaaccaaaaggtacgtgtcaatcttaatttggaattgtgtaagttatgttgaatctgaacctttaaatgaatccaagg

>DpAV4a TTATAAATAAATGATTTGTTGGTTTAAGGCGAAACGATAAAACAATAAACATG

>SfAV1a aattttcttaaaattgattcactaccataatccgaacgctggaatagttaATG

>HvAV3e cgtactcacaactcttataaaatcgtagtacactacattacaaagttaacATG

>TnAV6a cgaagaaggaagatgatgagttattgacgtaatattattaattaaaaaaaATG

>CIV ttgattatttgttttcgaagagatttaaaaaattaaatgtacattataaaatg

>MIV ggttaaaaattgaatttttggggtggaaaaattatattataaagaatataATG

**Upstream region of DpAV4a ORF003**

>DpAV4a CGAATAGAAGCGGCACGGACGCCAACACCGTAACCGCTGCGAGCATCCCGCATATGATGTATAAATTAGTTCTTTGTCTCATTTTATGCTTGGCGCCCCG

>HvAV3e27 gtctacacactcattttttctattaaccaaattaacaatcccagtcttgcagatttacacgcgtcaattcatttttttgccaaaaacaagaaaacaaata

>SfAV1a22 tattatgtagtagttttttttgcaataaatttacaatacgaatgagttgtttcaattcccccttccacatatcatcatcagtatatgggaacatcccgtg

>SfAV1a23 tacggtcatttcgtccgccaccgccggcaggtcgcgggacgaatatatcgcgccagtagccgagttatgaccatcataccacaactggcggaatacacac

>TnAV6a attttagttagttttataataatgaatgttagttagtttaaaattatggtgtttagttactactattacttttatgtgtattactaatttattaattaaa

>CIV ctaaatcttttttgttttaaaactcattgagttttaaaacctttctttaagtttaaatgtaggaggtatatacctacacttgtatttaaaacgttttaaa

>MIV aagtcgcactttttgtcataatgacgccatgttggacccttgtacttgatgggtcgttttgaaatgggatgaaagtatggatcgttgacccacaccaaac

>DpAV4a GCTTCAACTCTAAAAATTGTTTACATAATTCGGTCCCATATGTAAGATAAATG

>HvAV3e27 caatatctcaccgatatccttgcgtatctgaatgatacttacttttaaatatg

>SfAV1a22 tacacatgacacgtgtttgtgtgtattaattaaatcacatatcgttcagtatg

>SfAV1a23 cgaagagttcaatatcatcaccgcaacaacaacacgacgacgataacgacatg

>TnAV6a taatctatgacaaatgtattcttgtatttttaatttatttcatcattaaaatg

>CIV acttttaagaaaaatgaaacattaatataagacttgtaatttaaataaagatg

>MIV aatcgtgcacatcgttcattgttttattaactcggttttaaaacaaaggtatg

**Upstream region of DpAV4a ORF008**

>DpAV4a CTGCCGTGCGGCCACGTGGCTACATGTCTTTCTTGTTCTATCAAACAGATCGATCGAAAGTGTCCGCTGTGCTCCTCTCAATACGTGTCAAAGTTCCGGG

>HvAV3e61 gtctggacacacggacaacgatgactgggactattatgacattgtcgatgagacagttgatgtcatccaagaaccaatgctgaggcgtacacgtaggcgc

>SfAV1a cgaaaataacacggacaacgacgactgggactattacgatatcgtcgacgagtctgacgatcagatcgttcaaccgatgctgcgtaagcccaggcggcgc

>TnAV6a Agtttcaagaaacctaaaggtatacgtgataaaaaacccaagtaggtcgtaaaaatacgtatgtagatttatcattattattaacaatacttctttaaaa

>CIV_232 aggaagttcttttgtatctaaatccattttatttttagaatttttattcatacatattaaagtaactcaaatatcaagtgaaatagattcataccgttac

>MIV_084 gaagcaatggttgagctttaaacccctgttcggcaaaccgggggcaattggagcaatgctgaacggattttgaaaacttatggatcatttcatcttttac

>DpAV4a TCTATTTCTAAAGTTGAACTTTTAATCCAAGTATAATATAAACACTAACAATG

>HvAV3e gcactgtctccaaaataatctcaaccgttactagtaaagcattgataataatg

>SfAV1a gtgaactctccaaaataatctcaaccgttactagtaaagcatttatcctgatg

>TnAV6a Cccacgtggaaccatgaaataattctgactactataagtaaagcaaagaaatg

>CIV_232 aaaatgAAATTGAagttccaaatgtaaataaaaattattaaataaagatcatg

>MIV_084 aactggaaattgaaaattcacccatcatttgtcattaaataaagaccaatatg

**Upstream region of DpAV4a ORF9**

>DpAV4a CGTCGCATCGATGGTGGAAGCGTTGGAGAGTGTGAGGAACAAGCCGCGCATCTACACGCTACTCCAATCGGAGCAAGCTCTATTGAAGACGATCGGCCTG

>HvAV3e69 acacggtcgtgtttgtgtttggtaaatttttaggattaagtggtatatttagcatgttggcagacatcggaaataccaacaaaacgaacccacccggcac

>SfAV1a cgtgaaaatcatcaacgaaaagagtgcgaaccgtactaccaaaccctcacataaagctatcaagcgaacctcgattcgttgagtgaactcctttaacaca

>TnAV6a atggtgacggtgattgcttttgtgtactatcaaaaatataagtaactcactcattttattgattatactgttacctcgaaataacgctcgagttgaactc

>CIV_359 tatctatgctttaattattatttttatattaattattttctttgtactgcgtaaataaatttttaatgacaaaagtcattaagaattactgttttaacta

>MIV_105 Xttgcccctttcaaacaccacacaaccaacattgtgcaccgaaaacaattgaaatgctttatggtgggtggggacttgaacggtgagaacgttgacccca

>DpAV4a ATCTAAAACAATTGAACCAATTTTTATATTTGATATCGTCAAATATAAATATG

>HvAV3e69 cgtcggtagagaagaagacgacgataacgacttagacgatgacggatacgatg

>SfAV1a acgctcgagtgacactcgagcgttcagtaccaatccacacgttatccgagatg

>TnAV6a gagcgttattagtgttattcttgtttcgtttaagagtgaacgtcgtcaatatg

>CIV_359 attaaaataAAATTGAacctgtgtaaataaagtcgttttaaaaaagaaaaatg

>MIV_105 Gagagttggaaagtgaaaaattataaaaaaaatataaataaactagtaaaatg

**Upstream region of DpAV4a ORF010**

>DpAV4a GGAAGAAATTTGTTTATCAGCCTGTTTACAGAAAAAAACGTCAGTTCCTTGCACACTCCGAACGCAAGACTCGTACACAACATCATGAGATTTCTCTTT

>HvAV3e138 tccgctcacagttacgagctcgtcgacgtcttctgctcctagacattgaacaagacgaatacgattctgcatattcagataactacgaagaagaagaca

>SfAV1a aaacgccaacgatggattctagaaaatcgagggaggcgggagtcaaggagtcaacgttgaatcgttccattgtcgtggttgtacttgaaaacgatacat

>TnAV6a gctgataataaaaattgtacatttagtcagtgttaccttcaacgatttgaaggctcagaaagcgatggaattaagcgggaagacataatgttgaagtag

>CIV_454 gtaaataatatactggtttttcaaaatatttttgttgatacatttattttaagaaatttgttattaagttactaactcttacacatcttaaagttaaaa

>MIV_092 gccggtaatggatcctccacccagctgacccgacatcaattcggacaatggatcgtaaaatttaaaattggacatgtttatttcctgcccaatatggag

>DpAV4a TATAAAATGAGAAATTGAAAACGTCCGCGACCCTTTCCGTTAAAATAAAAATG

>HvAV3e138 acgaggacgataaataacggtatcaagagaaaaatcattgttttactaacatg

>SfAV1a atcggcagcgatagggtactgtcaatcaatttccaatgatcgagtagtaaatg

>TnAV6a ttgtggccgtgttaccattcgaagaagaaataccgaaagttcggtcagaaatg

>CIV_454 ttgaataataattttatgaattatgatgattattagattaaaaaattaaaatg

>MIV_092 ggaaattgaatttttgttaatggtaaaattaactagaataaacccaaacaatg

**Upstream region of DpAV4a ORF019**

>DpAV4a TAATCCATACTTTATATATAAACCCCATTTTGATGAATGTGATAATAGTAAGGCTTTTGGCAAAATTGTTTTATTGCTCGCAGCTTTTTGTGGTATTTTC

>HvAV3e56 cgtcaaagtgtttggcgaccgatcgcatgacgctgctcttaccgctacctatgttaccctcgaccgacacgtacacgacggactcggtttctgtcgtcga

>SfAV1a cgcaaaagtacaccagtccatcgtacctctcggcggctttacgcatcacgctgctcttaccgctgccaatgttcccctcgaccgagacgaatactaccga

>TnAV6a acttttccatcgtattcttcaaccaatttgtttattatcgtagattttcccgaaccaatgttaccttcaatagatatgattatcatgatgatgttgggta

>CIV_274 taaactacgcagaactctcgcgatcgtaactattctctaatttttttacttgcatgaaaaataagaagacatgaaaatttaaaagcatttaaaaatagta

>MIV_014 ggggagacattttgcaatttttatccataccgccaaagcttgggttcgagtagtatttcataggttattttttaactataaaaaattaccaactgtttcg

>DpAV4a CTCGATGTACATTAGTGTTTTGGTGATTTTTTATATTCTGTCTGTAATAAATG

>HvAV3e cgacatcaccactatttgatcttcacgtagtaaataattaaagcgttacaatg

>SfAV1a gtcggtcgtcatatttgatcttcaggtagtaaataaataaatcgttagatatg

>TnAV6a ggttttaaaagttcaattgatccaatccaagtaaaagtaaagaatacacaatg

>CIV_274 atctgattgtattcaaatttaaatcttggcaatgtaataaataaacaaagatg

>MIV_014 agggttttgtaaatttttcagccgaaatattgcaactaataaatctaaacatg

**Upstream region of DpAV4a ORF020**

>DpAV4a AAGCAGATCGACGCCTCGGAGACGGCGAACGTCTTCTGGTGGTCGACGCCATGGCCGCTCTGCCGGATCTGGCAGTCCACTCTGTGCCGAGACCCGCACT

>HvAV3e caaaatgtaactattttttctgtaaaaagtgtaagctagctttgtacgatcattatacacccgatgaatgtattgtatgtgaataaaacactattttaag

>SfAV1a tgtacgagccaccagtagtgagatccgttatcgagtattgtgattatttcttttgctcgacttgtcgtttctgcttgtatgatcactacacatacgatgA

>TnAV6a gtaacatatggtcgcgtaagttcacgaccgtttcaaggataagaaatacaaacaatgaggtaataatgtctgcacctcctcatcttcactaaaaataata

>CIV gactgcagatcttgaatattataacctatgtttaaaagaagttatgatgaattttcatcctacctttttaagtaggatgaatttggatgtttgtgattca

>MIV gattttcgaccctccctggttcgggtccataaaattggccgggttggggttgatcatgtccagatcgaggggtttaattttaatcgtcgtttcgtcctgt

>DpAV4a ATTTCTTGGGGCACGTTTTCTTTTAGTATCCATGTTTATATTATATAAACATG

>HvAV3e agctgtttcatttgaattttaattgtagttgcacgatcaactagtgtaaaatg

>SfAV1a atgtactctttgtcagacatgatttcttttttacgcacacaataaaatacatg

>TnAV6a ccatcactcactattgttgttttacaaataattttcattgggtaaaaagcatg

>CIV aacttatttgtattaaaataagtttgaagaataaaatcttaatagataaaATG

>MIV agttcaatcatcctttattattgaaaaaaaccacattttggtttaaataaatg

**Upstream region of DpAV4a ORF022**

>DpAV4a CCAGGCGCCGTCGCAGGTCGCGCAAGTCTCGCGCATCCTCCACCGACGAGTTTATGGCCCGCGACCACAAATTTTCATTGTTCGACAGTCCTAAAAACAT

>HvAV3e130 tagtcttttgatacataatattaggaactagtataggaatacgatcataattaactagacaatatacgaccttattatcgcattgacattttttactatt

>SfAV1a caagaatttattatgtaactacataaatacatatacatttggttacgaaaaaaaaaaaaaaaaaactacaacaataacaaaataggaactaaggacatga

>TnAV6a cataatctttggtgctgagggtgtatacattatacttaacttttacttagataacatacgggaggaaaagaatgaaaataaaagaacacaattcaaacgt

>CIV atataaatgaaacttcttaattttattttaattatttttaatgcattgaagtcaagacatctgatcagatgtcttgactctaaatgccataaattatttc

>MIV atttgtgtccgtgtttggcgcaacctttctccaccgactggaaatttggttggtttaagctctcaccaagtctacatttgaaaaattccatcattagggt

>DpAV4a ACAAGAATTTTTAAGCAGTTGAGTTAAAACAGAGCCGACAGTAAATAAAGATG

>HvAV3e tctaatacagaatttttaaattttcgcatctattacagtaaaagagtatcatg

>SfAV1a atagtgaataaattcaaaaaatcgcatctcacactagttaaacaattacaatg

>TnAV6a acaattttagtttaataaataataatttttttatcttatgcctatagtaaatg

>CIV ctcttcttgaagtaaataagtccactacctcattgtaaacggcagctaaaATG

>MIV tattccatctttaggaaaaaagaaacgcattaataaacaatctgtgcaccatg

**Upstream region of DpAV4a ORF026**

>DpAV4a CGAACGATGCGACATATTCATGGAGACGGACACCATGAACGTGGTTTTCGAAACCAACATCAAAAATTACAGAGAAAGTAAGACCAAAATATTCGGAAGG

>HvAV3e071 atatgttcgagtaactcgtatgcctctacgtcggacgagtttaaagtctcaaaaagtggaagcaagtgaggtttctttattttaaaacaattcgagttca

>SfAV1a agcaacgcgtacgcatccggcctgtcgctcggaatacgttcgaacaacggcagcaagtgcggcttttttatcgtaaagtttcgcctggacaataacgtct

>TnAV6a ccggtctgtcgtcggggattcggttaaagagacacaataaatgtggcttagtaatcgagaattcactacactttttggtttcgaaacaacaagtactgtc

>CIV atattcataatattaatacaacaaaagcagaatgtaaatttgatctaatcgaatcattccttcaacaacaaaaactttatttctatgaaaatttagatga

>MIV tgttttggatcagacactcgagggtgccgtgggactgggacattgaggcggacgagcccgatgtagaaatctactacgattagggtgggctggcaacact

>DpAV4a ATCAGAATCGGAATCACGAACAACGTTAAAGCCTAAGCGCAGCAATATAAATG

>HvAV3e71 ggctcttgtccatatcgaggagtagtggtggttttttcgactttttaaaaatg

>SfAV1a tcgacatcgttatccctgtacgtttatatagtgttaaaaataccttcaagatg

>TnAV6a aataatcatatttattattgatagcgaaacgcgaaattaatgatataaatatg

>CIV agaatagaaatattttaatttaaagcttattttttattaaccaaataagaatg

>MIV taaaattgaactaatttcatttgttcgaggaggagagaataaacctgagcatg

**Upstream region of DpAV4a ORF033**

>DpAV4a GCTGCGCATGCGGTTCGACGACATGATGCTCAAACTCCATGAAGTTTCCAACAAACTTAAAGAAGTGTCGGAAAGCCTGGATAACGCCGACTACTAATTC

>HvAV3e attcattgtgtgagtgtcaacttatacggtgttgctcgctaatttatgcgcgaattatgtttaccaatcaattccgataagagctggatgtaataagacg

>SfAV1a tgaggtatcgtcaggcgttgtcaccgagcactaactacacaaacacacaaccttctcgtaatcaattccgataatggtgacgtttgacaccaataagata

>TnAV6a aaaaatagtgacacgcattatagatactacttatttgtattttatgcgacaccaagacttgatttaaattcaagtgtaaacttcggttttgaacgacatc

>CIV aacagtggtactagacgtaattccaaaggaataagaattttttttatcaattttttgttccattattgaaaattctttatttgcacaacaattaaaaaat

>MIV gcgggaccttgacgcggaggaagaggacagcgacgaggttcgataggaccgcggtcgaccattattatttctttacccaacacaccaggttggttaaagg

>DpAV4a TCTCACTCGAATTTCGAGTGAGAGTTAAAGGCAAAAATTGGTAAATAAAAATG

>HvAV3e agtcgtctatcgttcgaatcgctcagtacgttagtatcagcggcagcgaaatg

>SfAV1a ccccgttcggagcgtaacctcgccagtgatcagatgcgagccgactcgatatg

>TnAV6a agtgtcgacgctcttcggctcaggacattatattaatatcgacgataaatatg

>CIV aaagagaagttttttgttttaaagataagatttaatggattaaactcataatg

>MIV aaaaaaggtaaaatgatatctatcggtttgggaatttaaataaaataaacatg

**Upstream region of DpAV4a ORF036**

>DpAV4a TGCATTCACGTTCCTGGCGTCGCTGATCCGTTCCAATGTTTCTAATGCCCAAATAATATTGGTGCTCTTCTGCGTGTTCGCGTACGTCATCGTCTTCCGC

>HvAV3e117 cttcatttcgctgccgctgatactaacgtactgagcgattcgaacgatagacgactcgtcttattacatccagctcttatcggaattgattggtaaacat

>SfAV1a tcggctcgcatctgatcactggcgaggttacgctccgaacggggtatcttattggtgtcaaacgtcaccattatcggaattgattacgagaaggttgtgt

>TnAV6a tcaaccggctgacttgttcggttacatttatccgtcattggcaaaagaagaagtagaaaaaaaattgatattaattaaagaaatcgaagatcaatccaat

>CIV_098 acgactgagccccgagacgacgaaaatctatataaaccatctattgattctggtaagcttgaagtcatttattaaaagttatatatttattgtatatttt

>MIV_038 tggtgtgttcatcgcaggctgttgtgaaagggtttgggcgtttagttgtatcccactcgttgcagcattcatttattaaagcatccattatcccagtaaa

>DpAV4a GCCAACTACGAAGACGATCCGCAAATTAAGGTGAAGCAAGCTTAAATAAAATG

>HvAV3e117 aattcgcgcataaattagcgagcaacaccgtataagttgacactcacacaatg

>SfAV1a gtttgtgtagttagtgctcggtgacaacgcctgacgatacctcagcaacaatg

>TnAV6a aataatacggtttcagatacataaagttgtacattgagaagaccacaaaaatg

>CIV_098 attttggatatttatttaaaatacttaaatagtgatcatagattattaaaatg

>MIV_038 tggcaagtaaattgaattttttaccccattaatagggtataaaataaaacatg

**Upstream region of DpAV4a ORF040**

>DpAV4a GCAGTATCTTGATGAACTGAGACGCCACCTCGACGTCTACGACGTTGGGTTGTTCCGGATACGTATCGGAATAAGTGTGTATCACGTACCAAAAATGCGG

>HvAV3e052 ctgaatgagtgtccgacgtgtcggagtaacattattgtgaccgtcaatcgttgtcaatagtaataattataataaatatttacagcgatctatttggtta

>SfAV1a gtatgcgagcgtacgtgaacatattcgaccgagccagcagcacgctataccacttggacggtaaaatcgaagcaatcgacgtgtcctacggacaacaacg

>TnAV6a ttcttggtacgacagctgtttttcagagttttaatgtagcctctttagctcagtggttagagcatcggtcttatacgccggttgtcgtgagttcgattct

>CIV_118 ctggggcatattacggaggttgtttttaattttttatattttttatggaatttttatggcattaatgccataaaaattaattgtctaatttttctttatt

>CIV_458 ccaggggctccaattggggcaaagcctccgcgtttcacgggagggaaaccgccgacgcattaatcttattaatgtttttttttatttttggttttataaa

>MIV gaggcacatttgcgccgtgattgaagggagtcggtttttggtgtgattgaaccgcaaaactccctcattggttgcctatggttgggtggaaaccatacct

>DpAV4a TCCCCAGACGCTGGGGTGAGGGATCGGTTTACTCATTTTATATAATATAAATG

>HvAV3e tttcaacaccctttaccgtattcacctgacgttatagtaaatattgtaacatg

>SfAV1a acaacagtgaacggtcgtacgacgggtctcggcagagtaaacattcaaaaatg

>TnAV6a cacaagaggtaattctgatttttttatatgtatgtttgttaacatacgtaatg

>CIV_118 gtatgttttaattgtaatgatttggtcaaataaataaagaataaatagaaatg

>CIV_458 tataactacaaggagtgtagatattttactaaccaagtcagaatactaacatg

>MIV tgtccaacgggctttacaggccgtaaaataacccatacaataaacaacagatg

**Upstream region of DpAV4a ORF041**

>DpAV4a CTCCGCCGGTGTTTTCGATCTTTACGACTATGGACTGGTTGTTGTCCGAATACGCCTTCTGTATATTCGTGTTGGCCACCTCGGTCACGGCCTTCATTAC

>HvAV3e74 tgtcacgcacagctggcgccactgccttttggcgtttagtacacaccgccatctcgctcaacactttgtagggtgccatttctttattaagcccatacga

>SfAV1a tccatcggtggatcttttttcgcgtgtgagtggtgtgacggttttgagccgtttgctacacacggacatttcgctcaacactttgtaaggtgccatttct

>TnAV6a agggctcgatgacgcctgaacaagtgggcatttttgaaaggtcacgatacatttttatatcttccgaataagaaaatttatacacacatctactatcgat

>CIV tatcatacccatcatcatctaatattggtaactcgtttatttctaaattagttgaagcattaattttagatattaaagtatcatacaaaatttgatattt

>MIV ggcttgtaaaactgtcaacactcatatttattcaaggggaaaatttaccgacaaattggagcggcgcaacaggacaatcgagagtttgcctccggggtca

>DpAV4a AATTCTTGTGGAATTAGTCGATATCGCAGATCCCATTTATATTATATAAAATG

>HvAV3e74 gaagcgccgccgtgaagtgaaaacacgtagagctgtaaagtaaagtcgtaatg

>SfAV1a ttatttagcgcatacgaaacgcgctctggtgcggtataataaaaatcgttatg

>TnAV6a acactataataaatttttaatacgttaggtctacggaggataaaaatactatg

>CIV gtgtgtcattttattgtttaataagaatcttttaacttttcttaaagtaaatg

>MIV agtccaacggttggactagggtttccctcgaccgagatggtaaataataaatg

**Upstream region of DpAV4a ORF043**

>DpAV4a GCCGCGCCGAGTCAAGGAATACAACAGTATCGTTCTTCACGTAAAGATGTTTTTTCTTTTGGAACGATTGAAAACGCAGTCGTCGTTCATAAAGGCGGTG

>HvAV3e78 acgtgccaaacttaagaacactaaacgtttcggtgttataggctacgccacgacatgctgcacatttaatctgacacgagctctcggcgtgacaacacgc

>SfAV1a ccagttcgtattcggttctcgatcgaacgtatagaaatttcagcggtaacattgtcgacgtatgtgagagtcgtcgtttacagttgacgtgctgtgtacg

>TnAV6a gtgatagtgttaacttccatatgccccgtgttcgatcattagcaatatatgggaaagcttaaactcaagttgttcactcgttacttaacactcatacttt

>CIV_393 atgtgttttcagagaacaaatctccataagcttcagtgggagcaaatgctgcatatccagatcctgacatgtctttattaataagttaaattagtttagt

>MIV_039 ttaaatttctctttccaactttaccgttctattggagctttaatcctatagaatgtgtttacggttttctgcctcgtctggtccatagtgacgcacaagc

>DpAV4a CTCAAAAATGAAAGTTAAAGAATTCAGATGTTAAATTAATAACCATAAACATG

>HvAV3e78 tctggtacctggggaccactcgcgggataaataaactaatttacttgaaaatg

>SfAV1a tttacacacccatgtcgaagggtgagtgtaaactgggcgaggtggtcgatatg

>TnAV6a ctggacgtaaacatcaaatgctagtctaagtaaattgatctcaaatataaatg

>CIV_393 taaacggaAAATTGAagctactaaaaggttttattcaaataattaatgctatg

>MIV_039 gcagaaatttgaaaagaaaaaattaaataataaagtaaattaaactcaaaatg

**Upstream region of DpAV4a ORF048**

>DpAV4a TCGCGCCGGTCTCCGACTCAGCCCTCCCTTCCGTAACGTATCTAGAAGAGATTATCAATTCGGCGGAACGAATTTACTTGCCCATAGAATACCTCACAAA

>HvAV3e aaactattccattcgcgaaagtggcacaattaccatcgtcatcgtcttcagatggtagctggactacgacaattctcgtcttgggagctatagtaatgtt

>SfAV1a catttcgacggatgacgacggtgcggtcgtgtgatacgctcgcatgatgcaaccgttcgtggtcatggtgtgaacgcgtgtttccagtatgtggtacagt

>TnAV6a tcgccggaattgctattatgatgttgtggattacagttaatgatggtgttaaaagataacaaacttattaatcctatgatagacgatttttttttgaaca

>CIV_224 aatacatttttagttccaattcctgttctatcgtcacgatcttcgccttgaaaaagaactttttctaataaatttaaatattgtttttcttcattattaa

>CIV_361 cttctttttggtgataaagatcgttttcttaataatggcatttatttagtattaaaatcatcattctagttttaaatttccaaaaaatatttagaactaa

>MIV_024 gctgccctcccccgtaagaatgagtttgcccgtactcgtttcctaggacaattatattaaacttttacgcttcttggtcaaaacacagcataaaagtttc

>DpAV4a ACTTAGAAAGTTTACCAGCAACGGCAAAATCGCGCGCAAAAAAATATTAAATG

>HvAV3e aggcgtttggatgtacaaacagtcctgaaatgtaaaacgtaacatacgatatg

>SfAV1a gtaagaatgagcagcgcagtagggtgccatgtaacaattgaaaaagactgatg

>TnAV6a taacaagtgatattttattatagtagttatgctagggaaaagaaaagaagatg

>CIV_224 tatccatctttatccattgatattttgtttttaaatattaaaataataaaatg

>CIV_361 aaatatttagaactaaaatcataaaataattttcaatttaaatcaaataaatg

>MIV_024 agtctaccatgtggcccgccagacactatagcggcttttggtaataataaatg

**Upstream region of DpAV4a ORF055**

>DpAV4a AACCCCAATAGTCTTTAATTATAAATGGAAATACCATGCACATGCGACTATTATTGCGAATGCGACGACGATTGTCAGTGTGATATTGACTGTCCGTGTG

>HvAV3e55 ccggtccgtagatataactatctatagtacctcgcttgttcacgtcagaacgtagctcgcttagagtcacattagataccactggcgtttctgggtttga

>SfAV1a cgggaccgtatatatagttgtcgatgttacccctcttgttcacatcctccctgacttcggtagaggtggcattctgagcggtcgtgtcggcagagatcgt

>TnAV6a tgtctcatagcctttatgtccactttgttcgtatcctccttggtttttgctacagcggttgcttcaatttttgggtcaccagacattgtgtattctttac

>CIV_143 ttattaaagaagcaccagacaggtttaaagcttttatgtaattgaacttttgattttaacagccaatagctgttaaaatcaaacagctagtagccgttca

>MIV_029 tcggtcggcgacgacaactttcccagcagcaccaaccacacctttgaggcgcgtcgtcgtccatcgtcgctgtcttctggtggtgctttgaaaccatcaa

>DpAV4a AGCTCTGTTAAATTTGATGTCAATTTAAACGGTTGTTAATTATCGATAAAATG

>HvAV3e55 agtcattgtaacgctttaattatttactacgtgaagatcaaatagtggtgatg

>SfAV1a cggtgtagacatatctaacgatttatttatttactacctgaagatcaaatatg

>TnAV6a ttttacttggattggatcaattgaacttttaaaacctacccaacatcatcatg

>CIV_143 ctataAAATTGAatagtagtttaagacatatttggtaaaataaatccaaaatg

>MIV_029 aaattttgtaaaaatgaaaattccatcgaaaaagagggctacaataaaggatg

**Upstream region of DpAV4a ORF064**

>DpAV4a CGAACACACCTGGTCTACGGTGTGGAACGACGATCCGAGTCTAGCGAACAACAGTTGTTCGGCTAAGTGTGCCGGCAAGCCGTTGGCGCAGATGTTCGCT

>HvAV3e aattaacgtatcggtgcggcgtcggtgacgtcaacttacttagaatgaaatagagcctaaaatcgcacacactcaacgtactgtgcagagtactttgggt

>SfAV1a acgattcacccgataacggtcccgatcgaaagacttcgaaatccggcactgtagttgtagacttttgaataaaatgtattttaacatgtaaacgtctggt

>TnAV6a actgtaaatagaagcgcaaaattcgagaaagactgcgctataatgtatgttttgatgtcaatgttgggacttggtgattacttgaattacgcgatgaaag

>CIV actgtccatatctattcctttttactgttttctttaattctttaattaaccttatttaatctgttcaattttaaagaagaataaaaattaaagaaaggct

>MIV ggtggacggggtagtagttgggatagtgcggcgcaccactcattttacaaacaatttctctctccagctggggtgaaccataccagctttgagggcaagg

>DpAV4a CCTTAATTTTTAACATTTTCTTAATGTTAAAACTCCAAGAACTTTAATAAATG

>HvAV3e cttaggaagttaattgcgtagtttaactgcgtacgactccactagtaaagatg

>SfAV1a tgttttattgcgttcaccccctttaacccattacagtaccactagtaaagatg

>TnAV6a tagcacttaaaattacccgtctctatactaaaatataccttaaataaattatg

>CIV gtatcccctttttaaaattttcaatcagaaaaaatgttgtattaagtaaaatg

>MIV gtttatggcttgatacatctttattattttatttttttccaacaataaagatg

**Upstream region of DpAV4a ORF065**

>DpAV4a CCTCCGCCGGCAGCCAGTGGCTGCGCTCGTGCACCTTCCACTTGTTGTAGAACTGTTCGTATTGGAACGGTTTGTAGTAAGATCTGCCTTTCAGTAATCC

>HvAV3e gtgtaccgtgtgtgggagcgaatcgcaagtagttaagcgaaacgaccatggttacgtttgtgctcgctgtaaaactctgaccgttacagaactggtgcta

>SfAV1a agttggtgttaccgtactcggcgaagttgctgcaccaaaatctacaggccatcggtgtgggatgtcggtacgtggtgaaacggcacgaccatcagtagcg

>TnAV6a aagattttcaaatggtgggagttaaagatttgtcttaaaactatgtatgaattattgtgtagaagaataaataatgtatagatagataactattgtttta

>CIV gattcaactttttttaaggaaaagtcaagttctttacaatgaaattccattatattaatttaaagaaactcataaaacaattcaatcatatttaaagtat

>MIV gattcgtaatagctctggaggttgaaactctccactagagaaatatctatttttaaaattttaccattttaaaaaaagaacatccgcaccattattttga

>DpAV4a CATTTTATAGTTGGTCCTGTCTTTTTAATTAAGAAGCCAATATCTATAAAATG

>HvAV3e ccatactctgcaaaactgcttcatcaaaatttgcaagctatcggagttggATG

>SfAV1a tgccccgtccatgccactaacgtaaaccttaatttcgtttaaatttacaaATG

>TnAV6a tttttcgcatggtattatcatgaatacaagtaaaacaaacaaacataataATG

>CIV ttttaaatttgaatgttttaaagggtttaaaccctttaaaacagaaaattatg

>MIV cggattgccggcgtcgttggattttgtccaccccattgccaagataataaatg

**Upstream region of DpAV4a ORF070**

>DpAV4a GTGACCTCATCCGCCTGCAGCAGAGGATCGCGGAGTTGCAGAAGAAGGAGCGAGACAAGAAGAACGAGATAAAGCAGATGAAGAAGGATCTCGAAGATCG

>HvAV3e cgacatgggttcgtttcgacgcgtgaacaccagagcgcaattaggggagcgctcgaaccagaagggaaacttttcgtacgtgctccgctcgatatcgtta

>SfAV1a caccacgagatacgaatcgggatcgttgcgacgcgtgaacatcagagcgtagttgacggtgtcttcgaaccactcggggaatttttcgtacgtgacacgt

>TnAV6a caagcaactacggttgcataataataaccatcatcaaaccaaaatggatatgaagtatgttgtatcatgatacgttcagtcattttacttacatacaagg

>CIV tggctgctttagcttgaggactcatagacgattcatcattaaaattatattttcgattcatacttctttatttattgtattttaatgttacatcaaggta

>MIV aacctttgttttcctctagatttttcttcattaaaaattgtcgctttttaatgaatgattcaatctttaaaattgaaaaactaaaattgaaaacggcacc

>DpAV4a GCGTCGAAAGTTGAACATTCAGGACAAAAAAATAGAGGAGACGATTAACGATG

>HvAV3e agttcgggatttgttttatagtacgccattacggttcagttctttacgtgATG

>SfAV1a tcgatagcgttttcggtgtcgtaagccattacgtttcaattctttacgagATG

>TnAV6a cattggacgttattttaaaaaaaaaattataaatctacggctttataacgATG

>CIV ataAAATTGAaccttatcttcattataaatacaataaataagaatctaaaatg

>MIV cctttcccatcataaattcaaccaa*c*aataaattcaaaaacaagtgaaaaatg

**Upstream region of DpAV4a ORF073**

>DpAV4a GAACTCGGTCCTCTCCCCGGTCATCGCGTCCAACAACATTCGCGTGTACGATATCATCAGGTACACTAGATTTTTTGACGCAAATTTCAAGAGGTCGGAT

>HvAV3e64 tgttgtagtgatgatttcgatggggcggtgttggcatgtaaccctccattttatgtcacgtctaattttcagcagtcgaattccggtagcgacaaagtcc

>SfAV1a ccaaagtacaaacgacgtcgtgcgttcaaaatgttcgacggaacggcaacgtttatacgttgaggtgtcgtgtccattttgtgcagaacagtcctggacg

>TnAV6a cagtagtcgcagtatgtgatcactggatagtagtagtaatactgatgataataatagttataataaaaacaattatccgttttcaaatcaaattccgaca

>CIV_428 tgggtaaaagcagagcctaaaaaagaaatatctgatgatgaagatgatttgtaaattccgtttcaaatataaagatttttcaatctttatattcattatt

>MIV_009 gttgatgatttgcaagtcgggatttgaggtgggaaattgtgtggcgattaattctgcaattggatcataaactttaaaagacatttatttactcgggctt

>DpAV4a TTTGGATACAGATAATTGAAGGAACGCGAACGGTATATATACGAATACAAATG

>HvAV3e64 agacacttgcaaaaggtccttatcttacgacggtcgcgtttataataaccATG

>SfAV1a tggctgtgataggaccgaacgtgtcggcagtcgaactaataataccgacaATG

>TnAV6a tcttctgagtacgtcgtgttttgtacgaagattgaaagttaaagacaataATG

>CIV_428 gattttaAAATTGAaccatataaaaagatagaatatattatttaattacaatg

>MIV_009 tctaaaattgaaatttcactggtgaaaaaaactttaataaactctccagtatg

**Upstream region of DpAV4a ORF085**

>DpAV4a CGTCAACGATCTGATAGAAGCGGTGTCTGCTCACCGCGCCAGACAGGAGGAAGAAGAGAGCGGTTATTTGGAGGTGGAGATTAGCATGAAGCATCACGGC

>HvAV3e146 ttcgtctgctatggtgggcgatgtttgaatggccaaaaatactattgtcatcgtaataacgatattgttgtgcatgatgctgcgagatttcatgtttatt

>SfAV1a gtcgatatttttactatagatcgatgcgaaaagcggacgaatcgcacgacgtcaaatgtaaagattacaacgacaatgaacacaagcgttctcagagtta

>TnAV6a tggtgaagatattattatacacaacgacggtgttgtcgccgataacaatggtataatataccagatggcttacggagacctagggctcgatccagaaaaa

>CIV_295 tataacaccagccaaaactgataaaatcatataaagatctaaaaattcttttttcatcttttatttactacttttaagaatagtctattttcttttttca

>MIV_016 tggtcgttgtaagctaaaaatgtaaaggttgtttttcctccctcggttggtggaataaagcttgaggtgcaccatacgttttcgaaacggtggtaaacgg

>DpAV4a GGCGATCATTAAGTAGTTCTAACCCAAATATTTGGGTTAGAATGTAATAAATG

>HvAV3e atagggtagatcgcaaacaagtcaatcgaagcgcgatcgtaccataacaaATG

>SfAV1a acggtcacacgttcacaattctcgacagctacacaccgagagtggtgtacATG

>TnAV6a acatctacgaatatagattacattattagaagtatcggcggtacgcctagATG

>CIV_295 tctttaatttagtaattttgatttagatttatttatttttaagaataaacatg

>MIV_016 ttggagcggccgggtgccgtgtaccgagggtgggaaaatgcatttaataaatg

**Upstream region of DpAV4a ORF086**

>DpAV4a TATTTGACAACGGCAAATACTTTTGCGTCACCAAGAAAGAGGCCTTGACGATGTTCGTCACGGGATACATGTACGTGTTCGACGGGCGCTCTCGTAACAG

>HvAV3e109 gatgcgcaccgaagataaacagtcgtaatagtaacagtgtacgtgtatatccctactgaaacttaacggtcggtatcgatatcgaccgttgccagtttgt

>SfAV1a caactagcacgtcatcggtctcgaaactagtctatttagaagatagtgacgaggaaaagcagcaaaagcaggaggatgagaaggtggatgaggaacaatc

>TnAV6a attttaaaacaaaaacggtcgtcaagaggcgaccgttttttggataagtaataaatttgtacttgttttatacgaaaacccaacttgtttgtctttaaaa

>CIV_075 cctttaaatatatactctatactatactctatactatactctatactatactacattttatgactcttattttatgttaccttattttaattttaatgga

>MIV_088 ccgcggcaactcgtactgtgtaaactcggacaggctgttgacgtcccggtcaaaggccggcgtctgaacggggtgatataatggtaggaaatcatacagg

>DpAV4a ACGTCGTAGAAGTTCGTGTTCGTTTTAAAACCGATCGCGGATATATAAACATG

>HvAV3e tttgtttttacgttgaataataaagtcacacatataaatcaaggaatataATG

>SfAV1a caaagtggacgttgaaaaagaaactaacaacgagtgatgttataccaaagATG

>TnAV6a ttgaaccctaagtattatctttggaattataaataaagcattaagtagatATG

>CIV_075 cattgtccattaaaattttttatttataagagataaatcaagaaataaagatg

>MIV_088 ttgtagtccatttatttaaaccaaaatgtggtttttttcaataataaaggatg

**Upstream region of DpAV4a ORF089**

>DpAV4a GGAGTCAAAAAAAACCGACGACCCAAACGAAAGGCGAAGGCGAGCGACCAACTCGCCGATCTGCTGAGAACAATGACCATTTAATTTATATGGGCCTCAG

>HvAV3e aacttgttacaaaaatccaaattaatgcgtttaaaatgtaattaacaaacggcgacctcactccctcttttgttctagtttgaaggtaagtgttgactaa

>SfAV1a agactcgtacgaaatgtgtgtcgaactcgacccatggccacgttctacgaccatcggtgacacgtagaaagtcggacatggtgaggaaaccgtccacgtt

>TnAV6a gatagacatggtggtacaatttataatgagctacaattttacaacggcatccagatatatatacactatcggagacgattcacgtcggcaaattcacaac

>CIV cactatgaggtgcttgtagataacctccacgatggtgcaccttgggcaacacaaaagtggcaaatcatctacattaaatttcaaagagaatttctctttg

>MIV aacctttgttttcctctagatttttcttcattaaaaattgtcgctttttaatgaatgattcaatctttaaaattgaaaaactaaaattgaaaacggcacc

>DpAV4a GCCCATATAAAAGTTGAATTAAACGGCGGCGTTCATTATAATGATCAATCATG

>HvAV3e agaccgagtgattcttaacattacgactcgtaacgtagaagcgacaggacATG

>SfAV1a caatggcgacgacgactggttcatggtgtggtcatgtgtgaccggaccgaATG

>TnAV6a aacattttaattagttttgttttttttttttaaaaaaaaaccactacaatATG

>CIV AAATTGAattgtccaatttaaatttaaaagataattaagcatactataacatg

>MIV cctttcccatcataaattcaaccaacaataaattcaaaaacaagtgaaaaatg

**Upstream region of DpAV4a ORF093**

>DpAV4a GTTTCGTGTTAAACGATAAATATAGACCAAATCATCTACACTCGAAAACGTGTGAGCTGTCTATCGACATGTTTATTACATTAAAATTTTTCTCTAAATT

>HvAV3e119 aggttcatgaccatatttatttgcaatacctaacctgaagccgattgtgactgaagccttttagaaattgatcattagtctttatgtatgcgaaaattta

>SfAV1a ttcagcaggagcgatgcaaatccctccaggtgcttgtcggccacgacttccgtatcgatgtcggccttgcacatctgtacgcgcgtcctgtcgactccgt

>TnAV6a gttttttttattaaaaaattgattgaattctgttgaattgaaaattttgtcaggatatgtactaatcgatcgaatagtgccttttagtcatagatacttt

>CIV tatataaaatatattgtatataaaatatattgtatataaaattttaatggatattatccattaaaatttttattgttaAAATTGAacccttatttataag

>MIV ctgtcggagtaaaacaacctaaaacgtggttcaatcggtaaatttaaggtttatggttcaaataaccataacaagtaaaattggtaagaaaaaaaatgaa

>DpAV4a TTCACCTCAAAAAATGATCCCAGTATTAAAATAATACTGGGATAAATAAAATG

>HvAV3e119 gagtgtgccattgaatgagtgttatatttgtctgtgatctctaaaacgtcATG

>SfAV1a gcggtaggaaaccgtcgggttcgttggtgaagtacgagtcgaagggaacgATG

>TnAV6a gtgttttaaaaacttaaaacgcagccacatatctgttggtaaaatacgaaATG

>CIV aaaagtttaaaaaattaatgaatataaaagaacactattatagttaaacaatg

>MIV attttcaaaaacaaaaaaaatataataaataaccataaaatccaacaagaatg

**Upstream region of DpAV4a ORF103**

>DpAV4a CCGGACTCGCTGTAACTCACACGCCGTTTTTCGCGGTGTTCAAGATGGGAATTTTTATAAAGACGATAAAAAGCCATTCAATGTCTTCTGGGGTTATCGA

>HvAV3e33 aagtattcaatgatataaacgtggttaaaggcctacgggcggcattgaacgcgtaatcagtatcataaggtgcggtcgtagttttacgaccgcacgtgtt

>SfAV1a acgaatcgtcggtgacgacagaaaggtgttccaagacgttaacgtggttcgtggactacgggccgcgttgaacgcttgatacacgtgtgttgagagcggt

>TnAV6a ctgattttaaaacaactaaaaattatagtgataagtcttctgatgatgatgatgatgatgatatgtaatacatgaataaataaatatttacatcactatc

>CIV_282 caagatttaaatttgaatacaatcagattactatttttaaatgcttttaaattttcatgtcttcttatttttcatgcaagtaaaaaaattagagaatagt

>MIV_079 aatttttcagacaattattcggccccaaaagaatgacgcgtcgagctcacagttggacgtttttcagtcgtcagttgtagggttcgcttgggccacaaac

>DpAV4a AAGCGAGCTCGAGGTGGAGCTGTCGGATCGCTTTTCGGCGCTTACGGACGATG

>HvAV3e gagagatcgggtacaatagtaaaaaggatcgatattatacgaccatcaaaATG

>SfAV1a cgcgatatttgcgaaccgcaagtgttgagagatcgtgtaatctttgtaaaATG

>TnAV6a gacttttatttcaacatcatgtttgaaagagaatgtattgtaagtaaatcATG

>CIV_282 tacgatcgcgagagttctgcgtagtttaaaaaataaataattaaaagaaaatg

>MIV_079 gtccaaatgattgattttatagggtaaatacccaaatatataaactgacaatg

**Upstream region of DpAV4a ORF108**

>DpAV4a GAGGAGCGAAAAAGGGACAACGTTGCACCACTATTTGTAAAACTGGCCCGCTTTGCAGCAAACACAAGTAATTCATAGGTTTTGTTGTTATTAAGACCTT

>SfAV1a tccggacggttccgtgaccgcatccaacggtattcgaatatcggtattgtatatcataccgtccatcagtctttttcaattgttacatggcaccctactg

>HvAV3e gtagagatgaactcgtagactttttccggtgtgagcacaacgcgcggtagtatttgcgaaataaaaccgattctgtttaaagttacgtaagtcatggtaa

>TnAV6a ctggaattaatgaattgttttataaaacatggacaccacataaagattcttttaatgttatactcgttgattttccttcaccgttgcttgttgacaaact

>CIV tatgacgttcttgtctatcattaagtttttttctaatttttttatctatcacgaagtcctttttttaaaatttttaccaatacaagtcgtattaaaacag

>MIV ctggtgccccagtcggagcggcggggaaacgtccacaactctttggtgtgtttaatctcgttgtggacgacgaccgattcgtggtcgtctttaatctttt

>DpAV4a CGGGTCTTAATAATTGATGGTGGTTTAAAAGATTAGATGACTTAACAAAGATG

>SfAV1a cgctgctcattcttacactgtaccacatactggaaacacgcgttcacaccATG

>HvAV3e caaaatattaagtttacatttgtcagacgcaagtaagacactgtacagtaATG

>TnAV6a gcagattcgtattccttgattggaagtattagagaagagacgtaaccaaaATG

>CIV ttaaaaatgaagcattgttaaataaatatttcaacaaacaaacatttaatatg

>MIV tcctatccacattggataggtcaattttctggtagattttaccattagacatg

**Upstream region of DpAV4a ORF116**

>DpAV4a CAGACAGAAGATGAAGTTTTAAACACACTCACGAACTGTCCGTTGACCTTCATGGTGAATATTTTTCTTGGTTCGAAAAAACGACATCTTCATTTATAAA

>HvAV3e gtactacctctgttgttgtgattgtataaataatatattttatattttgtttaactaataaagaaaactttacgtaatgtactccgtgttttttcattac

>SfAV1a attttgttattgttgtagttttttttttttttttttcgtaaccaaatgtatatgtatttatgtagttacataataaattcttgcctcgagtaccttgttt

>TnAV6a attaatttggtacaacgcttcgttagccatttactataggcataagataaaaaaattattatttattaaactaaaattgtacgtttgaattgtgttcttt

>CIV aattcatttcttgattcattgtctttttttatattttgtttaacacacttcaattttaaggattgatgcttttaaatgaaaaatatgattaaatgaacaa

>MIV gagcttcttttcccgcattgaggagcgagacattttgcgctacgttaggttttgtttgatccataaactaaagtagggcgataccccctccaaccggcac

>DpAV4a ATCGTTTGAATTTTGCGTACACACGTTTCTCAGACTTCTCGTATAAATAAATG

>HvAV3e tacattcacgcaaacatactccaatctgccgatgataaatagaagaagttATG

>SfAV1a catttcgctcgtaattcacaacctatccgacgaatgtaaagtaaaaagacATG

>TnAV6a tattttcattcttttcctcccgtatgttatctaagtaaaagttaagtataATG

>CIV ctatggatataaattttataaaaatatttccttaaaaataaataagaaatatg

>MIV gttttgcgtttagttgtcctgcaactatgggaaaaaatttcagataataaatg

**Upstream region of DpAV4a ORF117**

>DpAV4a GTTTTGTCCCGGTTGTCGACGCCCAACACCGACGTGGCCGCGAATTTCGTATCCGGGAACGCGAACGCTTCCATTTCAAACCTGTCTGACAATTTTAAAG

>SfAV1a agtgcgcttacgacgtgagtgccgttcataagaacacgtaatctagaaacacgatgaaactgcgcaacgcatacatcacacccaaaacgatcaaaacgaa

>HvAV3e110 gctgacaaaaagaaaatcgaggcgcaatgcgcttacgatcaaatcaacacatagtctattacgatgacaacactgcgaataacgtataaggcaatcaaca

>TnAV6a atcttttgaaagatcgacttcttcgacagtccaaaacgatgcttctgcttttttgtacatatcccatatgtcttgatattgaattggaaatatgacaaaa

>CIV_355 tccatccgaaacacgggaaagtggcctgagaaaaattcctggtctcaccggggtttgaacccgggacctttgggttgccatgccagcgcttaacccactc

>MIV_104 ttatttatattttttttataatttttcactttccaactctctggggtcaacgttctcaccgttcaagtccccacccaccataaagcatttcaattgtttt

>DpAV4a ACAAAAACTTAGTCATTTATATTAAAATATTTCGACAAACATTGTTATAAATG

>SfAV1a catggttttctttttctacaaacgtacgatccgtcggtcatttgtaaaacATG

>HvAV3e cgagtattcctatcatgttttatactgtgctcgttggacatttgtaaaatATG

>TnAV6a cgattagggttatcttttagtaacggctctagttgtgggtcaaacaacgtATG

>CIV_355 agctacgccacccccaagaacgtcataagaatccgtaaataatgtaataaatg

>MIV_104 cggtgcacaatgttggttgtgtggtgtttgaaaggggcaataaataaaggatg

**Size variations of the intergenic regions located at the 5’ extremity of each of the core gene in the 6 virus species**

**S7c: Conservation motif analysis within the sequence of the 150-bp located at the 3’extremity of the 28 core genes shared by ascoviruses, DpAV4a and invertebrate iridoviruses.**

**Method of analysis**: Repeated motifs in the 150-bp downstream the stop codon of DpAV4a, SfAV1a, HvAV3e, TnAV6a, CIV and MIV genes were located using the MEME software at <http://meme.sdsc.edu/meme/intro.html>. In the sequence alignments, stop codons are typed in white and highlighted in dark grey. Putative polyadenylation signals are boxed and ARE motifs are bolded. The conserved and few degenerated CPE motifs were highlighted in yellow[[4]](#footnote-5). Enlargement of the CPE motif conservation at both ends were highlighted in grey. The conserved motifs located in 100% of the sequences were highlighted in blue and green, in spite of the fact they have no sequence similarities to each other between genes. Sequence stretches types in red correspond to palindromic region detected with MFold. At the end of the 28 sequence alignments is a table describing the length of each intergenic and untranslated sequence of region and the orientations of the core gene (1) and the gene downstream (2) in each genome. For each virus, when the core gene and the gene downstream and are not in the same orientation, the cases are coloured.

**Downstream region of DpAV4a ORF001**

>DpAV4a TAATTTTGTTTTTATGGTCCCTCGGGACCATAAAATTACATATGCAGTGTTTCGACCGTGCGTTCGAGCTCTGCGGGATAAACGACGAACAGTCCCAACA

>HvAV3e taatgaataaatcaggacataaattttaacggttcttttatttcacaatcttccaccaaggcaatcttgagattggttcggacggttcgccaagtccgcc

>SfAV1a taaaactacaatgggtacaaaacaaaatttgttttatttcattttctcaaatacacgattcgatccaccagggtttcctcggtatattcctagaagtcca

>TnAV6a taaaattgaattataaaaatgtgaaagtaccacaaatataagtatttttttttattgtgaatttgtttgttttttaacaggataaaagatggattcgtta

>CIV taaattttagaagtaaagtttagtagtattttgtttaattttaatgatagattatcattaaa**attta**attaccaatgaccatctcctcccactcctccta

>MIV taaataacaccgacgcacccaacaatccaatttctacttttttcttttctggtacaagttaccacaaaagaaatttgcggcagagtttgactgtcggagt

>DpAV4a ACATAAGCTCTAACAGAAGTCCGCTCGTGGTCGTTATTAGAAACGCTGCGAGA

>HvAV3e cgactgatccacgaacctgtagacacacaacacgaatacatgttataacccac

>SfAV1a catgcgatccgcacatgatgtaggtcgcatctcgtcgaaccgttgcgccgaca

>TnAV6a acttataaattacgttatcgtttgaacaaaatatcagatgtgaaagaaagaat

>CIV cagtatataaatcatttcctatataaacacctcctaattttgattctagaata

>MIV AAaacaacctaaaacgtggttcaatcggtaa**attta**aggtttatggttcaaat

**Downstream region of DpAV4a ORF003**

>DpAV4a TGAACAAAAATATTTTTATTATTAAACATCGCTTGCGATGTTTA**ATTTA**AAAGCGACTGTTCAGATATAAATATGGATCAAATCATATCTACACTCGAGA

>HvAV3e tgatccatggtggcggatcgtgttggcaacgaaactacgaacggcagacacatgtcctctatcacttctaccgctttgaaacgccgacgggaacacgttc

>SfAV1a22 tgaccatcataccacaactggcggaatacacaccgaagagttcaatatcatcaccgcaacaacaacacgacgacgataacgacatgaacacgacgcggga

>SfAV1a23 tgacacggctatgacggtaacgaaattaatttcgaaaatatcgcaccacgtaaacggcgaacacagactttgtacggtggggtgtgtgagtgtgtggtcg

>TnAV6a tgaaatgaagtgttctaaaaaatcgttagaattaataaattgtgaccactgggtggggtggtactgtaaacattgtacaataacaatcgataataatgat

>CIV taattgaacttttgattttaacagccaatagctgttaaaatcaaacagctagtagccgttcactataaaattgaatagtagtttaagacatatttggtaa

>MIV taaggctttcctctccattgtttttattatttttaatgatcgatttgggtcattaaaaattaccatctactcaaagtatcaatcgaccattacttttgaa

>DpAV4a AAGTGGAGCTGTCTATCGGCAAGTCCAACGACGAGCTGGAGACGGGCTTCACG

>HvAV3e aaacctcggtcggttgtaacagtgtttggtcgggtttaatgtgtaaaaagtgc

>SfAV1a22 atcgagcgaaactcgagctcgactactgttggccacgtttcgagtcaccacac

>SfAV1a23 ggcgtcatgtgtaaagtatgtcgatgtacttggtcggacggtgaacgtgaaca

>TnAV6a gatgatgatgatgataataataatagtgttcatgataattcccatac**attta**g

>CIV aataaatccaaaatgacaatcatatgtattgatggaataattggtgctggaaa

>MIV ggtgcaattccccgggctaccagtgcgttttgccggcttcgtttgttgaaact

**Downstream region of DpAV4a ORF008**

>DpAV4a TAAACAATTGAACCAATTTTTATATTTGATATCGTCAAATATAAATATGGACGCAGCCGTTCTCAGACTCGAGGCCCGCATATTGGGTTTAGAGATGGAA

>HvAV3e tgatagtgacgggcttctcaaacgccatcgcaattggttcgccttgggtaccaccacagcgtttgaatactgtcgtacaagcagtagctggcgttcgtta

>SfAV1a tgatcgtcgaaggattcacgaacgccgtgtcattgggcccaccatggataccaccgtatcgtctcaaccaagtcgtagaaatattgtcgaatcaacaata

>TnAV6a taaacatcgatgaaaaatattatcccttgaatgacttattagacacagctatggttatgtttgaaccagattattgttataatttcattaataaaatagc

>CIV taaataattttgta**attta**attttgtaattttaatggaaatatttccattaaaattaaattaaatgttata**attta**taagaaaacgtgcttatcttaaaa

>MIV tgaagggttggtaaaatgaatttttttccaa**attta**acaccataataaaaaaccatggattacgcgttggagcaagcaatcaaaactaacctcgtgctgt

>DpAV4a GATGAGGTCGGCGAAGAGGCCGACGTCGCTCTTTCAGAATGGGATCTGTTTCT

>HvAV3e agcgttcgtcacttttgttccagactcgaccgacggtcgagtctgatatggct

>SfAV1a gacacgcaacgaattagactcggtcgtcgatcgagtctagttttgagaaaaat

>TnAV6a ttaaaaaaaaaaacaattatattgatagactcgattaaaatcgagtctatctt

>CIV a**attta**actattttaataaaataacaatcatgaatcgttcagataaaattacc

>MIV tggtgga**attta**tcctagaaaaacacccaacgattgaacgacacaccgtgtac

**Downstream region of DpAV4a ORF009**

>DpAV4a TGAGACCTAATCTAGGAAGAAATTTGTTTATCAGCCTGTTTACAGAAAAAAACGTCAGTTCCTTGCACACTCCGAACGCAAGACTCGTACACAACATCAT

>HvAV3e taattagatcccatttgggtgcactttcaaacgctcgagttaaactcgagcgttcagttgccaacagcaccttcgccaagatggaccgttgcatcgagga

>SfAV1a taacgtctgtgctgtttcacttgctcgtcatctccgaagtgtccaagcggttgtcgcctatccgtcccgagtcatcgtcgtttttctgttgcatcatacc

>TnAV6a tgaaagatattatgatttggtggtgataaaacatcatatggaccagtattcttactattactgtacacatcgtcatcttcatcgtcatcggtgttttcgt

>CIV taacatcttcctttaatataacatcttctaccttaaggttcccacctgaatattgtccataagaaaataaaaatgaattatgt**attta**aaatgtaaataa

>MIV taagttgccgcaaatcataaaacagtacaacccattagtacaaatttttttaggtttagttattgtacc**attta**accataaacgcgcgcctagcgttaaa

>DpAV4a GAGATTTCTCATTTTATAAAATGAGAAATTGAAAACGTCCGCGACCCTTTCCG

>HvAV3e atgggaagacgatgtcatggatattgttaaattagaactggatattgttgaag

>SfAV1a gaaaatactactcagtcccaggaaacgtccaaacacgaaactaccgtatttcc

>TnAV6a agtcgtcgttgttgttactcctaccgtgttcaccattagtatttttgttattt

>CIV agaataataaagatgtttgcaattaaaatagataaaatctctaattctaattt

>MIV attgaaattttaatccaaattaatgcccaaataaaatattaaacacgcgacat

**Downstream region of DpAV4a ORF010**

>DpAV4a TGAGGTTGTGTATTTTTAGACCCCGGGGTCTAAAAATGAAACCTTTCGTTCCTTTAAATTAAGACAAAAATGTCTATCGAAACAGTGTTTTCTATTTGCG

>HvAV3e tgagttgctcacgtgcgatatataaagctgacagtcatggactcgtataaaatggcacacatagccgtggaagtgagcgctgcttgcgctcttgcaatgt

>SfAV1a tgagtcgttcccatgtgttatataaaaaagttcagctatggactcgtataaaatagcacacatcgccgtcgaaataagtgcggtcggcgcattggccgtt

>TnAV6a ta**attta**tcttcgttattattaattacatgttaaaaatattttgttgttttttttttttaaaaaaataaccgatttcagtagtaagactgcatcgggtct

>CIV taatttttaaaagatggtttgtatcatatcaaatgt**attta**tttttaatggtaacaaccattaaaaatttacatatttgaagttcctaataatggtatag

>MIV tagaaagagtcaaacaaccattaaaatttttaatcctgtagttgaggattaaaaattactcggttcggcgagtttaggaaaactttaggtcctttaggtc

>DpAV4a AAATGCGACAGGAGGTGATTTCGTATCTCTCTTTGTCGGATTTACACAACCTC

>HvAV3e acaccaaaaagtcggtggccagattagacaaacaaatggttgacattatgact (No palindrome found in the 150-bp)

>SfAV1a tacacaagaaagtcggtgaatcgcatagagtccagaatcgactctgtcatatc (No palindrome found in the 150-bp)

>TnAV6a taggatggtaaatacgtttaataaaccttaaacgatggatacttcacgaatgg

>CIV gtgttggtggaattgtcataattggatacttaggttcccaaggtcttacagga

>MIV catctccagctgcgtctccaggtccagctccgagtgcgtcagttcgtgaatgt

**Downstream region of DpAV4a ORF019**

>DpAV4a TAGTTCAAATCATCATGATTTGAACTACTATTTCTTAATCTTTGTCTGAACACCGCACGACGAACTGGCCACGACCAGTTGTCTGTCGGTCAGCCACTTG

>HvAV3e taatcgtttgacttgatatatacagtcgtgtaaacacgactgtaatacacggtattggcgacaaaaaccttaattacagttcacaattgtaatcgtccag

>SfAV1a taatcacacacacacacacacacttgctttgtacagtcgtgtcaaacacgactgtagtaaccgaaatgtgaccatactattcgtcgatcgagtatctgtt

>TnAV6a taattttccgattata**attta**cagtcgtaaaaaattacgactgtaaataaatataaaaacaaaataaaccataatgaatattatagaagtagcagaagaa

>CIV tagaggggggttggggttggttttattatcattttttaatggaaaaaattccattaaaaaattattgtgatttttcaaaacgttctttttttagagtttt

>MIV taatggccgtcaacaacaacattgtgcgcatctcgggcggctgccttggcttcccagtgctgtaaacgaacgtgcctataaacaaccatacaaccgattg

>DpAV4a GATATGGAGTCGCATACCTTGTCTCTGGACTGGCCGCCGGCGTCCAACTGAAG

>HvAV3e actgacatcgggataatagtccggttcgtaccgatttgcaaaaaactccgcca

>SfAV1a cgcaaagaattccgccatagtttcccgcctgtgtatacgaacgcgtatctcgt

>TnAV6a gatacgacaatcactaaaataataaacgaaaataatgataatacaatacattc

>CIV ttttagaattatatatccataattctatatcatatccactttctgttacagat

>MIV tccgccactttgtggtttgattaacttttaaattttttataaaacttaaaagt

**Downstream region of DpAV4a ORF020**

>DpAV4a TAGTAGTTCAAATCATGATGATTTGAACTAATGAAGGACGGGTACCTGGGCGTTGCCTTCGGAAATCGTGATTACATTCGTGTTTTCGGCTATAGTGATG

>HvAV3e taaacgcgtatcataacataatcatatgatatttttgaggagtatttgcctaccagactagaataacaacaaaagaaacgcctttttcattgtgtcacgg

>SfAV1a tagaaaaaaaaaaaacacattacaacttggtgtacaatt**attta**ttcaaagtaacacaataccatcgcaagaatccaccaaacgataattgccgtgtcga

>TnAV6a ta**attta**ataatatataacaagagagtaaaatcaaatatgtcgtctgaagttgttcttttacacaatatgaaaataacggatcttaaagagaatgatgta

>CIV taaattttaatacctttaaggtattaaaattaattattatttgaatcatacgataaactatattgggaataaaattgaactaattcgtccatacaaatat

>MIV taattatggcttttataccttgaaattaaaaaaaagtactttggcatattttattgtttttgaatggttacaatacaccattcaaacacacgccttccac

>DpAV4a AACTCGAACTGGCGGTCGCTACGAATGGCCTCTCGGTCCACGGTCAACTCCAT

>HvAV3e cacttaacctcctaagacccgggctcggatacggtgcatcggacagtgagccg

>SfAV1a a**attta**acgtttacactcaacgattccacatcggagtaatcttctccttcgtc

>TnAV6a actgatgtgacagttttccgcaagatggatgctgaggcgcgagagaaagcccg (No palindrome found in the 150-bp)

>CIV aatattctctaattgcatctccgtttttcgt**attta**atttcataattattttt

>MIV cggtcgttgtaagccttaatattctacaatgttgtctgtccaccgtttgggac

**Downstream region of DpAV4a ORF022**

>DpAV4a TAAATTTTTTAGAAAAGAGCTTTTCTAAAAATATGTTACTATAAAATGGACATCAACACAATTTTAAACATTGTAAAGACGAAGCTCTTTGAACCGCGCA

>HvAV3e taagtgaaatctcacgcggtctattagcacctagatgagcagcaagatgaaaggcatgattaatatattgctatcgtttctaatacaatcgtcgcgttta

>SfAV1a taagaccacgatcatcgttagcaccgtgtat**attta**gacatgacgttactaattgacgttattgcttaaccacgacgatcgtcgagtgttcacgacgatc

>TnAV6a taaaaaaaaaacaggaactaatggtacattttgttctctaatggtctgtagtcgtcgagaaaatctcgacgattacagtgctgtcaatggtggtacacca

>CIV taatttttaatagagcatgctctattaaaaatacaatacatgccttaaaactatcatgccttaaaactatcatgccttaaaaatatcatgcattaaaact

>MIV taatggcaagtctgctctaattttttaatggtacaagatactattaaaaaataaaaactaccgcttacggtcagcctacactttaaacatgttaaactgg

>DpAV4a AAAAGGTAGTGAACACGGTCGGTGGAAAGAAAATAACCCTTCCGTCACCGAAC

>HvAV3e acacgacgattggtgcatccccacgattcgcatcaatggaggagttccgagac

>SfAV1a ctttcgcacaaaacaacggtgtaccccaacagtgagccaactgcgagtacgnn

>TnAV6a taacagtgcaccagactagggtacaatttttcatcaatattatcaatatcnnn

>CIV attttcccacattttgtttacggccaatcatatgaccaataatcatcgaatcc

>MIV ataaacgtttggagggatgggtcggggctggagggcgactgcgtgtcgggctg

**Downstream region of DpAV4a ORF026**

>DpAV4a TAACACTCTCAGTGAGAGTGTTAAAAAAAATTAAAACATACCGGCTATCTCGGCTTCGACGTCGGAAACGGCTTCGTTTCCGAATATAAAGTTGCCCGAG

>HvAV3e taaatgagtgaacgcacaactaaagtgacacgttcgaacaaatcgtccgctgccgatggtggtacagcaacatcacgtaaatctatattttcgagcccca

>SfAV1a taactccaattgaatgcgtacgtctcgtattgtaaatgagtgaacgtacatcgaaaggaggagttcgtcccatgaaacgaccgatgtcgtctagaaagtc

>TnAV6a taaataaaaaaattattaaaatatattgtgtcttgttattcgtactgttgaaaataaagacctcctattataaatgagcaatcgagctatcaatgaaact

>CIV taaaaattttttagaaaaaggaattaaaatacagtttaaatttgtacaagaaaatattaatactaaccatgcagtttatgaaaacaataaaacctttcca

>MIV taggagaaaccattatttttaatttttttagaaaaattaaaaattcaacaaatggagtacgagtacttccattcgagtatggaaaagagctttttgcaga

>DpAV4a CAGACTTGTTCAAGGAAAAAGTCTTTTAGTCCTTTGTTGTTCAGGACGACTCG

>HvAV3e taccaaacctgggattcgtggctgccgagaacggacatgttaggcgtcaagca (No palindrome found in the 150-bp)

>SfAV1a tatattctccagtcccattccgaatctcggtctcatcgcagcgcccgagtcgc

>TnAV6a gcttccaaacaacgacatgtaactaccaataaaacattgaaacataatgaggt (No palindrome found in the 150-bp)

>CIV acaattttagaacagttaattaaaaatgaaaacaacgcttgtttaacagaaat (No palindrome found in the 150-bp)

>MIV tggtgtggtggaaccttttcctttccgacttttttagggtcaaaaagtccata

**Downstream region of DpAV4a ORF033**

>DpAV4a TGAACGGCCACATCGAATTAAAAGCGCAAAGCGATAAATAAAAATGGAACAAATAATCGCAAATGTACAGAACACCGAGCTGTCGTTCGGAAAGTCCAGC

>HvAV3e tagtgtaattgatttttgtattatatgctaccaataaaagcgggaaagaagcacaaagtctatcatttcataacaatccgttcagtagtaaggactaata

>SfAV1a tagccaacgaaatacgagaatgggacctgaacgagatcgccgcaaacgcgtaccacagtggtgttaaacaacaacaatgtatcacggaggagatacagtc

>TnAV6a tgaatattattcgttgtttttatcataatcttcttaaattgcttattataacattgctcggattttgattaaatatattgtaaccgtgggtggcaccgca

>CIV taaaatga**attta**aaattataaa**attta**taagaaaacaagttggctttacccttaacggtgtaagatttctaaagaatgaaattggaaaaccactacaag

>MIV tgagcgccaccatcaactacaagggcaaccgcgtcgactacaagtcgctgagtacgggagagtatgctcgcgtcaagctggcgtttgacctaacgtttaa

>DpAV4a GACGAGCTGGAGGCGGGTTTCACGAAGACGCTGAACGACGCCTTCTCGTTCGA

>HvAV3e tctaccgacaggttcatgaccat**attta**tttgcaatacctaacctgaagccga (No palindrome found in the 150-bp)

>SfAV1a gatacgaaaccgaatacaagcggtcatgtataacatgcgaggtgtaaactcac (No palindrome found in the 150-bp)

>TnAV6a tgtgtgttcgctcgcttactttaataactcaacagtgtggagcatacagaata

>CIV gaaggtaatatcgttctttcaaaacaatttgtacataaagatgtggaggtttt

>MIV ggaaattttgggcgagacgataataatgttggacgagtgtacggccaatttgg (No palindrome found in the 150-bp)

**Downstream region of DpAV4a ORF036**

>DpAV4a TAAACATGGTAGCGCGACTGATTCACGAAACCACTTTCGAGAATTTTGTAAGTATACTCAAAAGTAAGATGTTGTACAAGCCATCCGAAAACA**ATTTA**GG

>HvAV3e tgaccgtacatgtaacgacagttaatctgtcccattaaatacggtcgtatgtatacgaccgtactttgactacgctctctttgtataatgtatatttgtt

>SfAV1a tgaggatatctgatgggtgcgattaagcgttgtattcgactaccgttgaattcgtccaacgtacaactggtcaccgactctatggagcaggagtttggat

>TnAV6a taaaaggttaacgatacggtcgtattgtgtacgaccgtatcacggtggaggtgaataaatgatagtctgatgttttttttttgttgaaattgtgtttttt

>CIV taatttttaatgaataagttcattaaaaattaaattctaatattatagttttaaagatcaaagacctttaaaataataatttcttctattctgtgagatt

>MIV taagtattattgtgcccgtctttagattaaattcaacgtttaaatttctctttccaactttaccgttctattggagctttaatcctatagaatgtgttta

>DpAV4a CAATACGGTACAGGGAAGTCGTAACCGAAGAATAGTATCGGACCCCTTTATAA (No palindrome found in the 150-bp)

>HvAV3e gataatattattaaatttgtctgattttatgatgaaatgtatttttatggact

>SfAV1a tgaaacggtacgatttgaaatcgtgcgtacaacaattggttcacgcgttgaga (No palindrome found in the 150-bp)

>TnAV6a ttattagaaatctgcatctagtgtaaatacattgtcaagtggattcttattca

>CIV tattttgtgatatgaataatgt**attta**attttactttgatcttcgttgtatga

>MIV cggttttctgcctcgtctggtccatagtgacgcacaagcgcagaaatttgaaa

**Downstream region of DpAV4a ORF040**

>DpAV4a TAAACTCTTGAATTGTA**ATTTA**AAATGTTGACCATATCGATTGATGTCGGCATCAAAAACTACGCAGTTTTGCTTGTAGAGAACGAGAGACCCGTTTTGG

>HvAV3e tgagaagttttgcatgtttatcgcaacgatgtttgattctgtattccaggctgagggcttcgctcttcgtgaaggattcgtcgctacgccatactaattc

>SfAV1a tgaacccgagctgactttctacgatgcgcccaccaccacggttccggcacgctgacggcagtcgaataggtacggaaatcgtcgcacccaccgatgtcga

>TnAV6a taaagatggtattttttttcatatgctcgggttgtaactcgagcatatagaaaaatctattacatcatcataggataggctttagggccgtatccaccac

>CIV taattcttaaa**attta**atggtaaaaaccattaaattt**attta**attgtcttttaagtaattttataatatacattaataagtgtaactgttaaataatttg

>CIV4 taatgcataattttaagctatattttaatgatttaaatcattaaaatatattaaattaaatggaagttggatgtgtttatataataacaactcaattgta

>MIV tagggcttgccgtcactttaaactcggaccactgaacgcgtatggttcattggcactttaaacttggcaaaaatgtggcaccgaaattaaaattgaaatt

>DpAV4a TGGTGAACCTGAACATGATGCCCTATTCTGCCCCGAAGCTTAAAACCGAACTG (No palindrome found in the 150-bp)

>HvAV3e gagttcagaggcacccctcagacattttgcacctttacccgagcaatgcgttt (No palindrome found in the 150-bp)

>SfAV1a atcgaccattaaaggtggtagtttctcgtgttaccaatgcagcgagggtgttg (No palindrome found in the 150-bp)

>TnAV6a catatgaatcgttttgacacgctctggcgtttggaatagtttcacaagaagct

>CIV ttagagaataaagatatgagtaaccaaaataaagttctttctcttggattatt

>CIV tgaacctttagatatttataaaataggatgtacgaaagacattaatagacgat

>MIV tttcatgataaaaaatatcataaataaactatacaacaatggaagcaaagaat (No palindrome found in the 150-bp)

**Downstream region of DpAV4a ORF041**

>DpAV4a TAATATAATAAAATGCACAGACTTAAAATAGATTTTGCAGAGAGGCGGTACAGGGACGACGTTGTTTTCGAGTTTCAAACAGACAACAAGACAGTGGTTG

>HvAV3e74 tagaaatactgacaagtaaatggaccaagtacaaaacgaatacgtagatggcatcgacgaacaagacgccgacgaagaagaggcgtacatcggcaggcaa

>SfAV1a tgagcacgatgaacgaagacgacaacaactattattccgagaccgagtcgataggtggcgatgatgaagactacgccggtcgtcaggagtacgaggacga

>TnAV6a tgaatatggaccaaagtctttatttgtcattattacaagaagctgacgatattatagaagaagagatgaccgacgacgaagatgaagatggagaagcagg

>CIV taattcttttttaatga**attta**aattcattaaaaaatagaagaagaaagaaaatatatttcaaaaaagttaaataaatccagtcacttttatacgagcta

>MIV tgaagacagacaaccttaaaacacgtgtcccgttgttggttggttgtggagcaaagtaacccgtaaaacagtcacctacacaccttgtaagaatgggggg

>DpAV4a AGGTAATCAGGTTGGCCGACGAAACCTGGTATTCGTTAAAAAATATATTGGAC (No palindrome found in the 150-bp)

>HvAV3e gactacgaggacgacgattacaacttcagggatcagccgatggccggtgccgt (No palindrome found in the 150-bp)

>SfAV1a tgactttaactaccgggacctaccgaccgcgggtgtcgttaacgagacgaacg (No palindrome found in the 150-bp)

>TnAV6a ag**attta**gatatatataatactatcgaacagtataattatggagataggccga (No palindrome found in the 150-bp)

>CIV attatacaagtctaattagtccttatgatacaatgatatgtaaagaaggtatc

>MIV tgaggaataaagcttggggaaggaggttgttttcaccctcatttcccccagtg

**Downstream region of DpAV4a ORF043**

>DpAV4a TAAAGGTGTTTTTTATTTCTAAGGACTTCGGTCCTTAGAATGACTGT**ATTTA**TAAATGTTAAACTGTGCTTGTAATAACGGCGACGCGCCATTGACCGAA

>HvAV3e tgaattggcaattgatggttgtttaaatataaatagtagataattaataccaactcaaaatggcaataaaagaaaaagtacaacaaagagtgcaaaaagt

>SfAV1a tgattgtgacatgttccacctctcttatgtataaataaacattagtgtaaaaattgcatccgatgaagaaacaaaaccggtcgtaggggaatcagcggtt

>TnAV6a tgatgtttgaaataacgatgattaagattcatatataacgggtcgggttgacccgacccgtttatgattacgtattaattagaagacgtcgaagtagcac

>CIV taagataaatattctaaaaagccgttcggaaccggcgttaaactgtaggtccaaaaaaaattaaaaaaataaaaaaaaaataaaaaaaaattaaaaaaaa

>MIV taaatacacacaatacca**attta**accaca**attta**accacaatatatacaaccca**attta**accacaatacaaccacaatacaaccacaatatacaaaccaa

>DpAV4a TACATCACGCAGGCGTCGTCTTCCGTGTCCGATCAGACCGCCGGTTCGTCTAA

>HvAV3e acacggttggtggtggaggcgatgccacgacttaaagatggcgttcgtgcact (No palindrome found in the 150-bp)

>SfAV1a ggtccgacgtagatgtaacgacataaaaatggcactcatcagcatcttccacg (No palindrome found in the 150-bp)

>TnAV6a ctgatggtggtgatgaatatgatgatgattctatacatttggataataaatac

>CIV aattaaattttaatggcattagccattaaaattttaattacatttttcgatat

>MIV cttttttaatttttcatggtcacaatggccataaaaaattttcacattcaaga

**Downstream region of DpAV4a ORF048**

>DpAV4a TAAGGTTGAATAATATAAAATGAGACAATTAATTTCAATGTTGAGAGACGACGGTACAGGACAACAGCGCGACGAGTCGGCGGCGTCACACGAGGATCTA

>HvAV3e tagtgcctcggttcatttcacccctggccacttctatgagcatttccgtgctaccgacttttagttgcggtaaat**attta**acatcgagtacaccgtttag

>SfAV1a taacgcgatcgctcgcgcttcctcgaatttcgtcaaacgagcgttcggtatgatgactagatgttcgtcgatgacgccgtccgtttgtttttcgtcgacg

>TnAV6a taatttgtgacatcgagcgaggactatcgatgtgtag**attta**aattactttgtatatgacttccacgaacagctaaaactttagctttttcaa**attta**ga

>CIV224 taaaactatcaccttatcaagaaaatctataaaga**attta**gaaagatga**attta**taaag**attta**attttaatgaccacggtcattaaa**attta**ttccaat

>CIV taaatttttaatgacaaaagtcattaagaattactgttttaactaattaaaataaaattgaacctgtgtaaataaagtcgttttaaaaaagaaaaatgga

>MIV taacccgccgtcgattcccaaggtaataaaaagatgaattattccgtaatctgggcaatcactatcctaattctagggcttgttttaaccttggcttggg

>DpAV4a AAGGCTAACTTCTCTAAAGCATTAGAAGTAGTGTTATCCGATCCCAAGACGGG

>HvAV3e tctggtaaattgctgcaaatgttccgcacgcaatgcgatagctctggcctttt (No palindrome found in the 150-bp)

>SfAV1a tcaagtgtcgcgttgacttgttcatccgcgtcgtctcccgctccggtcaattc

>TnAV6a caactttggcgcgtaattttgaaatatattcttttttttaatcaagatattgg

>CIV224 gaaaaattattcaatctgaattgaacagagtcttccatgttgctagggggggg

>CIV cgatttgattcattcgttaaaattaattcaaatagaaaaagaatctattttac

>MIV cacggcaaaaccctacccaccccatcaacccgctggtattaaactaccacacc

**Downstream region of DpAV4a ORF055**

>DpAV4a TAAACAGCGTCTCGATTTTATTTTATGCCTAAGGGCATAAAATAAATGTCTTCTACGTATTGTGGTAATAACCTGTTGAAAAACCCCAATAGGAGAGTGG

>HvAV3e tagctttgtcaaacgacacgacacgatacacaacagcagcagaccgattatacccaaaattaagagcgttacaagtgtcgccagagcaacgtaagtgggt

>SfAV1a tagtcgacacgacagcacgcatatcgatatagtcaagatgatgattatggtagctacaagggcggtacatgcggccgccgcgtacactaacatcggccaa

>TnAV6a tgatcttcatcatcatcatcatcattaatttttgttactttactcaatttggagagtttgctactaatgctacacattaatatgcatattattgatattg

>CIV taatccccgtctatcatt**attta**aaattagtctaaatcatcaattattggatctaagattgaaattatttcaccatcttctattaaaatattttttaatg

>MIV tgaccattgcacgcctaaaacaaccattaaatttttaatatttttttgtcaaatattaaaaattattaa**attta**aggttacggttgtaccttcaagtctg

>DpAV4a CGAGACTGACGTGTCTGAAGAAGGGGATCGGCATAGGCAAGAACCTGCCGACC

>HvAV3e atcggccatcgcttgtgcaccgtgtgtgcgacgttttgtacgagatccatcat (No palindrome found in the 150-bp)

>SfAV1a cgtttatgaacggtaaccgccaggtgtacggacaagtctagtatgaa**attta**t (No palindrome found in the 150-bp)

>TnAV6a gaataaatactaaaatgaaacccactccgagacatatacatatgattgtattg (No palindrome found in the 150-bp)

>CIV gtaacatttttaaaaataatcttaaattgattttaaataatatttttttaatt

>MIV ttgttcgcgttccttgcgcatttgtttggcaagttcgagtaccgtcaaaagac

**Downstream region of DpAV4a ORF064**

>DpAV4a TAGCGCATCATTGCCAAGAATAAAATCATAAGCGCTCCTATAGCGATCGCCACATACAACGCGGTGTTGGTGGGCGCCGACGGCGGAGAGACCGGCGGTG

>HvAV3e tgagttagttacagttttttcgggagctttgttagtttcacaaccatcatcggccaacaacgcacgaacggtactattgaatccattcggtgacacatgg

>SfAV1a tgagtagtattttcggtatgatgcaacagaaaaacgacgatgactcgggacggataggcgacaaccgcttggacacttcggagatgacgagcaagtgaaa

>TnAV6a taattgtgaagaggaagaagttgaatccacaattgccgctgctgatgataatgatactaatataaatgaagaaaaaggtacgacattaaataaaaatcag

>CIV taataacattaaaataattttaatgacttttaggtcattaaa**attta**agaattttcatc**attta**aaaaa**atttaattta**atgatctttatagagaagtct

>MIV taactctgcccaagacttgttgtggtaaagacttgttgtggtaaagacttgttgtggtaaagacttgttgtggtaaagtattgtggtgacttgaaacttt

>DpAV4a GAGCCGGCGCGGGCGAAGGCGCCGATGGAAACTGTGAACA**ATTTA**TGTAATTT (No palindrome found in the 150-bp)

>HvAV3e caggccttactaaagctggcgatttttggattactgaaagccacagctccgct (No palindrome found in the 150-bp)

>SfAV1a cagcacagacgttacggtcgactgcacgaacaacgcgccaacgtcttatcgcc (No palindrome found in the 150-bp)

>TnAV6a ggtacttcatctagtagtggtat**attta**gtggagaaaataatgttaaaataca (No palindrome found in the 150-bp)

>CIV aacaagatcgcctataatttggtaaatacaaaaattttttgagtccggattaa

>MIV taatttcgccaaagaaattaaaagcttaatctaaccttaatgcaccaaaaaaa

**Downstream region of DpAV4a ORF065**

>DpAV4a TAAACGTCCGGGCCCTTCATCATTCGCGGAGCGGACCCCGACGACACTGGCTGGTAAGTCTGCGTGGCGGGCGCTGTTTCTTTTTTAGTTATAGACAACA

>HvAV3e taacaaaatcgacgcaatgatcgaatcgtacgacgaaaatgtgctgtctacaccgtacataatggcattgcgcgaatacgtgttcggtgtaagaaagaac

>SfAV1a tagcgttgggcgtgatcgtgatagtgttcataatctattcgtacgtgacgaaatcgacgccatgatgatcgatacgtacgacgagaacgtactcgccacg

>TnAV6a tagaggtaagttttgtataaacgacgtgtctattaacggtcatgaggatattgatattaaaaatatacacaatacgattgaatgtttcaattcgaagagt

>CIV tagttttattagtttaaagaattaaaaggattaattaaactttatggaaaaacaaaaagataacgtaacttctactaaagaagagatattatgcactaaa

>MIV taggattcgtaatagctctggaggttgaaactctccactagagaaatatctatttttaaaattttaccattttaaaaaaagaacatccgcaccattattt

>DpAV4a GACTCAGGATATCGAAGTTGGTCTTCTTTGATATCGACTTGGCGACTATGAAC

>HvAV3e gtgggtctgttcataccaaaaaagtgcgtatcgtggtgcaaatggagacgcaa (No palindrome found in the 150-bp)

>SfAV1a ccgtacgtgatggctgtacgcgagtacgctttgggtgtacgaaagaacgtggg (No palindrome found in the 150-bp)

>TnAV6a ggatcgtcttcttcatcatctgtatataatgtgtgtaacataaccaagatgat (No palindrome found in the 150-bp)

>CIV aaagaacaaaacattgatatggaaaaatttaattcaattggatttttaaacga (No palindrome found in the 150-bp)

>MIV tgacggattgccggcgtcgttggattttgtccaccccattgccaagataataa (No palindrome found in the 150-bp)

**Downstream region of DpAV4a ORF070**

>DpAV4a TAATTTTTTTTTATAACACATTGTGTTATAAAAATGTCCCTAAACTATTTCGATAAAGTCTATAAAAAAGAATGGCTGTTGGGTGTTCCGGATCACGTCG

>HvAV3e taaaagagacacgtactcttggcgaatatttctgtatatttgcgtgctacagtttatccgtttgcattaatcaagacgacttgttttgaatggaacattc

>SfAV1a taatttttttcacattcttatcatatcactggggcggtggtgatcaaacgtctatttcgggatatatattcggttgttaccagagattgaagcacacagt

>TnAV6a taaattgaaacagcatcatgtgtgagagaatgttgctaaagttgaagcgcaacaatcttcgcgatcagttgagatttttaatacaattcgctaaagatag

>CIV taaaattctactagcattttaaaatataaaggttaaacaacctttatatttcaatt**attta**caaacatgcacttctaacgtaaggtgttccagtcaatcc

>MIV taagggcactacacgtaggcactgcacgcaagcaccaccagctcaatcgcaaccattttttttagataataaaactcgtccactttgaaagacatgcctc

>DpAV4a TCATCACCAAAGACGTCCTGTCTCTATTCGGTATAACAG**ATTTA**GAACCAGAG

>HvAV3e gcgggcgcgcacacacggaccatccgtctctgctgggataacaattagatatt (No palindrome found in the 150-bp)

>SfAV1a ttgtgagtgattcactggagagagtcgggaatcgtaaaacatggcaccatctt (No palindrome found in the 150-bp)

>TnAV6a tgatttgaa**attta**tgaatacctatgcacagcttaatgatgctagtggtggtc (No palindrome found in the 150-bp)

>CIV agttccacaatctccgtacgcggttcctaaagtaaaatatccagatccagacg

>MIV gggacaagaaactcgttcaccgtgccacgtccgacgtggaagatgaagacaat (No palindrome found in the 150-bp)

**Downstream region of DpAV4a ORF073**

>DpAV4a TAAAGACCCTGTTTATTTTTCAAACTAATTTGGTTTGAAAAATTACTGTTTGTACACCGACAGTTCGAACGTTTTGGGGTTGAGCTCGTAGAACTGCATG

>HvAV3e tgatcatcatcacattgacgtcgcgatccgcgttaataaaacgatttctgatgggttccagtcattcaagtccgacaccagtcattccgcaagttcctaa

>SfAV1a tagcgtgccccgtccatgccactaacgtaaaccttaatttcgtttaa**attta**caaatgggatcgagtcaaactaaatcgacaccggtaactgaagcgaaa

>TnAV6a tgattgtgtagaaaatttt**attta**ttcatcatactagtaagaaatacacagttatggtggtatttttgttaaca**attta**aacagttatcgtcattatcga

>CIV taaaaaattaaattttaatgaccattggtcattaaa**attta**aggtaaacacaagtgtgaaatctatgaaatactaacaatttcaaaaatatcttgaggaa

>MIV taaatctcaaatttcttttttaatgcttttattcaagcattaaaaacgtttaatcaaaacaatcgtcaccaccactactactacgaagagagacgactct

>DpAV4a AAGTCGTCTCCCACCGTCAAATCTTGAATGCGCCGTTTGAAACGCTGGAACGA

>HvAV3e aaaaactcaaactccggtcaatatactagcagacatagtcagtttgaagtaca (No palindrome found in the 150-bp)

>SfAV1a cgcgccacgcccgtgaacatagtcgcgaaactggtgtcgttaaagtacacggt (No palindrome found in the 150-bp)

>TnAV6a ggcacatacaatttttatttgagcataaagaacgagagtatcttctccacttt (No palindrome found in the 150-bp)

>CIV aaactgcatttgttatttttttattaaaccaagttgtattatcaataccaatg

>MIV tgggtggggtcacgttgtagtattcgataaacttggggtcattgtagggaatg

**Downstream region of DpAV4a ORF085**

>DpAV4a ATGTCCTGTTGGACATTAAAAAGAACTAAAGTCGGCCGTGTCGAAAAGTTTAGTCCGGGATTCGTTGTCGGCGCTCCGGTTGATCATCTTGTTGGCGCCC

>HvAV3e taacaaaattatacggtactttgtgaatgtcaagccagtttcgtgcatacacgaaactgacacgcaaaaagttattataatcacccggcggtgtgaattc

>SfAV1a tgatggtgcgacgtatgagcgcgttcgagaacagcgagcttcgtaagtcgcgttacatcaccgacttgctaaagttcgaatcggactttacgtccgacta

>TnAV6a tagtgggtgttggatataattgtgagtactatgtagtacgattaccagaatctcgtttactatgcttcaaagtggatcgaaatattgactgact*cacaa*a

>CIV taagtttttaatgccttcggcattaaaaactaataaaattattatacagaaccatataagaaaagaattttaataagttttttcaataagaataacatca

>MIV taagttttaatgggagcaattgctccattaaaattttaattctagtctttggttgtagcgcaccatattgcgcgtgtcagtcttgtcgtttttgggccac

>DpAV4a TCGTTGTACTTTTCCTGTATCTTTTCGAGTATCTCTTTTGGAGAAACCAAGAA (No palindrome found in the 150-bp)

>HvAV3e atcaaacttattacaacttcttaagttaggctgccatcaaagctagagtgtat

>SfAV1a ctacccgttcacgatggtcacgagcacggacaagtttgtcgtcaaaccacgac

>TnAV6a atttg**attta**acaaatatattacaagctaaagaattaagaggatacagagacg

>CIV aaaaattttttaaatatatcttgaatttctattcggttttcttcatc**attta**t

>MIV tcccagccgaaaattgaatttttcccaacttttaaggcgtaaataaacaacat

**Downstream region of DpAV4a ORF086**

>DpAV4a TAATGTCCAACAGGACATTAAATACTCTTAAACTTTACAAGTGTGTATGTCACGTCGGCTATTTTGTACACGACCAACGTTCTTCCGGAGCCCGGGCTCG

>HvAV3e tagtctattacgatgacaacactgcgaataacgtataaggcaatcaacacgagtattcctatcatgttttatactgtgctcgttggacatttgtaaaata

>SfAV1a taatctagaaacacgatgaaactgcgcaacgcatacatcacacccaaaacgatcaaaacgaacatggttttctttttctacaaacgtacgatccgtcggt

>TnAV6a tagaaaaataaaacaatgaagatatatttcataca**attta**ttattagcaagagttacaaaatcgtgtagaatgacacgagtatcgaagtttgatacaatt

>CIV taaaagcaacacaacaccaaacattaggcatttctgggataaaaacattataataaaaataaaatgagtattatagaaattattacagacagttccataa

>MIV taactgttacattttttatgcttgttgaaagcataaaaaatttttttattgtttcatggtcgattggttccttcttgttgtttttgtctttgctgctgca

>DpAV4a AAACAACGCTATATCCCGAAGAGTTCCAGAGGTAAAACGTATCCAACATGTTT

>HvAV3e tgtttgcgaaaccagagcccgcgatccgttccaggcccactgttttcctcgac (No palindrome found in the 150-bp)

>SfAV1a catttgtaaaacatgttctctacctcatcgaacgatgaaggtggtggtggtgg (No palindrome found in the 150-bp)

>TnAV6a tccgaaag**attta**tacaaatatcatcaatattactttgcactaattctaagga (No palindrome found in the 150-bp)

>CIV atacaacaacagatatcggaattgtta**attta**actcaaatagaacctttgtta (No palindrome found in the 150-bp)

>MIV ggtagagggttcgtttcaggataatactctccaagtgttggcgtcgctcttgt

**Downstream region of DpAV4a ORF089**

>DpAV4a TAAACCA**ATTTA**T**ATTTA**CTTTACTCGGCTAGCCGAGTAAAGTTCTAAGGGCGATCTCCCAGGAGCGCCAGTATCGCGTCTATCTTGCTCTCCAGCGCGT

>HvAV3e tagtaggcaaaaattttgtaatgtttgtaaataaataacgttaaatttgcaagttgtttattgtaattcctttttggtgaacgaatacgaaagtaatatc

>SfAV1a taaaattctttcgaatgtataaacgtttt**attta**caattatcataacgattacataacgatcaatacggagattagaaacaaaggtcttgaggtaaattg

>TnAV6a tgaccataaagcgaaacggatacaatgaatacatatcaatgagttccaattcgtcaccgttgttattgtatgatattattagtttaactaatggtagtaa

>CIV taattttttagaaaaagataggtctatagttctatggtttgttaaaattcttagttataagatttttattttaatggtataaaaccattaaaata**attta**

>MIV taagggcactacacgtaggcactgcacgcaagcaccaccagctcaatcgcaaccattttttttagataataaaactcgtccactttgaaagacatgcctc

>DpAV4a CGAGCCGGTGCACCAGGGTCTTGTCGGCCCGCTCCTTCTTCAGCTTGATGCGC

>HvAV3e gtcgactgaacctttgttctacaaaaagttggtactcaagtcgtaggtaggta

>SfAV1a ctgaaata*cacaa*ggtggtgtgaattcgacacaccgtttacgtcatcaatcca

>TnAV6a tgacaacgagataataataaattttcgcagtcatgtactaaatagtaatatta

>CIV aattaaaaa**attta**ctttaatacgtataaatagaagttaaatttgaacgtggt

>MIV gggacaagaaactcgttcaccgtgccacgtccgacgtggaagatgaagacaat (No palindrome found in the 150-bp)

**Downstream region of DpAV4a ORF093**

>DpAV4a TAAGCGATTTTTTACACACCCTTAGGGGTGTATAAAAATTGGATCTCGGTTGATATAAATGGTTATTATAAGAAACAAAGAAAGCGTCCCCTACGGGATC

>HvAV3e tgatctcgacgtggcgatgtttctaccgtagagcagcttcaccctataaaacaagtgacgtggtggctgcgtataagttgtgcgaagcatcgcgactacg

>SfAV1a tgacacacggacaacagaaagtattcgcgcacctccgggtccggaaacaggctgtccagaaagtcgttgacgaactttttcacatcgtccgccaactctt

>TnAV6a taaatgtgtatatatattaagcatcgcgtaaacgcgatgctatgtaaaaattgattttattttcataaatcatatgatattacatgagtgtgcatgtata

>CIV taaaagaaacgttggaaga**attta**atttggaaaataacgaagaaaatggcatgcctttattctaaaattttaa**attta**aaggttttttaacctttaaatt

>MIV tgatgcgacacgaaccacggcaatgacggtcccgacccctctctagagtcacttgggatgacaattttggtagaaaagaaccgtaataaacaatgtctca

>DpAV4a CTAAGCAATTTTTACGAAACAGAACTGGTTCTGGACGGTGAAAAGTGGAATAA

>HvAV3e cgatgcttcatctcggcgtgtgatctcatcatgtaaacgtaccaaaagaactt

>SfAV1a cgtacggcacgtagtcacagttcaaacacctgctgatcatatcttccggctta (No palindrome found in the 150-bp)

>TnAV6a tctgagagataactatcatgacttcgtcgtattgtctggaattcttgcgcccc

>CIV tatcaataatataataaatgtctttcaaaaaattaacaaacgtatggaatgta

>MIV cgtttcagtcattgcagcccgactgctggtttgggttggaattttactatgtt

**Downstream region of DpAV4a ORF103**

>DpAV4a TAATATAAAGATGTCTACTACGGCATACGCGGCGGTCGGCGACCGCATTTGGAATAAAAATAACCACCTGTTGGTTTATGTGATCGCAGCGAGAAACGCG

>HvAV3e tgaaggagattgaaagtcacacttagtgtggcgttgtacgtcacactaaacattatcgaaacaaagtctattgtcatactcaactctttattcgattaca

>SfAV1a tgatgatggatccaggtcgtgacgcgtgttcgaacatgatcggcaacctggaagccctcgagaggttcatgatcgaaaacgacgtcgtgtcgtacaaagt

>TnAV6a tgatgtggattgtatcggactacgtgatcgtagtccgaaatgaatattcatatggttcaaaatcttgaataaaagttggagatgaagtgtatttttaatc

>CIV taaactattctgtaagttaaaagattatattataaaatagagtaaaaatggataaatacacaatacgtcagtt**attta**tgt**attta**agtgataaccaata

>MIV tgaatttttaatttttctaaaaaaattaaaaataatggtttctcctagccttgggcgaggaacg**attta**cacagggattcgaggtcggcatctccggtgg

>DpAV4a GGGAACGTTTCTCCGGGCTCTTACAAGTTCTACGCGATCGAGGGGTTCGAGGC

>HvAV3e caagtacttgcataagtaacgttataattatcataaatttgcgcagaatacac

>SfAV1a gtcgagcgattacgcgttgtgtgacaacatgcgccgcacgatcactatcctgt

>TnAV6a agtactgatacagatatacaatgtttaagaagaatacatcttatgaaaatgta

>CIV tgtttattgtagaaaatgtat**attta**cacctttaaggggcg**attta**aacgact

>MIV tttgaaccttttccgtcaaaatgtcgagaaattttcgactcccgttcagggtg

**Downstream region of DpAV4a ORF108**

>DpAV4a TAAAAATGTTTTTCCGTATAATAAAATGGATCAATCGACTATGATTATTTTACTTATCGCCGGAGCCGCGTTGGCGTATTACCTTTACACCCAACAGAAC

>SfAV1a tgaaaagaaataaaaataaaaaacacgtctgttgccaaaaatactgtattttcattccgtccacctcttcccgcaacaactgcacactgcgaacaccgtc

>HvAV3e105 tgatcacaataaaaacgacacc**attta**attgcaagtatttgttttattcagttcactctttccattttttaccacatccactacacacggcgaacaccgt

>TnAV6a taataaagaacaagcagacgacggtgctatttggacgaaggctagctgtttcattaaatacatatatgaagaatcactacaattacaaaagccagaagta

>CIV taaccattatacaattttaatgactttacagtcattaaaattcattaaaattgaagttattaaaaaagaaagaaaaagaagaaaaggatacaatggactt

>MIV tagggtcccaattgtccatttttcacctcgtttacgagttggaag**attta**agaaaattgattgataattttaaattaaatggttaaagtacaccgtaaaa

>DpAV4a AAGAAACAGTAA**ATTTA**ATAGAACAACAAACAGTAA**ATTTA**TTAGAAGCTGTG

>SfAV1a atcggttcgtcacctccacgaacttgccgagcgaaagcgtaaatcttcctcga (No palindrome found in the 150-bp)

>HvAV3e catcggttcgtctccgccgcgcacttgacgggcaaaggcgtaaatttttctag (No palindrome found in the 150-bp)

>TnAV6a gaggaaggggctctacaatgtaacagatgtaattctcataaaattcattgtac (No palindrome found in the 150-bp)

>CIV ggctttaaaagttaaaattttaaaaatgctgtgtaaatacctcaccaataaaa

>MIV cgtaaaatgaatcctacaagcgctgattttgtcataccacccgcactggagac

**Downstream region of DpAV4a ORF116**

>DpAV4a TAAAGTTTCAATAATAAAAAATATTATTGAAACTAAAACCTAGACATGATTATGTTGACCACGTTCATCAGTTCGTTGTCGTACTGCGCGTCCTTATCAA

>HvAV3e taatcaaagcattcgacgacatcgccgactattagtcggcgatgtcatacctcggtaatatcgttatggtacttcgttacatcttttgagggtgtcac**at**

>SfAV1a taggttgcgccacggtatcgccgactgcagtcggcgattagcgcgtgattcgtgtgtcacggtacctcgttactgcggccgtcgtttcgattgcggaaat

>TnAV6a taaggtaatatatatatatatatatataatggattgaatgttattcaatccaaaaattgatta**attta**aaagaataattagcatcta**attta**gtgttgtt

>CIV taa**attta**aatacatttttataaaggtcaattgacctttataaaaaatttgtcttttcaacttaa**attta**cagataataacttttatgatttttttttgg

>MIV taaccgatggggcagggttaattttttaatttggatcaaaaattaaaaaatatggtcaactgttccttaaaaaaactgctgtcttgattctctaaaccct

>DpAV4a AAAAATAAAACGGTTTGATCTTTATGCAGTTGGCGGGTTGAGTGCGATAAACT

>HvAV3e **tta**gaacacatcttatgaatgttattcttgacaaatctatacccgaaatggta

>SfAV1a cgaccttccgtctgttgttcgccacgaacctatagccgaagtggtacatgacg

>TnAV6a aaatcacatcaattataatgttgtttaaatcactacccctcgatgtgcacaga

>CIV atcaacaataaaataaagaaaaatggcttcgtttaacattgaaaatccattac

>MIV caaaagtatggacaaattcagctttaactttttataactattaaaaaatgaaa

**Downstream region of DpAV4a ORF117**

>DpAV4a TAGTTTCAATAATATTTTTTATTATTGAAACTTTACACAACTCTTCGCCCCAACAGTATCTGAGTTCTCAACTCCTCGTATTTGTTGAAATCGAACACCA

>HvAV3e tagttgagcacatgagtcgcaacataaaatatacaacgagtacgcaaccaccatgtactttgacatttttcagtgtgtctgttacctcacatacgcatta

>SfAV1a tagttgagtgctaaacatccacataataaacgacattgaaattgaaccacaacatgtacgtggaatactttcagttattatgctatgtagcgtacatttt

>TnAV6a taattatcttgagtgtataaatataccatatattataagattattccattattattctgtaaccaacatgaagagaacgttgtttgttgttgc**attta**ca

>CIV taaaatttgttgaaacattaaatttgtcaaaac**attta**attatattaaaagaagaacgagaatgataaaatgattttaattttaactataatagtaggtt

>MIV taaatttttaatgctcaaccaaagcattaaaaacctacaagtctagaataaagcagcaaatgtttttaaagtcaaacaaaatcataaatgggccgacaat

>DpAV4a GTTGCTGGTACTCCTCTTCCGATATTTGTCCGTCGGCAATGAACAGGTTGTAT

>HvAV3e actgcgataagcatttgtattttaatgcaattcgatagaatgacgctcgttta (No palindrome found in the 150-bp)

>SfAV1a cacggcaataagtctatgtatattgattcaagtggaccgaatgactctcgtct (No palindrome found in the 150-bp)

>TnAV6a attgtaattatcattagctgctcggtgaatggcgatagttgtgaacaatcgga (No palindrome found in the 150-bp)

>CIV ttttaatctattttgtaactgcaaagcgttttaaattaaagcgtttaattttg

>MIV gtacgaccagtttttcgtctttaccgcaaccggaaccaacaatgggagagtta

**Size variations of the intergenic regions located at the 3’ extremity of each of the core gene in the 6 virus species**

1. Salem TZ, Turney CM, Wang L, Xue J, Wan XF, Cheng XW (2008) Transcriptional analysis of a major capsid protein gene from Spodoptera exigua ascovirus 5a. *Arch Virol* 153:149-162. [↑](#footnote-ref-2)
2. D'Costa SM, Yao H, Bilimoria SL. (2001) Transcription and temporal cascade in Chilo iridescent virus infected cells. *Arch Virol.* 146: 2165-2178; D'Costa SM, Yao HJ, Bilimoria SL (2004) Transcriptional mapping in Chilo iridescent virus infections. *Arch Virol* 149:723-742 ; Nalçacioğlu R, Marks H, Vlak JM, Demirbaĝ Z, van Oers MM. (2003) Promoter analysis of the Chilo iridescent virus DNA polymerase and major capsid protein genes. *Virology* 317:321-329 ; Nalçacioglu R, Ince IA, Vlak JM, Demirbag Z, van Oers MM (2007) The Chilo iridescent virus DNA polymerase promoter contains an essential AAAAT motif. *J Gen Virol* 88:2488-2494. [↑](#footnote-ref-3)
3. Suhre K, Audic S, Claverie JM (2005) Mimivirus gene promoters exhibit an unprecedented conservation among all eukaryotes. *Proc Natl Acad Sci U S A* 102:14689-14693. [↑](#footnote-ref-4)
4. Petit A, Rouleux-Bonnin F, Lambelé M, Pollet N, Bigot Y (2007). Properties of the various Botmar1 transcripts in imagoes of the bumble bee, *Bombus terrestris* (Hymenoptera: Apidae). *Gene* 390:52-66. [↑](#footnote-ref-5)
