# Supplementary material for: Symbiotic Virus at the Evolutionary Intersection of Three Types of Large DNA Viruses; Iridoviruses, Ascoviruses, and Ichnoviruses
Source: PLoS One. 2009 Jul 28;4(7):e6397. doi: 10.1371/journal.pone.0006397 (PMC2712680; doi:10.1371/journal.pone.0006397)
Supplement: Figure S8 — Phylogenetic analysis of core genes. S8a. Phylogenetic trees depicting the relationships between each of the proteins shared by all vertebrate and invertebrate iridoviruses, ascoviruses and DpAV4a S8b. Phylogenetic trees depicting the relationships between each of the proteins shared by all invertebrate iridoviruses, ascoviruses and DpAV4a S8c. Table summarizing phylogenetic relationships between core proteins shared by all vertebrate and invertebrate iridoviruses, ascoviruses, and DpAV4a S8d. Table summarizing phylogenetic relationships between core proteins shared by all invertebrate iridoviruses, ascoviruses, and DpAV4a (0.67 MB DOC) [file pone.0006397.s008.doc]

**S8 : Supporting Information 8**

**Symbiotic Virus at the Evolutionary Intersection of Three Types of Large DNA Viruses;**

**Iridoviruses, Ascoviruses, and Ichnoviruses**

Yves Bigot, Sylvaine Renault, Jacques Nicolas, Corinne Moundras, Marie-Véronique Demattei,

Sylvie Samain, Dennis K. Bideschi, and Brian A. Federici

**S8a. Phylogenetic trees depicting the relationships between each of the proteins shared by all vertebrate and invertebrate iridoviruses, ascoviruses and DpAV4a**

**S8b. Phylogenetic trees depicting the relationships between each of the proteins shared by all invertebrate iridoviruses, ascoviruses and DpAV4a**

**S8c. Table summarizing phylogenetic relationships between core proteins shared by all vertebrate and invertebrate iridoviruses, ascoviruses, and DpAV4a**

**S8d. Table summarizing phylogenetic relationships between core proteins shared by all invertebrate iridoviruses, ascoviruses, and DpAV4a**

**Methods of analysis :** The sequence alignments, determination of conserved blocks, and the calculation of the trees with the maximum likehood, neighbor-joining, and parsimony procedures were obtained using MABL facilities at [http://www.phylogeny.fr/phylo_cgi/downloads.cgi[[1]](#footnote-2)](http://www.phylogeny.fr/phylo_cgi/downloads.cgi). Similar results with the three methods were obtained for each of the 28 core ORFs. ORFs were separated in two sets (S8a&c and S8b&d), depending on the availability of protein homologues to root the trees in at least one species belonging to vertebrate iridoviruses, phycodnaviruses, mimiviruses, eukaryotes and/or bacteria. The GBlocks program was used to eliminate poorly aligned positions and divergent regions, and to avoid to misleading encryption deletions and insertions, as previously performed[[2]](#footnote-3). However, to avoid a too stringent block selection, smaller final blocks, positions with gaps within the final blocks, and less strict flanking positions were allowed. Here, only results obtained with the maximum likelihood method are shown. For each ORF, the tree is represented with the LTR probability[[3]](#footnote-4) at each node (ranging from 0, no statistical significance, to 1, elevated significance; values typed in red and bolded). Branch lengths were proportional to genetic distances that are scale by one bar, below each tree. For each used sequence which was extracted from database, is indicated the name of the virus and its accession number in database. DpAV4a sequences in these trees were named, DpAV4, DpAV4-ORFX, Diadromus_pulchellus_ascovirus_4a or query. CIV and DpAV4a sequences were respectively located with a red or a blue arrow in the first tree. Ascovirus sequences were located by green spots. Conclusions of these phylogenetic analyses were summarized in S8c and S8d.

**Remarks about the use of the core genes for evolutionary purposes in virology**: The use of the core genes is a powerful tool to define the evolutionary relationships between virus families[[4]](#footnote-5). Nevertheless, some observations can be misleading since the core genes, like many virus genes, may have been involved during evolution in lateral gene transfers between unrelated viruses. In the present paper, such a situation was encountered twice, with homologues of the proteins encoded by the DpAV4a ORFs 041 and 055. Indeed, results revealed that both ORFs were subjected to lateral transfers, direct or via an intermediate virus species, from an ascovirus to an asfarvirus for ORF041, and from an ascovirus to the *Heliothis zea* virus 1 (Nudiviridae) or its close ancestor, for ORF 055.

**S8a. Phylogenetic trees depicting the relationships between each of the proteins shared by all vertebrate and invertebrate iridoviruses, ascoviruses and DpAV4a**

**ORF001 DpAV4a DNA polymerase B (Ascovirus-Iridovirus-Phycodnavirus-Mimivirus)**

**ORF003 DpAV4a RNAseIII (Ascovirus-Iridovirus-Phycodnavirus)**

**ORF008 DpAV4a Major Core protein (Ascovirus-Iridovirus)**

**ORF019 DpAV4a Major Capsid protein (Ascovirus-Iridovirus-Phycodnavirus)**

**ORF020 DpAV4a SNF2 DEAD-like helicase (Ascovirus-Iridovirus-Phycodnavirus-Mimivirus)**

**ORF036 DpAV4a Serine/threonine kinase (Ascovirus-Iridovirus)**

**ORF040 DpAV4a Unknown function (AscoV-IridoV)**

**
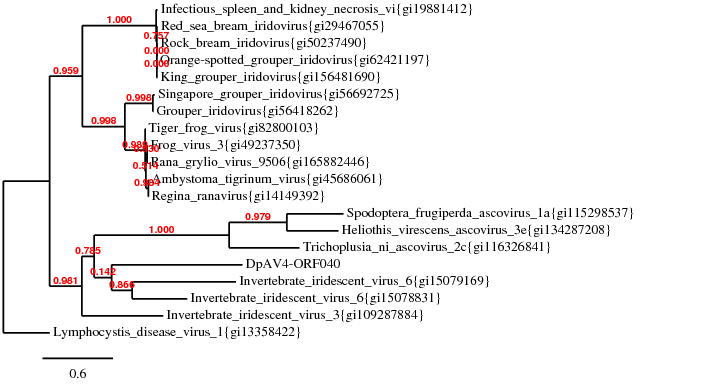
**

**ORF041 DpAV4a Thiol-oxidase (Ascovirus-Iridovirus-Phycodnavirus)**

**ORF043 unknwon function (Ascovirus-Iridovirus)**

**ORF055 DpAV4a Thymidine kinase (AscoV-IridoV-PhycodnaV-MimiV)**

**ORF064 DpAV4a Unknown function (Ascoirus-Iridovirus)**

**
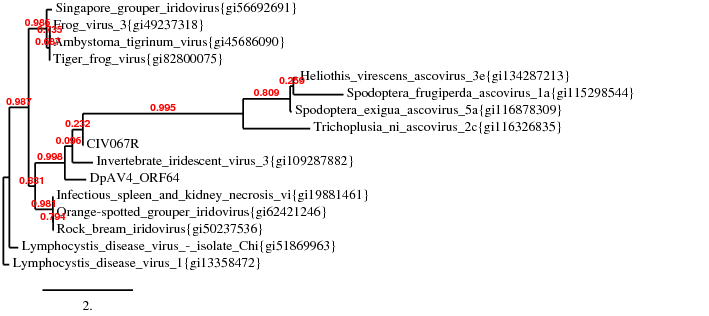
**

**ORF065 DpAV4a Myristylated membrane protein (Ascovirus-Iridovirus)**


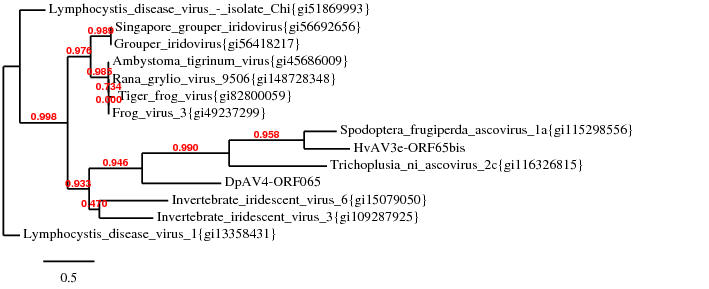


**ORF070 DpAV4a DNA-directed RNA polymerase (Ascovirus-Iridoirus)**

**
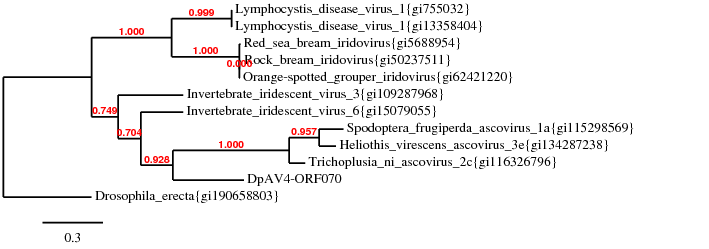
**

**ORF073 DpAV4a DNA-directed RNA polymerase beta subunit (Ascovirus-Iridovirus)**

**ORF085 DpAV4a Dynein-like beta chain (Ascovirus-Iridovirus)**

**ORF086 DpAV4a Dynein-like beta chain (Ascovirus-Iridovirus-Phycodnavirus)**

**ORF089 DpAV4a DNA-directed RNA polymerase subunit alpha (Ascovirus-Iridovirus)**

**ORF093 DpAV4a Putative ATPase (Ascovirus-Iridovirus-Mimivirus-Asfarvirus)**

**ORF103 DpAV4a Late translation factor VLTF3-like (Ascovirus-Iridovirus-Mimivirus-Phycodnavirus)**

**ORF117 DpAV4a CDT-like phosphatase (Ascovirus-Iridovirus)**

**S8b. Phylogenetic trees depicting the relationships between each of the proteins only shared by all invertebrate iridoviruses, ascoviruses and DpAV4a**

**ORF009 DpAV4a unknown function (Ascovirus-invertebrate iridovirus)**

Too few phylogenetic data were available in the sequence alignments performed with CIV, MIV, SfAV1a, HvAV3e and TnAV6a protein to obtain a significant tree, albeit the closest related protein has a BLASTp e value of about 10^-3^) and is encoded by ORF CIV359LL.

**ORF010 DpAV4a DNA-directed DNA polymerase II subunit Rbp5b-like (Ascovirus-invertebrate iridovirus)**

Too few phylogenetic data were available in the sequence alignments performed with CIV, MIV, SfAV1a, HvAV3e and TnAV6a protein to obtain a significant tree, albeit the closest related protein has a BLASTp e value of about 10^-3^) and is encoded by ORF CIV454R.

**ORF022 Yabby-like transcription factor (Ascovirus-Invertebrate iridovirus)**

**ORF026 Metallo-dependent calcineurin-like phosphatase (Ascovirus-Invertebrate iridovirus)**

**ORF033 Sbc subunit C (Ascovirus-Invertebrate iridovirus)**

Too few phylogenetic data were available in the sequence alignment performed with CIV, MIV, SfAV1a, HvAV3e and TnAV6a protein to obtain a significant tree, albeit that the closest related proteins encoded by ORFs MIV094L and CIV050L have elevated BLASTp e values (<10^-20^).

**ORF048 Cathepsin B (Ascovirus-Invertebrate iridovirus)**

**ORF108 DpAV4a Zinc finger protein (Ascovirus-Invertebrate iridovirus-Phycodnavirus-Mimivirus (probably lost at the origin of the vertebrate iridovirus evolution)**

**ORF116 DpAV4a Unknown function (Ascovirus-Invertebrate iridovirus)**

Too few phylogenetic data were available in the sequence alignments performed with CIV, MIV, SfAV1a, HvAV3e and TnAV6a protein to obtain a significant tree, albeit the closest related protein encoded by ORF CIV259R have elevated BLASTp e values (<10^-12^).

**S8c. Table summarizing phylogenetic relationships between core proteins share by all vertebrate and invertebrate iridoviruses, ascoviruses, and DpAV4a**

| **ORFs supporting the existence of the DpAV4a & the ascovirus** | **ORFs containing no decisive information** | **ORFs only supporting the existence of a single family, the ascovirus** |
| --- | --- | --- |
| 001  003  008  036  040  041  043  055  064  085  086  103  117 | 019  020  065  070  073  089  093 | - |

**S8d. Table summarizing phylogenetic relationships between core proteins share by all invertebrate iridoviruses, ascoviruses, and DpAV4a**

| **ORFs supporting the existence of the DpAV4a & the ascovirus** | **ORFs containing no decisive information** | **ORFs only supporting the existence of a single family, the ascovirus** |
| --- | --- | --- |
| 022  026  048  108 | 009  010  033  116 | - |

1. Dereeper A, Guignon V, Blanc G, Audic S, Buffet S, Chevenet F, Dufayard JF, Guindon S, Lefort V, Lescot M, Claverie JM, Gascuel O (2008) Phylogeny.fr: robust phylogenetic analysis for the non-specialist. *Nucleic Acids Res.*36 (Web Server issue):W465-9. [↑](#footnote-ref-2)
2. Stasiak K, Renault S, Demattei MV, Bigot Y, Federici BA (2003) Evidence for the evolution of ascoviruses from iridoviruses. *J Gen Virol* 84:2999-3009. [↑](#footnote-ref-3)
3. Anisimova M, Gascuel O (2006) Approximate likelihood-ratio test for branches: A fast, accurate, and powerful alternative. *Syst Biol* 55:539-52. [↑](#footnote-ref-4)
4. Iyer LM, Aravind L, Koonin EV (2001) Common origin of four diverse families of large eukaryotic DNA viruses. *J Virol* 75:11720-11734. [↑](#footnote-ref-5)
